# Stable intuition and the rise of deliberative prosociality in childhood

**DOI:** 10.1038/s41562-026-02487-4

**Published:** 2026-07-06

**Authors:** Francesco Margoni, Francesco Nava, Chiara Sotis, Matthew R. Levy, Valerio Capraro, Elena Nava

**Affiliations:** 1 Department of Psychology, University of Stavanger, Stavanger, Norway; 2 Department of Economics, London School of Economics, London, UK; 3 Department of Economics, Northwestern University, Evanston, IL USA; 4 Department of Psychology, University of Milano-Bicocca, Milan, Italy

**Keywords:** Human behaviour, Economics

## Abstract

We examine how social behaviours emerge and stabilize across childhood. A sample of 537 Italian-speaking children (3–10 years) were randomly assigned to respond under time pressure (intuitive) or time delay (deliberative) in social decision tasks (Public Goods, Dictator, Ultimatum, Deception and Moral Dilemmas). Factor analysis identified three latent dimensions: Prosociality (cooperative, altruistic and honest actions), Social Optimism (beliefs about others’ cooperation) and Acquiescence (tendency to accept offers). Intuitive responses were more prosocial than deliberative ones in early childhood (*β* = 0.66; 95% CI = (0.35, 0.97)), but this difference diminished with age (*β* = −0.11; 95% CI = (−0.18, −0.05)). We found no evidence that Social Optimism varied across age or decision mode, whereas Acquiescence declined with age (*β* = −0.14; 95% CI = (−0.19, −0.10)). These findings suggest a developmental shift whereby prosocial behaviour is initially driven by intuitive responses and gradually becomes embedded within reflective, deliberative decision-making systems, as cooperative dispositions stabilize across childhood.

## Abstract (web summary)

In children aged 3–10 years, intuitive responses lead to more prosocial choices in early childhood, while deliberate reasoning increasingly supports cooperative, fair and honest decisions with age.

## Main

Social decision-making is central to human associative life: cooperation, altruism and aversion to inequity are thought to contribute to the success of our species in coordinating small and large groups towards common goals^1–5^. In the current paper, we use the term ‘social decision-making’ to refer to a broad class of decisions that affect not only the decision maker’s welfare but also that of others^6^, and the term ‘prosociality’ to refer to voluntary behaviour that benefits others^7^. In particular, we analyse behaviour across several experimental paradigms: the Public Goods Game (PGG) (measuring cooperation), the Dictator Game (DG) (measuring altruism), the Ultimatum Game (UG) (measuring aversion to inequality), the Deception Game (DEG) (measuring honesty) and Moral Dilemmas (MDs) (capturing the choice between utilitarian and deontological courses of action). Within a dual-process framework, we compare the developmental trajectory of intuitive and deliberative social decision-making. Using factor analysis, we capture associations across different decisions and thus systematic patterns of behaviour. By focusing on development (3–10 years of age), the main aim of this study is to investigate how intuitive and deliberative social decisions arise and stabilize during early to middle childhood.

Dual-process accounts of decision-making propose that people’s decisions are determined by the interplay between two cognitive processes: fast and intuitive on the one hand, slow and deliberative on the other^8,9^. In recent decades, a fruitful area of research has sought to understand which behaviours are more driven by intuitive processes and which by deliberative processes^10–13^. Although tested primarily in adults, the dual-process framework has also been applied to study the development of intuitive social decision-making. Focusing on studies that manipulated response time (which provide stronger evidence than studies relying solely on correlational measures, such as refs. ^14,15^), some findings suggest early intuitive cooperation and altruism in children^16,17^. However, current evidence regarding the role of intuition in promoting prosociality is mixed^18,19^, and prior work has been limited to specific tasks and narrow age ranges, leaving the developmental trajectory of intuitive and deliberative social behaviours throughout early to middle childhood largely uncharted. Here, adopting a developmental approach to intuitive and deliberative responses, we provide evidence on the differential development of social intuitions and deliberative reasoning. In the initial formulation of this work, we sought to derive contrasting predictions from two prominent theoretical frameworks—the social heuristics hypothesis (SHH) and the nativist account. However, considerations arising during the revision process indicated that the present design does not permit a definitive dissociation between these accounts. Accordingly, we refrain from advancing strong a priori hypotheses and instead position our findings as constraining and informing both perspectives, a point we revisit in ‘Discussion’.

The SHH provides a framework for understanding how social experience might shape intuitive social behaviour. The SHH is a dual-process account of social decisions initially introduced to explain intuitive cooperation in one-shot and anonymous interactions among adults^20^. According to the SHH, individuals internalize cooperative heuristics because cooperation tends to be more advantageous than selfishness in everyday interactions, where it pays off in the long run given that everyday interactions tend to be repeated in stable groups. When participants are tested in the lab through one-shot and anonymous interactions, manipulations promoting intuitions make participants more likely to follow these cooperative heuristics. Consistent with the SHH, two meta-analyses indicate that promoting intuition generally enhances cooperation^21,22^, with this effect particularly pronounced in high-trust environments, arguably reflecting a stronger internalization of cooperative heuristics^23,24^. By contrast, deliberation makes people more likely to tailor their behaviour to the specific context at hand, and therefore deliberation may favour selfish behaviour, because this is optimal in the specific one-shot and anonymous interaction^12,23^.

Beyond cooperation, meta-analyses of experimental studies with adult participants indicate that intuition favours dishonesty when lying harms abstract but not concrete individuals^25^, promotes honesty when cognitive effort is required for payoff maximization^26–28^, increases both offers and the rejection of low offers in the UG^6^ and favours deontological judgements when utilitarian choices entail direct harm, but not when harm is merely a side effect^6,29^. Effects on altruism, trust and trustworthiness appear instead inconsistent between studies and sample populations, with meta-analytical effects not statistically different from zero^6,30,31^. While the SHH accounts for patterns in cooperation^21^, altruism^31^, inequity aversion^32^ and truth-telling^25^, it struggles to explain the full pattern of results across various social decisions. The generalized SHH (GSHH) was proposed to address these limitations^6^, positing that intuitive processing promotes internalized heuristics designed for self-preservation—prioritizing the avoidance of being harmed and guiding individuals to avoid actions that might provoke harm from others—while remaining agnostic about deliberation.

Nativist accounts, supported by infant and early childhood research, highlight the early emergence of social competencies and intuitions favouring prosociality. Research suggests that a rich social understanding and the precursors of social evaluation emerge remarkably early in development. Infants and toddlers exhibit expectations about others’ social behaviour, showing sensitivity to violations of several foundational principles^33^, including fairness^34^, harm avoidance^35^, ingroup support^36^, authority relations defining obligations for both leaders and subordinates^37^ and reciprocity^38^. Beyond these expectations, they also demonstrate preferences for fair over unfair agents and for individuals who give rather than take resources from others^39^. Moreover, infants and toddlers display rudimentary forms of spontaneous altruism, such as instrumental helping of unfamiliar adults^40,41^. However, prosocial behaviour appears to develop more gradually than social expectations or evaluative preferences; specifically, prosocial actions such as helping and sharing emerge first, while more complex cooperative skills appear later^42,43^. From a nativist perspective, early-emerging tendencies are argued to reflect an intuitive response system: before age 3, children possess limited cognitive control over their actions or thoughts^44^ and receive minimal input from their social environment^45^. Consequently, their representations, evaluations and behavioural inclinations are hypothesized to arise from core cognitive systems built by evolution that might continue to shape social decisions across development and into adulthood^45–47^.

In the current study, we test the developmental timeline for the emergence and stabilization of social heuristics favouring either a prosocial or a selfish response by administering laboratory tasks to a large sample of over 500 children aged 3 to 10 years. By looking at commonalities in play across a wide range of tasks, we investigate how systematic patterns of social behaviour emerge and develop over time, thus advancing our understanding of the dynamic interaction between intuitive social processes and deliberative reasoning.

Despite assigning different roles to environmental learning in the formation of intuitive responses, SHH or GSHH and nativist perspectives may nonetheless converge on a similar conceivable developmental trajectory. Both the SHH and the GSHH posit that internalized heuristics depend on the social contexts in which individuals operate. Early prosocial intuitions are likely to arise within family environments rich in cooperative, trusting and supportive interactions^48–51^. These frameworks can thus predict a pattern of continuity whereby early-formed prosocial heuristics remain stable over time, provided that children’s subsequent social environments resemble their early familial contexts. Likewise, nativist perspectives are consistent with prosocial intuitions that appear early in life and persist across development^52,53^, as they propose that core moral intuitions favouring prosociality, cooperation and social cohesion are evolutionarily ingrained, might emerge early and, if so, probably continue to undergird decision-making throughout later developmental stages^33,45–47,54^.

However, at least two further developmental trajectories are conceivable. Within the SHH or GSHH framework, heuristics may also shift during childhood as children transition through diverse social ecologies. As children grow older and become more exposed to broader social arenas—such as school settings—characterized by more competitive dynamics, greater peer comparison and less guaranteed reciprocity^55–57^, their heuristics may gradually become more self-serving. Finally, a third possible trajectory is that prosocial intuitions strengthen with age, as their repeated use across increasingly diverse contexts may consolidate and generalize them.

In the present study, stability between ages 3 and 10 would be consistent with the first possible prediction, common to SHH, GSHH and nativist perspectives, of enduring prosocial heuristics, whereas a decline or increase in prosocial intuitions with age would support the second or third trajectory, respectively. Finally, although the SHH and GSHH posit that social heuristics are acquired through experience and may emerge early, and the nativist perspective similarly holds that prosocial intuitions may emerge early, the precise developmental onset remains unclear, leaving open the question of when these heuristics or intuitions first manifest.

## Results

**Fig. 1:**
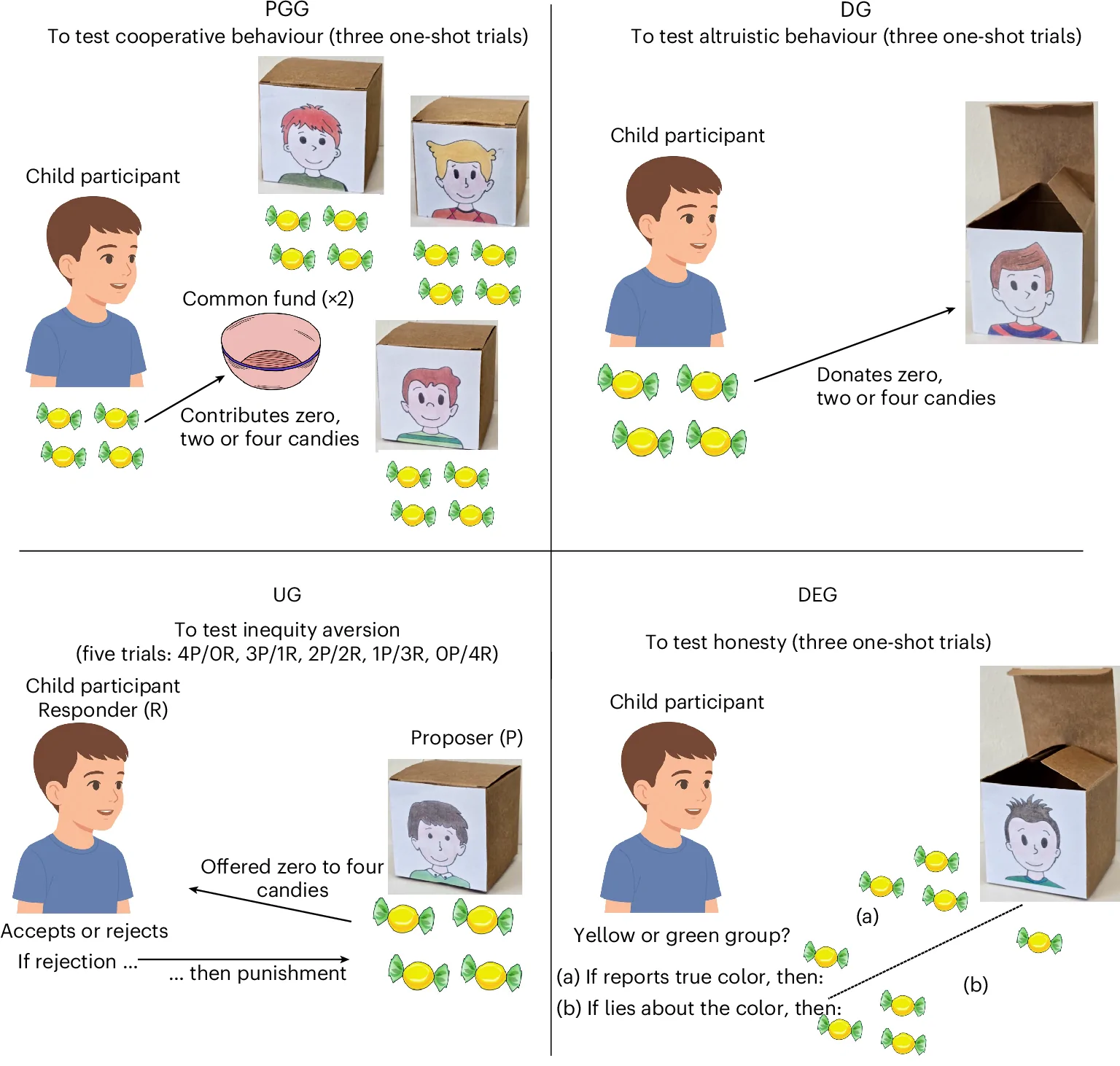
The four economic games used to test social decision-making. The figure displays the PGG, where children had to decide how many candies to contribute to a common fund, knowing that the total amount of contributed candies would be multiplied by two and equally distributed among the four children (top left); the DG, where children had to decide how many candies to donate (top right); the UG, where children had to decide whether to accept or reject a proposer’s offer (bottom left); and the DEG, where children had to decide whether to be honest or lie to increase their payoff (bottom right). Boxes are shown open when children contributed to the partner’s box (for example, the DG) and closed when the box contained candies already allocated by the partner (for example, the UG). Children were also presented with the Bystander and Footbridge versions of the classical trolley problem (for these child-friendly stimuli, see Supplementary Fig. 5).

A total of 537 children (ages 3 to 10; 254 female) participated in the study, completing five tasks under either time pressure (where they had to respond within 10 seconds) or time delay (where they had to wait at least 10 seconds before responding). Time constraints are commonly used experimental manipulations of cognitive processes^6,12,20,58^. The tasks included the PGG, measuring cooperation; the DG, measuring altruism; the UG, measuring aversion to inequality; the DEG, measuring aversion to lying; and two MDs, measuring utilitarian versus deontological judgements. Children played three rounds of the PGG with anonymous partners and had to decide how many candies (zero, two or four) to invest in a common fund, knowing that candies in the fund would be doubled and divided equally among players (Fig. 1). After each round, the children were asked to guess how many candies the other players had invested (expected cooperation). Children also played three rounds of the DG, where they had to decide how many candies (zero, two or four) to donate to a partner. In the UG, children played five rounds in the role of responder and had to accept or reject the offer of a proposer on how to divide four candies, knowing that rejecting the offer would result in no one receiving the candies. The offers they faced in these five rounds were 4–0 (that is, four candies for the proposer and zero for the receiver), 3–1, 2–2, 1–3 and 0–4. In the DEG, over three rounds, children were (privately) assigned to a group (the green or the yellow group), and when asked about it, they could tell the truth or lie, knowing that lying resulted in three candies for themselves and one for their partner, whereas telling the truth determined the reverse outcome. Last, children faced two MDs where they had to decide whether to harm one kid to save five other kids by redirecting a giant ball that was about to hit the five kids to the path where the lone kid was (Bystander dilemma (BD)) or by pushing the lone kid (who had a big backpack) off the bridge to stop the ball (Footbridge dilemma (FD)). In total, each child made 19 decisions. All decisions across tasks, except PGG expectations, were made under time pressure or delay. The partners were fictitious, but the children were consistently led to believe that they were real and were playing under the same conditions. Supplementary Table 1 shows that the age distributions of children under time pressure are similar to those under time delay. Supplementary Table 2 shows that compliance with time pressure was around 95%. As in previous studies using time pressure, we kept participants failing to comply with time pressure in the analysis^24,59,60^ and removed only those failing comprehension checks.

### Factor analysis

Rather than assuming that behaviour is governed by five distinct, game-specific dimensions, we let the data reveal whether underlying common character traits exist. To do so, we conducted a factor analysis to identify systematic behavioural patterns^61^, allowing us to assess within-participant variation across tasks. This approach improves precision by reducing idiosyncratic noise and accounting for correlations between behaviours. Once we identified character traits, we used linear regressions to examine how they are influenced by decision mode (fast or slow) and associated with age.

**Fig. 2:**
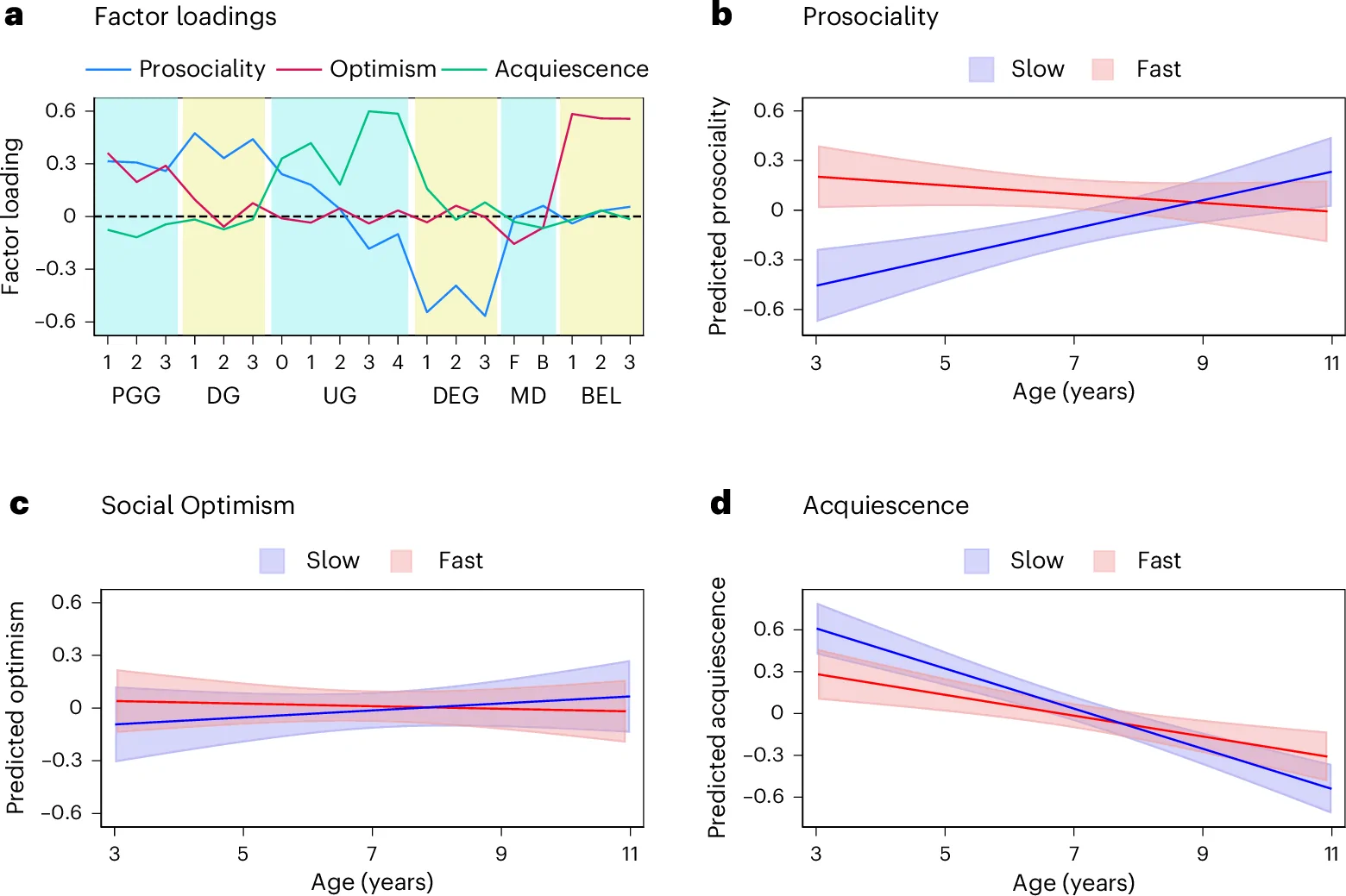
Factor loadings and differences in factors by age and decision mode. **a**, The factor loading for the three retained factors on contributions in the three PGGs, donations in the three DGs, the five UG decisions, deception in the three DEGs, judgements in the two MDs and beliefs elicited in the three PGGs (BEL). Shading in different colours is used to identify different tasks. **b**–**d**, The association with age estimated from linear regressions with factors as dependent variables (Prosociality (**b**), Social Optimism (**c**) and Acquiescence (**d**)) and decision mode as a control (*n* = 522). The data are presented as OLS predicted values (lines) ±95% CIs (shaded bands). All tests are two-sided, and statistical significance is based on sharpened FDR *q* values; the full test statistics, degrees of freedom, CIs and *q* values are reported in the main text and Supplementary Table 4.

Our factor analysis uncovered three significant latent factors (with eigenvalues higher than 1). On the basis of the factor loadings, we interpret these as Prosociality, Social Optimism and Acquiescence. Figure 2a and Supplementary Table 3 report factor loadings for the 19 decisions, with positive loadings indicating that behaviour in the task is positively associated with the factor, and vice versa for negative loadings. The first factor, Prosociality, is positively associated with contributions in the PGG, with donations in the DG and with acceptance of low offers in the UG, and negatively associated with deception in the DEG. This factor thus captures general prosocial tendencies, defined as behaviours that benefit others^7^. It therefore integrates cooperative and altruistic behaviours together with aversion to lying. It also includes accepting low offers in the UG, which increases the proposer’s and the collective payoff in one-shot interactions, by refraining from costly punishment. In this context, the factor primarily reflects a child’s inclination to benefit others and to forgive unfairness rather than a principled commitment to enforcing fairness norms. Although costly enforcement is often considered a key element of prosociality, because it promotes long-term cooperation, all tasks in our study involved one-shot interactions with putatively different partners. Punishments therefore do not affect long-term cooperation rates, and (unlike in repeated interactions) rejecting low offers here can only hurt others. The second factor, Social Optimism, is positively associated primarily with beliefs about the partners’ contributions in the PGG (assuming that partners decided under time pressure or time delay) and to a lesser degree with own contributions. Thus, this factor mainly captures children’s optimism that others will cooperate. Finally, the third factor, Acquiescence, is positively associated with acceptance of all offers (including low offers) in the UG. This factor thus captures not only the unwillingness to engage in second-party punishment but a more general willingness to accept others’ offers, whatever they are. Note that this factor is different from the opposite of inequity aversion, as inequity aversion would be associated with DG giving, while this factor is not.

### Decision mode effects and associations with age

Ordinary least squares (OLS) regressions were carried out to test the effects of decision mode (fast or slow) on the three retained factors (Prosociality, Social Optimism and Acquiescence) and to document their association with age. To account for the large number of hypotheses tested across specifications, we corrected all *P* values using the sharpened false discovery rate (FDR) *q*-values procedure of Benjamini et al.^62^, as advocated by Anderson^63^. All reported results and interpretations are based on corrected *q* values. The main analyses were carried out under the assumption that age is linearly associated with the factors and are presented in Fig. 2b–d. Supplementary Table 4 reports the regression coefficients. All OLS regressions report heteroskedasticity-robust standard errors, clustered by participant where observations are repeated within participants. All non-parametric tests are exact Wilcoxon rank-sum tests, which do not require the errors to be normally distributed. All statistical tests reported are two-sided. To assess the null results that we report, we replicated all OLS regressions as Bayesian linear regressions with Gaussian errors. We then report the Bayes factors computed by comparing the associated regression in which a variable was omitted to one in which it was present. Bayes factors larger than 1 favour the model with fewer independent variables, while Bayes factors smaller than 1 favour the model with more independent variables. Metropolis–Hastings sampling and flat priors were used. Specifically, we used flat priors to let the data primarily drive the posterior estimates, minimizing the influence of prior beliefs. All analyses were carried out using Stata v.18 (StataCorp).

Figure 2b presents the results for Prosociality. First, at age 3, intuition favours Prosociality (that is, Prosociality is significantly higher in the fast decision mode than in the slow decision mode; *β* = 0.66; *t*_518_ = 4.19; *q* < 0.001; 95% CI = (0.35, 0.97)). Second, deliberative Prosociality (that is, Prosociality under time delay) is positively associated with age (*β* = 0.09; *t*_518_ = 3.53; *q* < 0.001; 95% CI = (0.04, 0.13)). In contrast, intuitive Prosociality (that is, Prosociality under time pressure) displays a significantly weaker association with age than deliberative Prosociality (*β* = −0.11; *t*_518_ = −3.49; *q* < 0.001; 95% CI = (−0.18, −0.05)), and we do not reject a null hypothesis of no association with age for intuitive Prosociality (*β* = −0.03; *t*_518_ = −1.26; *q* = 0.120; 95% CI = (−0.07, 0.01)). The associated Bayes factor is 8.78, implying that the model excluding an association with age for intuitive Prosociality represents a better fit than the model including one. As shown in Fig. 2b, the gap in Prosociality between the fast and slow decision modes narrows with age, consistent with the significant negative interaction between decision mode and age reported above. Robustness checks using discrete age groups (3–4, 5–6, 7–8 and 9–10 years) corroborated these findings and further revealed that the association with age is not strictly linear. Prosociality is higher among children in the fast condition at ages 3–4 (*β* = 0.51; *t*_514_ = 3.14; *q* = 0.004; 95% CI = (0.19, 0.83)). Moreover, the difference between the fast and slow decision modes is significantly larger among 5–6-year-olds than among 9–10-year-olds (*β* = 0.41; *t*_514_ = 2.17; *q* = 0.038; 95% CI = (0.04, 0.79)), with no significant difference between decision modes remaining at ages 9–10 (*β* = −0.17; *t*_514_ = −1.35; *q* = 0.186; 95% CI = (−0.43, 0.08)); a Bayes factor of 1.75 suggests that a model without decision mode provides a better fit at this age.

As seen in Fig. 2c, we found no evidence that Social Optimism differs by age or decision mode (*F*_3,518_ = 0.26; *q* = 0.317). The associated Bayes factor is 1,247.65. Finally, as shown in Fig. 2d, we found that Acquiescence under time delay decreases with age (*β* = −0.14; *t*_518_ = −6.65; *q* < 0.001; 95% CI = (−0.19, −0.10)) and decreases at a lower rate under time pressure (*β* = 0.07; *t*_518_ = 2.31; *q* = 0.020; 95% CI = (0.01, 0.13)). While deliberation favours Acquiescence at age 3 (*β* = −0.33; *t*_518_ = −2.37; *q* = 0.018; 95% CI = (−0.60, −0.06)), the effect is no longer significant by age 6 (*β* = −0.12; *t*_518_ = −1.67; *q* = 0.064; 95% CI = (−0.26, 0.02)). The associated Bayes factor is 1.47.

**Fig. 3:**
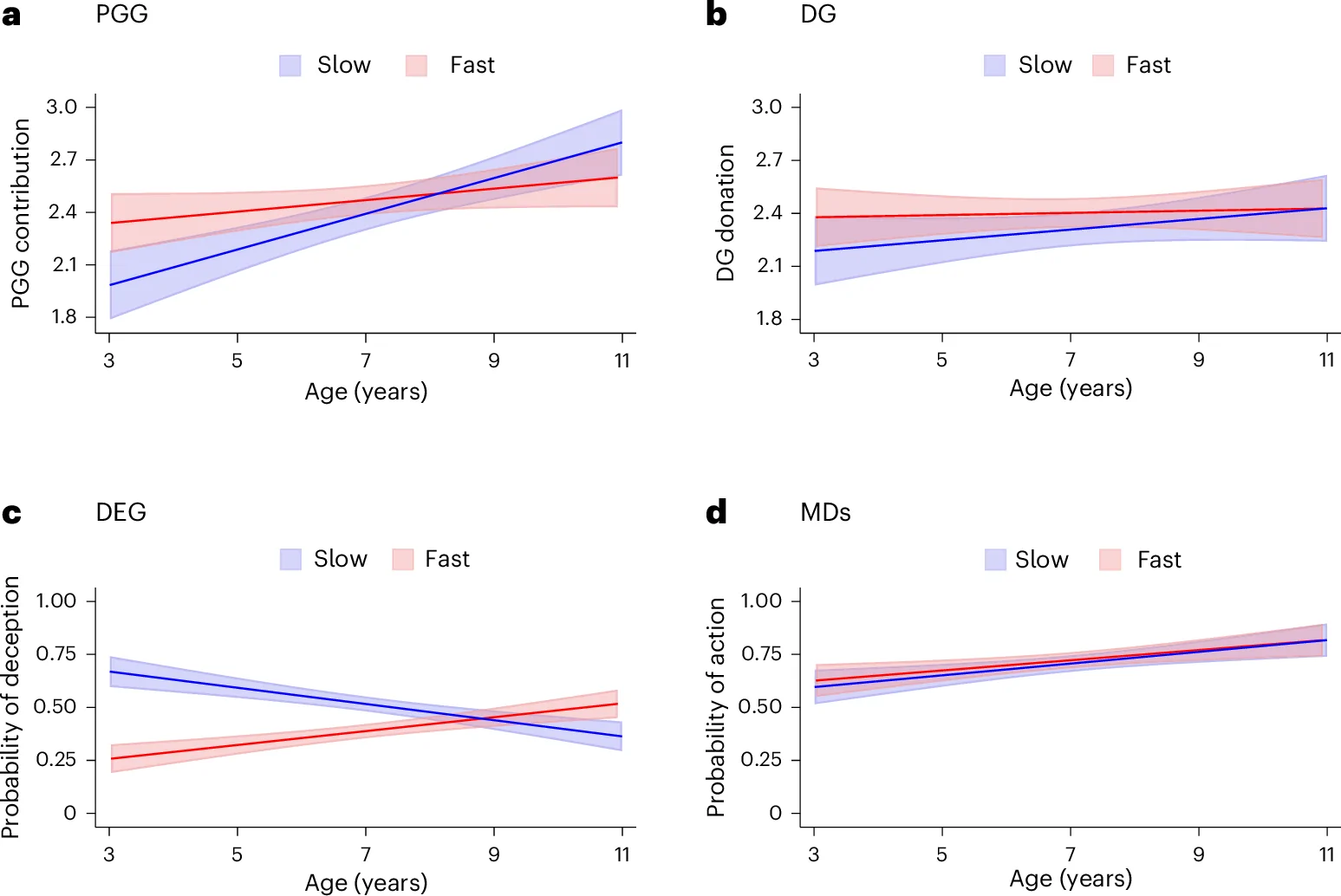
Age and decision mode differences in the PGG, DG, DEG and MD tasks. **a**–**d**, The association with age estimated from linear regressions with behaviour in the PGG (*n* = 1,572 observations; **a**), the DG (*n* = 1,575; **b**), the DEG (*n* = 1,575; **c**) and MDs (*n* = 1,047; **d**) as dependent variables and decision mode as a control. The data are presented as OLS predicted values (lines) ±95% CIs (shaded bands), clustered by participant. All tests are two-sided, and statistical significance is based on sharpened FDR *q* values; the full test statistics, degrees of freedom, CIs and *q* values are reported in the main text and Supplementary Table 5.

**Fig. 4:**
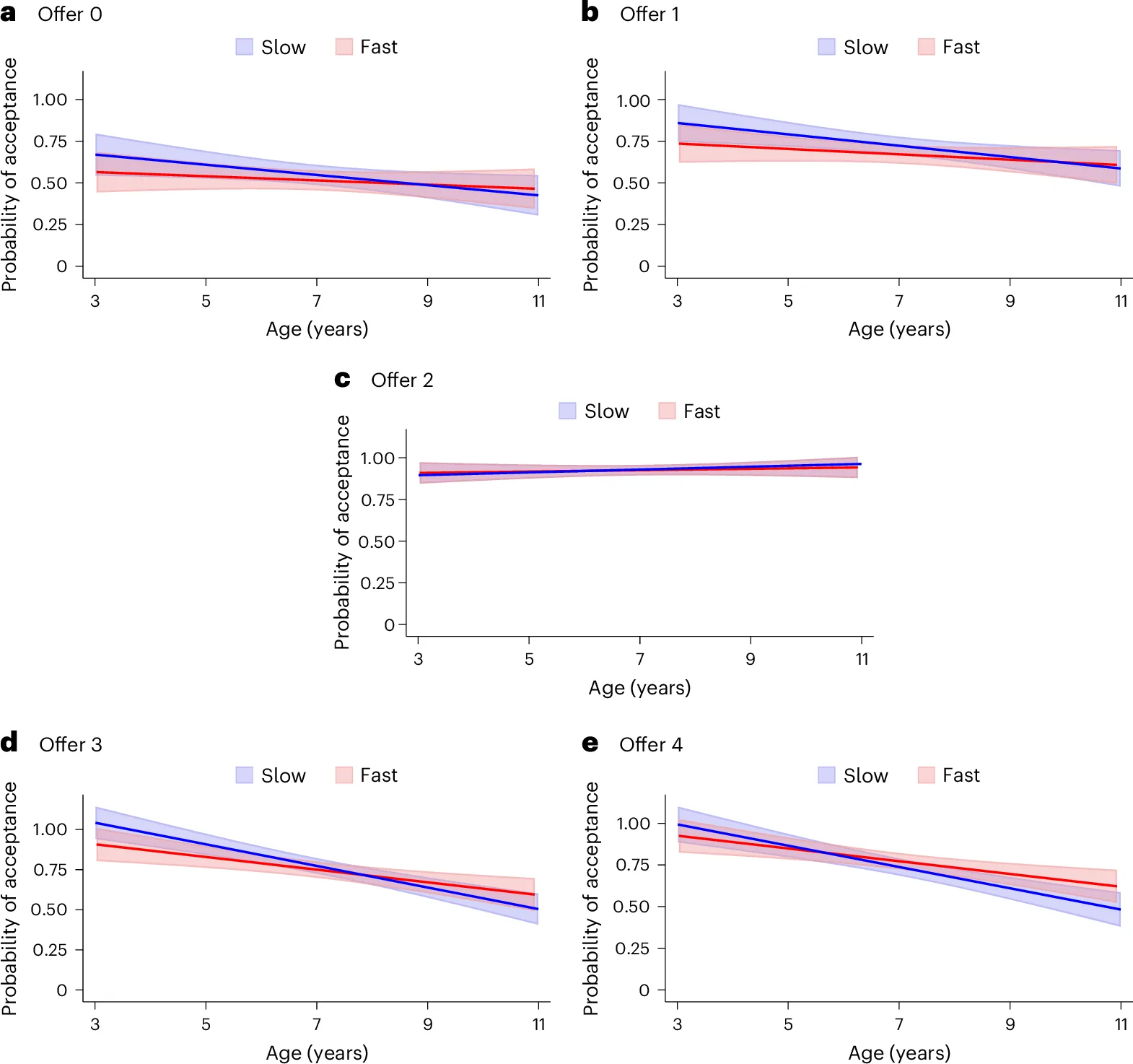
Age and decision mode differences on UG acceptance. **a**–**e**, The association with age estimated from linear regressions with behaviour in the five UG trials (4–0 (**a**), 3–1 (**b**), 2–2 (**c**), 1–3 (**d**), 0–4 (**e**)) as dependent variables and decision mode as a control (*n* = 2,625 observations, 524 participants). The data are presented as OLS predicted values (lines) ±95% CIs (shaded bands), clustered by participant. All tests are two-sided, and statistical significance is based on sharpened FDR *q* values; the full test statistics, degrees of freedom, CIs and *q* values are reported in the main text and Supplementary Table 5.

The results for each game separately are reported in Figs. 3 and 4 and in Supplementary Table 5 (see also Supplementary Figs. 3 and 4). In the PGG, the fast decision mode is associated with higher cooperation at age 3 (*β* = 0.34; *t*_523_ = 1.83; *q* = 0.049; 95% CI = (−0.03, 0.70)). There is no evidence of an effect of time pressure at age 6 (*β* = 0.14; *t*_523_ = 1.46; *q* = 0.071; 95% CI = (−0.05, 0.33)). The associated Bayes factor is 1.01, implying that, at age 6, a model without decision mode provides a better fit than one including decision mode; this is also the case at subsequent ages. Under time delay, contributions increase with age (*β* = 0.10; *t*_523_ = 3.65; *q* < 0.001; 95% CI = (0.05, 0.16)). Beliefs about others’ contributions are strongly associated with cooperation: children expecting higher contributions from others give significantly more themselves (*β* = 0.40; *t*_523_ = 5.21; *q* < 0.001; 95% CI = (0.25, 0.55)).

In the DG, we found no evidence that the fast decision mode favours altruism at age 3 (*β* = 0.19; *t*_524_ = 0.99; *q* = 0.156; 95% CI = (−0.19, 0.57)); a Bayes factor of 1.28 confirms that a model without decision mode provides a better fit. Under time delay, donations do not change significantly with age (*β* = 0.03; *t*_524_ = 1.02; *q* = 0.152; 95% CI = (−0.03, 0.09)), with a Bayes factor of 7.87 further supporting the absence of an age effect. In the UG, the probability of acceptance decreases with age (*β* = −0.04; *t*_524_ = −5.25; *q* < 0.001; 95% CI = (−0.05, −0.02)), while the fast decision mode reduces the probability of acceptance at marginal significance (*β* = −0.13; *t*_524_ = −1.81; *q* = 0.049; 95% CI = (−0.28, 0.01)); a Bayes factor of 99.06 suggests, however, that a model without decision mode provides a better fit.

In the DEG, the fast decision mode favours honesty at age 3 (*β* = −0.41; *t*_524_ = −6.12; *q* < 0.001; 95% CI = (−0.54, −0.28)), but we found no evidence of an effect at age 9 (*β* = 0.01; *t*_524_ = 0.35; *q* = 0.275; 95% CI = (−0.07, 0.09)); a Bayes factor of 14.22 confirms that a model without decision mode provides a better fit at this age. Under time delay, honesty increases with age (*β* = −0.04; *t*_524_ = −3.46; *q* < 0.001; 95% CI = (−0.06, −0.02)), whereas under time pressure the association reverses, with the interaction between age and the fast decision mode being positive and significant (*β* = 0.07; *t*_524_ = 5.07; *q* < 0.001; 95% CI = (0.04, 0.10)).

Finally, utilitarianism increases with age in both versions of the MD (*β* = 0.03; *t*_523_ = 2.83; *q* = 0.006; 95% CI = (0.01, 0.05)). Children are also less likely to act in the FD than in the BD (*β* = −0.19; *t*_523_ = −7.72; *q* < 0.001; 95% CI = (−0.24, −0.14)). Decision mode and its interaction with age do not provide evidence of an association with utilitarian choices (*q* = 0.264 and *q* = 0.297, respectively), and a Bayes factor of 445.74 provides strong evidence that a model excluding decision mode fits the data better than one including it.

### Robustness checks

Supplementary Section A3 replicates the analysis treating age as a discrete variable by grouping participants into four balanced age groups (Supplementary Table 6, and Supplementary Figs. 1 and 2). The results are robust, and the difference in Prosociality between the fast and slow decision modes is shown to be significantly larger among 5–6-year-olds than among 9–10-year-olds, thus replicating the main pattern obtained when age is modelled continuously.

Supplementary Section B1 establishes that our main results are unaffected by omitting 3–4-year-olds from the analysis, by including participants who failed comprehension checks and by excluding participants who did not comply with the fast condition (Supplementary Tables 7–12). Supplementary Section B2 shows that our conclusions hold when we exclude tasks with high uniqueness in the main factor analysis, and when we remove all PGG trials and the corresponding belief elicitation from the analysis (Supplementary Tables 13–16). Supplementary Section B3 establishes that for most tasks, the fast decision mode did not move the choices of 3–4-year-olds in the direction predicted by random choice (Supplementary Table 17). Finally, Supplementary Section B4 shows that our conclusions are robust to estimating separate factor weights for older and younger children (Supplementary Table 18).

## Discussion

This study examines how intuitive versus deliberative social decision-making emerges and stabilizes during early to middle childhood, by employing classic economic games and tasks designed to assess cooperation, altruism, inequity aversion, honesty and utilitarian versus deontological judgements. Our analysis revealed three significant latent factors. The first, Prosociality, is associated with cooperative and altruistic decisions in the PGG and DG, willingness to accept low offers in the UG and aversion to lying in the DEG. This factor captures a general attitude towards cooperative, altruistic and honest behaviour—that is, a prosocial tendency to benefit others^7^. This finding aligns with prior research in adults suggesting that different forms of prosocial behaviour tend to correlate, reflecting a general “cooperative phenotype”^61^ or disposition to “do the right thing”^64^. It also aligns with research suggesting that cooperation in social dilemmas is not necessarily linked to norm-enforcing punishment^61,65^. In other words, children who are highly cooperative in social dilemmas or prosocial (in the sense used here) are not necessarily inclined to punish norm violators, suggesting that cooperation and punishment may stem from separate mechanisms. Additionally, the absence of a correlation between the prosocial factor and deontological or utilitarian judgements mirrors findings in adults, which show no association between altruistic behaviour and the structure of moral judgements^66^, and no correlation between internalized moral identity and the strength of deontological versus utilitarian inclinations^67^.

The second factor, Social Optimism, reflects children’s expectations that others will act cooperatively. This trait is evident in the PGG, where children’s expectations of high contributions from their partners are positively associated with their own likelihood of contributing. This finding aligns with prior research showing a robust association between prosociality and expected reciprocity^1,68–73^, which we extend here to early childhood^18^. This association may reflect a foundational aspect of human social behaviour: individuals’ expectations about others’ cooperation and their own willingness to cooperate are closely intertwined, potentially influencing each other through mechanisms such as conditional cooperation^69^.

The third key factor that we identified is Acquiescence, which reflects the willingness to accept decisions made by others, even when those decisions are unfair. This is evident in the UG and in children’s acceptance of both low and high unequal offers. This finding highlights an aspect of children’s psychology that warrants further investigation: the capacity to tolerate or accept inequality in social situations. Whereas much of the focus has been on how children react to unfairness, our results suggest that some children are willing to overlook inequality, perhaps illustrating a nuanced understanding of fairness and social harmony.

Examining the impact of decision mode manipulations and how it varies across age reveals the following features of the development of the identified factors. First, as early as age 3, intuition favours Prosociality. This aligns with prior work showing similar effects with sharing^17^ but contrasts with evidence of reduced helping under time pressure^19^. This finding has implications for the two main theoretical perspectives outlined in the introduction to this paper. The nativist accounts claim that children are biologically predisposed towards intuitive prosociality^54,74^; however, the precise developmental timing of the emergence of these predispositions remains open to debate. Similarly, the SHH and GSHH frameworks can account for young children’s intuition favouring Prosociality, though, unlike nativist accounts, they attribute these tendencies to heuristics internalized following positive experiences in specific environments. This work reveals that intuition can favour prosociality as early as age 3 or 4. Future research, within the dual-process framework employed here, could further examine whether intuitions favouring prosociality progressively narrow or broaden over development by systematically manipulating the relational, physical and phylogenetic distance of interaction partners^75,76^.

Furthermore, we found no evidence of developmental changes in intuition favouring Prosociality. This aligns with continuity predictions from nativist accounts (positing that intuitions favouring prosociality might emerge early and persist across development) and is consistent with the SHH and GSHH if the school environment does not differ greatly from the early family context. We did, however, find that intuition no longer favours Prosociality among 9–10-year-olds, as deliberative Prosociality is higher in this age group than in younger children, and statistical tests did not provide evidence of a difference between deliberative and intuitive Prosociality in the same age group. Previous evidence from adolescents using a similar decision mode manipulation found that intuition favours selfishness in PGG decisions^73^. While the latter work examined only a subset of the games used in the current study, it suggests that intuition might favour self-interest later in development. Our data indicate that at least in our sample, such heuristics favouring self-interest, if they emerge at all, are not evident in children under 9–10 years of age.

Another key developmental finding is that deliberative Prosociality instead increases with age, closing the gap with intuitive Prosociality by ages 9–10. This trajectory for deliberative Prosociality aligns with classical theories of moral development, which emphasize that the ability to apply and reflect on abstract moral rules emerges only after several years of cognitive maturation^77^. More generally, prior studies indicate that around age 8, children show a marked advancement in prosocial behaviour, including heightened sensitivity to the social and reputational consequences of their actions and a stronger aversion to disadvantageous as well as advantageous inequality^78–81^. The present study, which explicitly manipulates deliberative versus intuitive decision-making, reveals that increases in prosociality are confined to deliberative reasoning. This finding suggests a reinterpretation of prior work, which typically assessed spontaneous, unconstrained decisions.

Our analyses also indicate a developmental divergence between the other two key factors. On the one hand, despite prior research that has reported a decline in optimism during childhood^82^, Social Optimism shows no evidence of an association with age and no evidence of an association with whether the partner is thought to decide under time pressure or with deliberation. In contrast, Acquiescence declines significantly with age under deliberation and at a lower rate under time pressure, perhaps reflecting either growing awareness of agency or simply a developmental shift towards more selective responding. This decline may correspond with the developmental emergence of domain-specific mechanisms, such as second-party punishment and heightened sensitivity to inequity, which become more prominent with age: as children’s cognitive and social frameworks become more sophisticated, their willingness to accept unjust outcomes may decrease, signalling a shift towards more norm-enforcing or justice-oriented reasoning^83,84^.

When we examined individual tasks, three findings emerged. First, we found evidence of intuitive cooperation in young children in the PGG, but no evidence of intuitive altruism in the DG. These results align with previous studies showing that while intuition may have a modest positive effect on cooperation^21,22^, it has little to no impact on altruism in adults^30,31^, and they only partially confirm prior work on intuitive social decision-making in children^16–18^. Moreover, no clear effect of promoting intuition is found on inequity aversion and deontological responses, effects that have instead been reported in adult meta-analyses^6,29^. Second, the largest effect of intuition is observed in the tendency of children to be honest. This result confirms work with adult populations showing that under certain testing circumstances, intuition favours truth-telling^26,28^. Third, cooperation, inequity aversion and utilitarian inclinations, though not altruism, show positive associations with age across both decision modes, which is overall consistent with developmental work in the field^83^. Honesty also increases with age, but only under deliberation; under time pressure the association reverses, with older children showing lower honesty than younger ones.

A number of limitations of the present study should be acknowledged. A primary concern is the extent to which participants, particularly the youngest, had the ability to understand the tasks. While we cannot directly test this concern, we can make the following observations. First, comprehension failures were very low overall, with only 2% of participants failing at least one comprehension check. Furthermore, as detailed in the robustness checks, our main conclusions are not driven by the youngest participants: the results hold when we omit 3–4-year-olds from the analysis, and the difference in Prosociality between the fast and slow decision modes is significantly larger among 5–6-year-olds than among 9–10-year-olds.

A second related concern is that time pressure might have exacerbated comprehension difficulties in the youngest age groups, thereby inadvertently influencing decision mode effects. We note, however, that instructions were always delivered without time constraints, deliberately aiming to avoid any interaction between time pressure and comprehension. This lack of interaction is reflected in several empirical findings. First, in most games, the fast decision mode did not move the choices of 3–4-year-olds in the direction predicted by random choice, which would be expected if low comprehension influenced the results. Second, as noted above and detailed in the robustness checks, the effect of decision mode on Prosociality by age holds when we exclude the youngest groups or the most demanding tasks.

Another limitation relates to the choice of decision modes. The present study does not include a time-neutral condition and can only assess behaviour under forced deliberation or forced intuition. The present study therefore provides evidence on developmental trends in these two frames, but not in a neutral one. Lastly, our sample consisted exclusively of Italian children. While findings from Western, educated, industrialized, rich and democratic^85^ populations may provide a starting point for comparison, prosocial development can vary substantially across cultural contexts^86–89^, and we therefore refrain from making broad generalizations, leaving open the question of how the current results extend to other populations. Moreover, our sample specifically comes from Northern Italy, a region that is known to differ in certain cultural norms, such as trust and social capital, even relative to other parts of Italy^90^.

In conclusion, by identifying three fundamental factors and examining their developmental trajectories, our study provides insights into how intuition and deliberation shape social decision-making throughout childhood. We found that intuitive Prosociality is present in younger children and remains stable with development. As children age, deliberation increasingly reinforces children’s prosocial behaviour. This developmental trajectory occurs with no evidence of changes in Social Optimism but alongside a decline in Acquiescence. Using a data-driven approach, this study identified underlying factors in children’s social decision-making, providing a framework for future research to examine how intuition and deliberation shape behaviour.

## Methods

Raw data and study materials (Movie-S1) are available via the Open Science Framework at https://osf.io/xpsqe/overview. The current study was not preregistered.

### Participants

A total of *n* = 537 Italian-speaking children participated in the study (age range = 3.01–10.99 years; mean = 6.97; s.d. = 2.29; 254 female). For each age group (3-year-olds, 4-year-olds and so on), approximately 70 children were tested, with half randomly assigned to respond under time pressure and half to respond under time delay (see Supplementary Table 1 for the exact number of children in each age group and decision mode condition). The sample size was determined by an a priori power analysis for a *t*-test (linear bivariate regression: two groups, difference between slopes), testing for an impact on a variable ranging from 0 to 4 (for example, the number of candies contributed in the PGG) over a seven-year range. To detect at least a ∣Δslope∣ of 0.15, with *α* = 0.05 and power = 0.99, a minimum sample of 532 participants was required. Participants were recruited from kindergartens and primary schools in Milano (Italy), serving a middle-income population, and were tested with their caregivers’ informed consent. Assent was obtained from all children: school-age children provided written assent following an age-appropriate explanation, whereas preschool children provided verbal assent. The participants received no monetary compensation for their participation in the study. Data collection took place between fall 2023 and spring 2024. The research was carried out in accordance with all relevant ethical regulations, and its procedure was approved by the Ethics Committee of the University of Milano-Bicocca (protocol number 0048825).

Decision mode was assigned at random, stratifying by age. Balance checks for condition assignment are presented in Supplementary Table 1. Participants who failed comprehension checks (12 across tasks) were excluded from the main analysis. In line with previous studies^26,59,60,91^, participants who did not comply with time constraints were retained in the analysis. Compliance rates for each task are reported in Supplementary Table 2. Blinding was not implemented in this study.

### Tasks and procedure

Children were administered five tasks under either time pressure or time delay. They were individually tested in a quiet room at school, playing with fictitious participants (believed to be real), and their responses were recorded by the experimenter using paper and pen. The order of presentation of the tasks was randomized.

#### PGG

To test cooperative behaviour, we used a child-friendly version of the PGG (for example, ref. ^92^), in which children were matched with three fictitious child participants (depicted with drawings on small boxes, as shown in Fig. 1). Each child played three one-shot games, each with different partners. On each trial, participants received four candies, knowing that the other players received the same. They decided how many candies to contribute to a common fund (a bowl), where they could insert zero, two or four candies, as the candies were taped together in pairs. The children were told that the candies in the fund would be doubled and equally distributed among all players. The selfish optimal strategy is to contribute nothing, while the maximal payoff occurs when each player contributes four candies.

The participants were instructed that all players would receive four candies, that each player would decide how many candies to contribute and that the candies in the fund would be doubled and shared equally. The children watched a three-minute explanatory movie (Movie-S1; see also Supplementary Section C1), followed by two comprehension probes. With four candies, the bowl and three closed boxes (representing the three partners), the experimenter explained, ‘See, in this game you can keep all 4 candies, put 2 in the common bowl for everyone and keep 2 for yourself, or put all 4 in the bowl.’ The experimenter then asked, ‘What happens if you keep all the candies for yourself? Do you take them home or do they go to everyone?’ (first probe) and ‘What happens if you put the candies in the common bowl? Do you keep them or do they go to everyone?’ (second probe). Children who failed at least one probe were given the instructions and probes again. Data from four 3-year-olds who failed the probes after three attempts were excluded from the analysis.

After the probes, the first trial was administered with a bowl, four candies and three closed boxes (each with a child’s face, male for male participants and female for female participants). The experimenter repeated the instructions (the script is provided in Supplementary Section C2) and clarified that the other players had already decided how many candies to contribute (but their boxes were closed, and the contribution could not be seen) and that no one would know what the others did, as the choices were anonymous and the partners were from a different school.

Half of the children were randomly assigned to respond under time pressure, where they had 10 seconds to answer, and the other half to respond under time delay, where they had to wait 10 seconds before answering. In the fast decision mode, the experimenter explained, ‘There is one last rule that I haven’t told you yet, but it’s very important. You have to decide right away! You only have 10 seconds to decide whether and how many candies you want to put in the common bowl, so you must be quick. Do you know how long 10 seconds is? I’ll show you!’ The experimenter then played a 10-second countdown and encouraged the child to make a decision. In the slow decision mode, the experimenter used the countdown to show how long to wait before deciding: ‘You must wait at least 10 seconds and think carefully; you can’t choose right away. After that, you can take all the time you need. I’m in no hurry; think carefully about what to do!’

The participants completed three trials. After each trial, they were not told how many candies the other players had contributed. Instead, they were told that they had to play a new round. All candies were removed, and four new candies were provided. At the end of the task, the children were informed that they could take home all the candies (those not invested, those received from the common fund and those accumulated in other tasks). In reality, each child received 20 candies in total, on the basis of the average number of candies one could accumulate across the PGG, DG, UG and DEG tasks. The children were then debriefed in age-appropriate terms, informed that the partners were fictitious and thanked for their participation.

#### Expected reciprocity

To examine expectations about the partners’ cooperative behaviour, the children were asked to estimate how many candies each partner had contributed. After each trial, for each partner, they were asked, ‘In your opinion, how many candies did this child put in the common bowl? None, 2, or 4 candies?’ These judgements were made without time constraints, and children in both conditions had sufficient time to reason about their partners’ likely contributions. The goal was to evaluate children’s beliefs about how others would behave under time pressure or delay, rather than their own pressured decision-making.

#### DG

To test altruism, we used a child-friendly version of the DG. Children were matched with a fictitious partner (as in the PGG) and completed three trials, each with a different partner (represented by an empty open box). The children received four candies and decided how many to donate (zero, two or four candies).

The participants were told that the four candies were all theirs to decide what to do with and that the partner would not know who played with them and could not reciprocate. The experimenter explained, ‘You and this child are playing this game. Here’s his box. Now I’ll give you 4 candies. These are yours, and you can decide how many to give him. If you give him candies, I’ll put them in his box, and he won’t know it was you, so he can’t give anything back. You can also keep all the candies for yourself, if you want.’ The experimenter continued, ‘For example, I can give him all 4, leaving me with nothing. Or I can give him 2, so we both have 2. Or, I can keep all the candies for myself. You’re the only one who decides what to do!’

Children then received a comprehension probe: ‘What happens if you put some candies in this kid’s box? Do both of you get them, or does he take them home?’ If the child failed the probe, the instructions were repeated up to three times. Data from children failing after three attempts were excluded; three 3-year-olds were excluded. Children were randomly assigned to the fast or slow condition, with instructions similar to those in the PGG (Supplementary Section C2). After each trial, the experimenter closed the partner’s box and told the child that the partner would later receive the candies. The participating child’s candies were then collected in a plastic bag.

#### UG

To test negative reciprocity and advantageous inequity aversion, we used a child-friendly version of the UG. On each trial, children were matched with a different fictitious partner and assigned to the responder role. They decided whether to accept or reject an offer made by the proposer, who had four candies to divide. The child could accept the offer, with candies divided as proposed, or reject it, resulting in no candies for either player. Each child completed five rounds with different offers: two low offers (4–0 and 3–1, with the numbers denoting the proposer–receiver split), one equitable offer (2–2) and two high offers (1–3 and 0–4). The proposer’s box was opened to reveal the offer, and the child was asked to accept or reject it. The trial order was randomized.

To explain the game, the experimenter pointed to the proposer’s box and said (here using the 2–2 offer as an example), ‘When I met this child, I gave her 4 candies to divide between her and you. Here’s what she did: 2 candies for herself, 2 for you. You can accept, say yes, and take your 2 candies, or reject and no one gets anything. If you say no, I will take all the candies and put them away. No one takes the candies. You decide freely, and the other child won’t know who played with her.’

The children then received a comprehension probe: ‘What happens if you say no? Do the candies go to someone, or are they put away?’ If the child failed, the instructions were repeated up to three times. After a correct response, the experimenter also reminded them, ‘What happens when you say yes?’ Data from children failing after three attempts were excluded, with one 3-year-old excluded. Children were randomly assigned to the fast or slow condition (see Supplementary Section C2 for the details). After each trial, the experimenter either set aside the candies if the offer was rejected or distributed them if accepted, then closed the proposer’s box and collected the remaining candies in the child’s bag.

#### DEG

To test aversion to lying, we used a child-friendly version of the DEG. Children completed three trials, each with a different fictitious partner. They were told there were two groups, yellow and green, one for the participant and the other for the partner. The participants discovered which group they were in by drawing a card, a private piece of information that the partner did not know. When asked about their group, they could either tell the truth or lie. The game’s payoffs incentivized lying: truth-telling resulted in one candy for the participant and three for the partner, while lying reversed the allocation.

To explain the game, the experimenter showed the child a partner (as in the DG, with the partner’s box open) and two cards (yellow and green, face down), saying, ‘In this game there are 2 groups, yellow and green. We don’t know yet which group you’ll be in, but this child here will be in the other group. Soon you’ll find out your group, and I’ll ask you to tell me. But be careful: if you tell the truth, you’ll get 1 candy and the other child will get 3. If you lie and say the other color, you’ll get 3 candies and the other child will get 1!’ The experimenter gave an example (the script is provided in Supplementary Section C2).

Next, the children were asked two comprehension probes: ‘How many candies do you get if you tell the truth? How many does the other child get?’ and ‘How many candies do you get if you lie? How many does the other child get?’ If the child failed at least one probe, the instructions were repeated up to three times. Data from children who failed after three attempts were excluded, with six 3-year-olds and one 4-year-old excluded. Children were randomly assigned to the fast or slow condition and instructed accordingly (see Supplementary Section C2 for the details). After each trial, the experimenter placed the participant’s candies in a plastic bag and put the partner’s candies in their box.

#### MDs

To test utilitarian versus deontological judgements in moral decision-making, we used a child-friendly version of the classic trolley problem (the materials were developed by Dworazik et al.^93^). The participants were presented with two dilemmas: the BD and the FD. In both, the participants did not play with partners as in the other tasks, but had to decide whether to harm one child to save five others from a giant ball rolling down a hill. In the BD, the protagonist can throw a fence across the main trail to redirect the ball onto a different trail where one child would be hit. In the FD, the protagonist can stop the ball from hitting five children by pushing a child with a backpack off a footbridge.

The scenarios were presented via animations on a laptop, using male characters for male participants and female characters for female participants. The order of presentation was counterbalanced across participants. The experimenter instructed the children that they would hear stories and decide the endings (the instructions are provided in Supplementary Section C2). In the BD, the story involved Mary/Tom walking down a hill when a giant ball is heading towards five children. The protagonist can push a fence to deflect the ball onto a trail where another child would be hit. The participants were asked, ‘What should Mary/Tom do? Push the ball to the side or not?’ The FD was similar, but the protagonist, Luisa/John, is on a bridge. They can stop the ball by pushing a child with a backpack off the bridge, saving the five children but harming the pushed child. The test question was, ‘What should Luisa/John do? Push the child onto the trail or not?’ (Supplementary Fig. 5 shows the animations, and the full script is in Supplementary Section C2).

For each story, the children answered two probes: ‘Do the 5 children see the ball rolling down the hill?’ and ‘Does this child (on the other trail or with the backpack) see or hear the ball?’ If the child failed at least one probe, the experimenter repeated the story and question up to three times. Data from children failing after three attempts were excluded. FD data from one 3-year-old and one 4-year-old were excluded. Children were randomly assigned to either the fast or slow condition.

### Reporting summary

Further information on research design is available in the Nature Portfolio Reporting Summary linked to this article.

## Supplementary information

Supplementary Information Supplementary Figs. 1–5 and Tables 1–18.

Reporting Summary

Peer Review File

## Data Availability

The dataset generated for the current study is available via the Open Science Framework repository at https://osf.io/xpsqe/overview.
