# Supplementary material for: Stable intuition and the rise of deliberative prosociality in childhood
Source: Nat Hum Behav. 2026 Jul 6;10(9):1686–97. doi: 10.1038/s41562-026-02487-4 (PMC13590409; doi:10.1038/s41562-026-02487-4)
Supplement: Supplementary file 1 [file 41562_2026_2487_MOESM1_ESM.pdf]

nature human behaviour

Supplementary information https://doi.org/10.1038/s41562-026-02487-4

Stable intuition and the rise of deliberative prosociality in childhood

In the format provided by the authors and unedited

Supplementary Information for
Stable Intuition and the Rise of Deliberative Prosociality in Childhood

Francesco Margoni[1], Francesco Nava[2], Chiara Sotis[3], Matthew R Levy[2], Valerio Capraro[4], Elena Nava[4]

[1]Department of Psychology, University of Stavanger.
[2]Department of Economics, London School of Economics.
[3]Department of Economics, Northwestern University.
[4]Department of Psychology, University of Milano-Bicocca.

Contents

A Additional Analyses **2**
A.1 Balance Checks and Condition Compliance 2
A.2 Main Tables and Results by Task 3
A.3 Non-Linear Age Regressions 4

B Robustness Checks **12**
B.1 Sample Inclusion Criteria 12
B.2 Task Selection Criteria 16
B.3 Time Pressure and Random Choice 19
B.4 Estimating Factors by Age 20

C Additional Scripts For Each Task **21**
C.1 The PGG Task (Explanatory Movie-S1) 21
C.2 Additional Scripts and Instructions 22

A Additional Analyses

A.1 Balance Checks and Condition Compliance

As shown in Supplementary Table 1, condition (fast, slow) assignment was roughly equal. There were slightly more children in the fast condition in the 3-4 year age group. This difference arose because 9 children in the 3-4 age group responding under time delay failed at least one comprehension check (and were therefore dropped from the main analysis), while 3 children in this group responding under time pressure failed. No participant failed comprehension in the other age groups.

Age Group	Age 3-4	Age 5-6	Age 7-8	Age 9-10	Total	Percent
Slow Condition	56	70	65	66	257	48.95
Fast Condition	63	70	67	68	268	51.05
Total	119	140	132	134	525	100
Percent	22.67	26.67	25.14	25.52	100	–

Supplementary Table 1: Sample divided by age group and decision mode.

Supplementary Table 2 illustrates compliance with the fast condition across experimental tasks, reporting the *percentage of non-compliant participants* for each task and iteration. Moral dilemmas were the tasks where participants struggled the most to comply with the condition. For the MD, any participant failing to comply with the condition in one of the two frames was recorded as having failed the condition in both frames due to data collection.

Task	PGG	DG	UG	DEG	MD
Round 1	2.66	3.42	–	5.34	–
Round 2	3.02	3.42	–	1.52	–
Round 3	2.66	3.80	–	1.90	–
Offer 0	–	–	0.38	–	–
Offer 1	–	–	0.00	–	–
Offer 2	–	–	2.66	–	–
Offer 3	–	–	1.90	–	–
Offer 4	–	–	1.52	–	–
Average	2.78	3.55	1.29	2.92	5.90

Supplementary Table 2: Condition non-compliance.

A.2 Main Tables and Results by Task

Supplementary Table 3 reports the factor loadings for the 3 retained factors, along with the uniqueness coefficients for each. We retained factors with eigenvalues greater than 1. The uniqueness coefficients are relatively high compared to other studies on adult samples, which is consistent with a higher degree of idiosyncratic behaviour in children. The aggregation through factor analysis allows us to extract commonalities across tasks. We show in section B1 and B2 that results are robust to a number of sample and task selection criteria, and in section B4 that results are robust to estimating factor weights separately for younger and older child participants.

Supplementary Table 4 presents the linear regression coefficients from our main analysis. In these regressions, the dependent variables are the retained factors with condition (fast, slow), age, and their interaction included as control variables.

Supplementary Table 5 presents the linear regression coefficients by task. In these regressions, the dependent variables represent the responses in a specific task, with condition, age, their interaction, and a round/trial indicator included as control variables. Specifically, the regressor *round* X identifies the fixed effect associated with playing round X relative to round 1. Since participants were required to accept 5 offer levels in the UG, the regressor *offer* X identifies the fixed effect associated with accepting offer X relative to accepting the fair offer (offer 2). In the MD, where participants had to play 2 frames, the regressor *footbridge* identifies the fixed effect associated with playing the footbridge frame relative to the bystander frame. The independent variable *beliefs* represents a participant's beliefs about the contributions of others in the PGG.

Public Goods Game

The findings from the PGG reveal several key patterns. At age 3, children in the fast condition contribute more than those in the slow condition. However, this effect disappears after the preschool years. Older children tend to contribute more than younger ones, and on average, children contribute significantly more than 2 candies, the fair split (see also section B3). Contributions also increase in the second trial. Additionally, children's expectations are positively correlated with their own contributions.

Dictator Game

In the DG, we find no evidence that condition or age are associated with children's choices. Nonetheless, children, on average, donate significantly more than 2 candies, the fair split (see also section B3). Donations also increase in the second and third trials compared to the first.

Ultimatum Game

The findings from the UG indicate that older children are less likely to accept offers than younger ones. Children are most likely to accept fair offers (worth 2 candies), but they are more likely to accept higher offers (worth 3 or 4 candies) than lower offers (worth 0 or 1 candy). Fast decision mode is associated with lower acceptance at the margins, $q = 0.049$.

Loadings	Prosociality	Social Optimism	Acquiescence	Uniqueness
PGG1	0.3146	0.3613	-0.0759	0.7647
PGG2	0.3075	0.1962	-0.1183	0.8529
PGG3	0.2588	0.2890	-0.0446	0.8475
DG1	0.4740	0.0963	-0.0170	0.7658
DG2	0.3323	-0.0579	-0.0731	0.8809
DG3	0.4401	0.0760	-0.0161	0.8003
UG0	0.2411	-0.0106	0.3298	0.8329
UG1	0.1807	-0.0348	0.4179	0.7915
UG2	0.0426	0.0470	0.1818	0.9629
UG3	-0.1836	-0.0397	0.5989	0.6060
UG4	-0.1001	0.0346	0.5851	0.6465
DEG1	-0.5444	-0.0331	0.1586	0.6774
DEG2	-0.3936	0.0615	-0.0184	0.8410
DEG3	-0.5657	-0.0030	0.0805	0.6735
MDF	-0.0097	-0.1556	-0.0300	0.9748
MDB	0.0608	-0.0636	-0.0658	0.9879
BEL1	-0.0397	0.5835	-0.0178	0.6576
BEL2	0.0324	0.5584	0.0349	0.6859
BEL3	0.0555	0.5565	-0.0153	0.6870

Supplementary Table 3: Factor loadings.

The table reports the factor loadings for the 3 retained factors in the first 3 columns, and the uniqueness coefficient for each task in the fourth column. Loadings with weights above 0.1 appear in blue, while those with weights below -0.1 appear in red.

Deception Game

In the DEG, preschoolers in the fast condition are less likely to deceive than those in the slow condition, although this effect diminishes with age. Older children are generally less likely to deceive than younger ones, and deception increases in later rounds.

Moral Dilemmas

Children are more likely to act in the Bystander dilemma than in the Footbridge dilemma. Older children are more likely to act in both types of dilemmas. Finally, we find no evidence that condition is associated with children's choices.

A.3 Non-Linear Age Regressions

This section replicates the earlier analysis, but instead of using age as a continuous control, participants are grouped into 4 balanced age categories: 3–4, 5–6, 7–8, and 9–10 years. The insights from these regressions align with those of the main analysis. However, these robustness checks highlight that the association with age is not linear. Rather, it appears to undergo developmental changes at specific ages. These results are reported in Supplementary Table 6, and Supplementary Figure 1. These conclusions are further corroborated by local polynomial regressions in Supplementary Figure 2.

The linear regressions reveal several key insights. In the context of Prosociality, children in the fast condition in the 3–4 age group showed higher prosocial tendencies

Factor	Prosociality	Social Optimism	Acquiescence
Constant	−0.715	−0.153	1.043
	(0.192)	(0.198)	(0.158)
Fast	0.997***	0.215	−0.535*
	(0.248)	(0.257)	(0.222)
	[<0.001]	[0.169]	[0.017]
Age	0.086***	0.020	−0.144***
	(0.024)	(0.025)	(0.022)
	[<0.001]	[0.171]	[<0.001]
Age × Fast	−0.113***	−0.027	0.069*
	(0.032)	(0.032)	(0.030)
	[<0.001]	[0.169]	[0.020]
R-squared	0.044	0.002	0.111
Observations	522	522	522

Supplementary Table 4: Linear regressions by factor.

Heteroskedasticity robust standard errors appear in parentheses ($df = 518$). Sharpened FDR q-values appear in square brackets. Significance stars are based on sharpened FDR q-values, with * denoting $q < .050$, ** denoting $q < .010$, *** denoting $q < .001$; no stars are reported for constants as they reflect factor normalization rather than substantive hypotheses.

compared to those in the slow condition, $b = 0.51$, $t(514) = 3.25$, $q = 0.004$, 95% CI = [0.20, 0.83]. Moreover, the difference between fast and slow decision modes is significantly larger among 5–6-year-olds than among 9–10-year-olds, $b = 0.41$, $t(514) = 2.17$, $q = 0.038$, 95% CI = [0.04, 0.79], highlighting that the effect of decision mode on Prosociality undergoes meaningful shifts across development. Acquiescence declines markedly at ages 9–10 in both conditions, $b = -0.88$, $t(514) = -6.57$, $q < 0.001$, 95% CI = [−1.14, −0.62], though the decline is attenuated under time pressure, $b = 0.42$, $t(514) = 2.26$, $q = 0.032$, 95% CI = [0.05, 0.79], with similar patterns emerging at ages 7–8. These insights highlight that development is often associated with non-linear shifts in factors, as seen by the changes in coefficients across age groups.

Task	PGG	DG	UG	DEG	MD
Constant	0.718	1.921	1.200	0.726	0.608
	(0.294)	(0.246)	(0.056)	(0.085)	(0.076)
Fast	0.537*	0.263	−0.134*	−0.625***	0.040
	(0.287)	(0.297)	(0.074)	(0.107)	(0.102)
	[0.048]	[0.166]	[0.049]	[<0.001]	[0.264]
Age	0.105***	0.030	−0.038***	−0.038***	0.028**
	(0.029)	(0.030)	(0.007)	(0.011)	(0.010)
	[<0.001]	[0.152]	[<0.001]	[<0.001]	[0.006]
Age × Fast	−0.066*	−0.024	0.017	0.071***	−0.004
	(0.036)	(0.036)	(0.010)	(0.014)	(0.013)
	[0.048]	[0.210]	[0.056]	[<0.001]	[0.297]
Round 2	0.328***	0.297***		0.069*	
	(0.066)	(0.065)		(0.027)	
	[<0.001]	[<0.001]		[0.011]	
Round 3	−0.034	0.229***		0.107***	
	(0.063)	(0.059)		(0.020)	
	[0.242]	[<0.001]		[<0.001]	
Beliefs	0.396***				
	(0.076)				
	[<0.001]				
Offer 0			−0.398***		
			(0.024)		
			[<0.001]		
Offer 1			−0.232***		
			(0.022)		
			[<0.001]		
Offer 3			−0.170***		
			(0.021)		
			[<0.001]		
Offer 4			−0.175***		
			(0.021)		
			[<0.001]		
Footbridge					−0.188***
					(0.024)
					[<0.001]
R-squared	0.034	0.013	0.107	0.049	0.061
Observations	1572	1575	2625	1575	1047

Supplementary Table 5: Linear regression by task.

Heteroskedasticity robust standard errors clustered by participant appear in parentheses. Degrees of freedom: PGG $df = 523$; DG $df = 524$; UG $df = 524$; DEG $df = 524$; MD $df = 523$. Sharpened FDR q-values appear in square brackets. Significance stars are based on sharpened FDR q-values, with * denoting $q < .050$, ** denoting $q < .010$, *** denoting $q < .001$; no stars are reported for constants as they capture baseline levels rather than the treatment effects or developmental patterns of interest.

Factor	Prosociality	Social Optimism	Acquiescence
Constant	−0.186	−0.024	0.453
	(0.130)	(0.154)	(0.097)
Fast	0.512**	0.067	−0.297*
	(0.163)	(0.190)	(0.137)
	[0.004]	[0.500]	[0.037]
Age 5-6	−0.086	−0.104	−0.134
	(0.167)	(0.180)	(0.125)
	[0.443]	[0.428]	[0.260]
Age 7-8	−0.099	0.080	−0.672***
	(0.163)	(0.182)	(0.129)
	[0.428]	[0.464]	[<0.001]
Age 9-10	0.514**	0.081	−0.881***
	(0.158)	(0.173)	(0.134)
	[0.003]	[0.460]	[<0.001]
Age 5-6 × Fast	−0.274	0.093	0.115
	(0.215)	(0.230)	(0.184)
	[0.200]	[0.477]	[0.426]
Age 7-8 × Fast	−0.264	−0.154	0.469*
	(0.211)	(0.228)	(0.180)
	[0.200]	[0.423]	[0.016]
Age 9-10 × Fast	−0.686**	−0.125	0.421*
	(0.208)	(0.220)	(0.187)
	[0.003]	[0.428]	[0.032]
R-squared	0.078	0.006	0.138
Observations	522	522	522

Supplementary Table 6: Factor regressions with age groups.

Heteroskedasticity robust standard errors appear in parentheses ($df = 514$). Sharpened FDR q-values appear in square brackets. Significance stars are based on sharpened FDR q-values, with * denoting $q < .050$, ** denoting $q < .010$, *** denoting $q < .001$; no stars are reported for constants as they reflect factor normalization rather than substantive hypotheses.

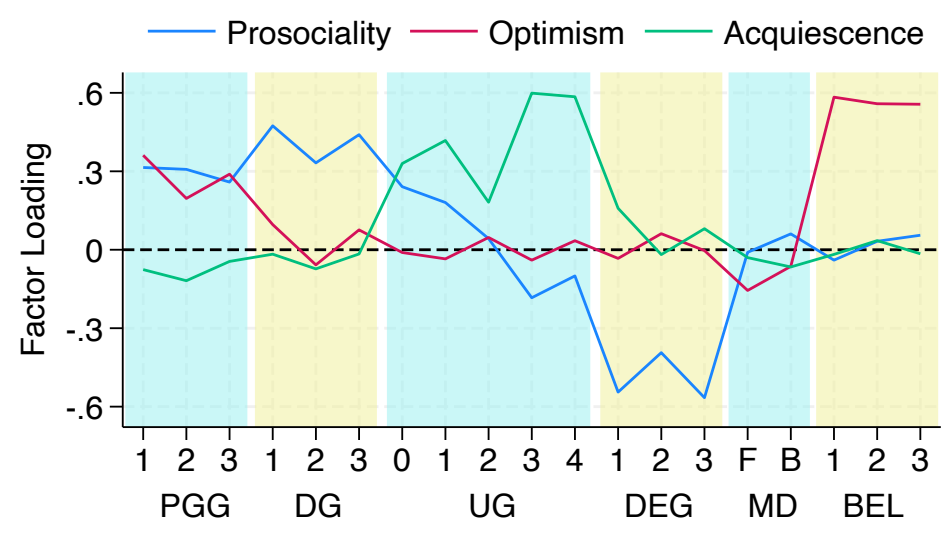

(a) Factor Loadings

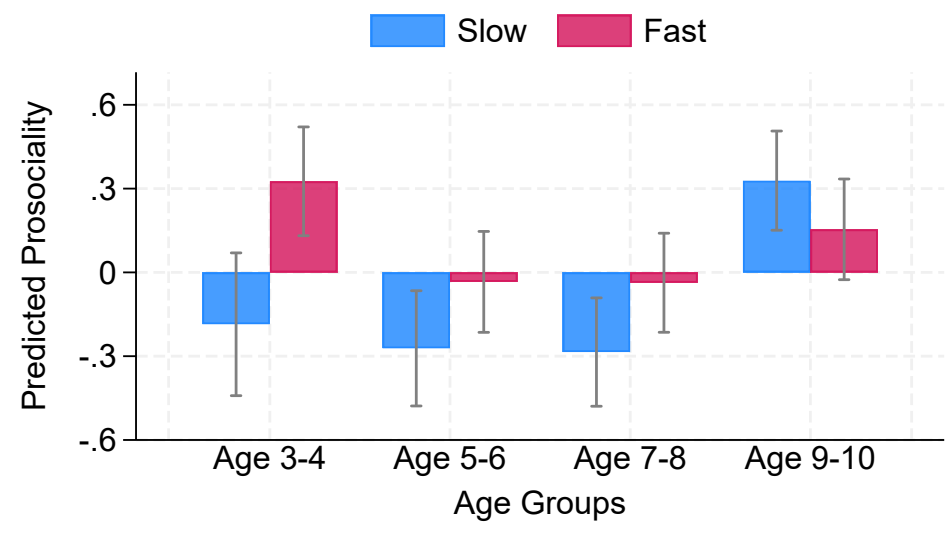

(b) Prosociality

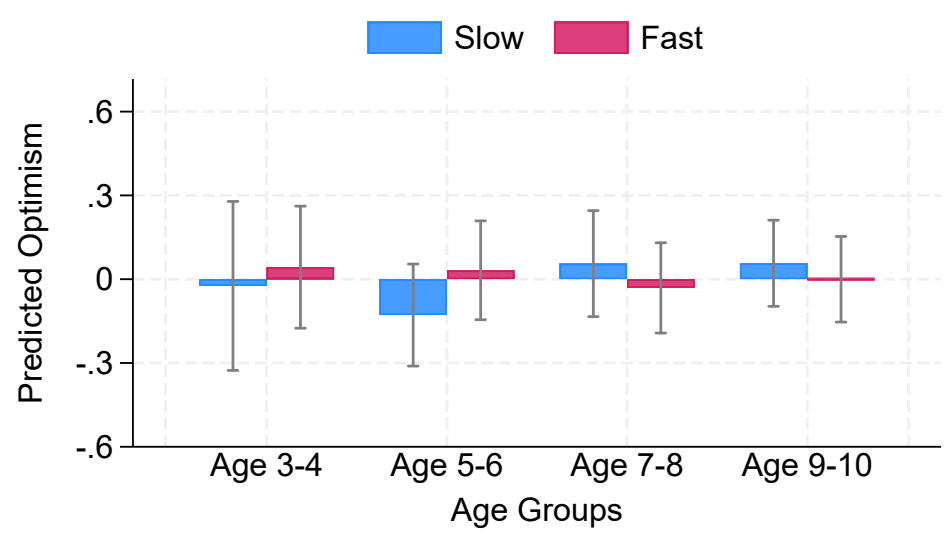

(c) Social Optimism

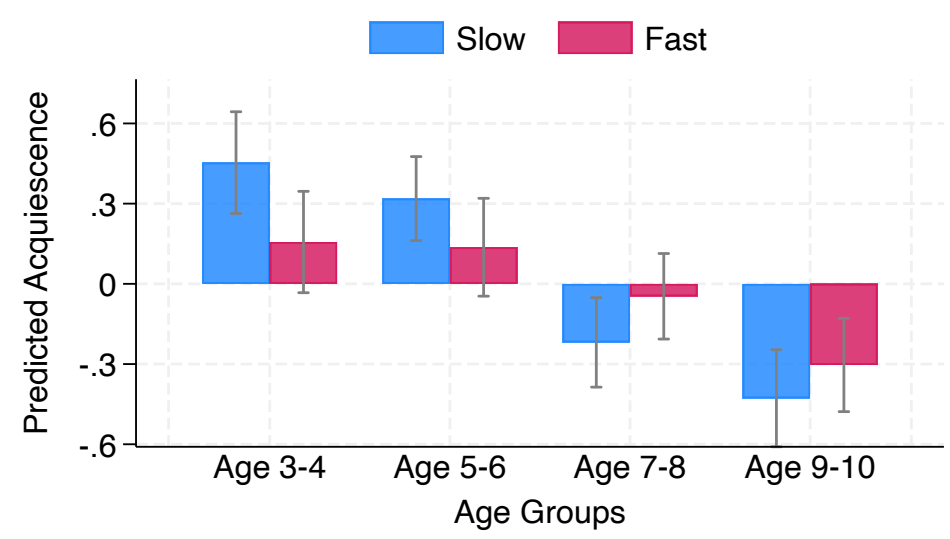

(d) Acquiescence

Supplementary Figure 1: Factor loadings and differences in factors by age group and decision mode.

Panel (a) depicts the factor loading for the 3 retained factors. Panels (b)–(d) depict the association with age groups estimated from linear regressions with factors as dependent variables and decision mode as control (n = 522). Bars represent mean values; error bars represent 95% CIs. All tests are two-sided and statistical significance is based on sharpened FDR q-values; full test statistics, degrees of freedom, confidence intervals, and q-values are reported in the main text and Supplementary Table 6.

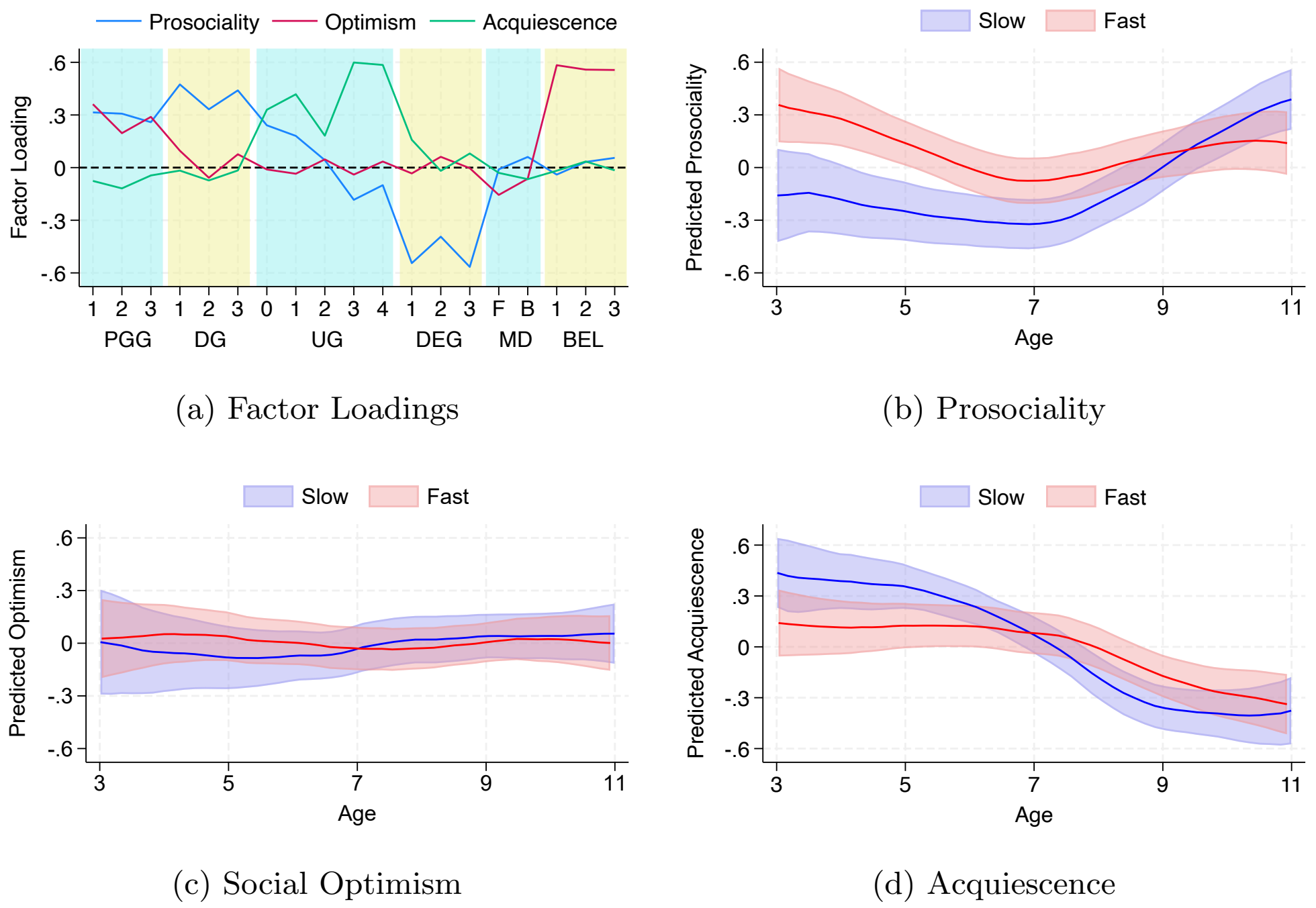

(a) Factor Loadings

(b) Prosociality

(c) Social Optimism

(d) Acquiescence

Supplementary Figure 2: Factor loadings and non-linear differences in factors by age and decision mode.

Panel (a) depicts the factor loading for the 3 retained factors. Panels (b)–(d) depict the association with age estimated from local polynomial regressions with factors as dependent variables and decision mode as control (n = 522). Lines represent local polynomial estimates; shaded bands represent 95% CIs. Note that local polynomial regressions are presented for descriptive purposes only; all formal tests are two-sided and based on the linear regressions reported in the main text and Supplementary Tables.

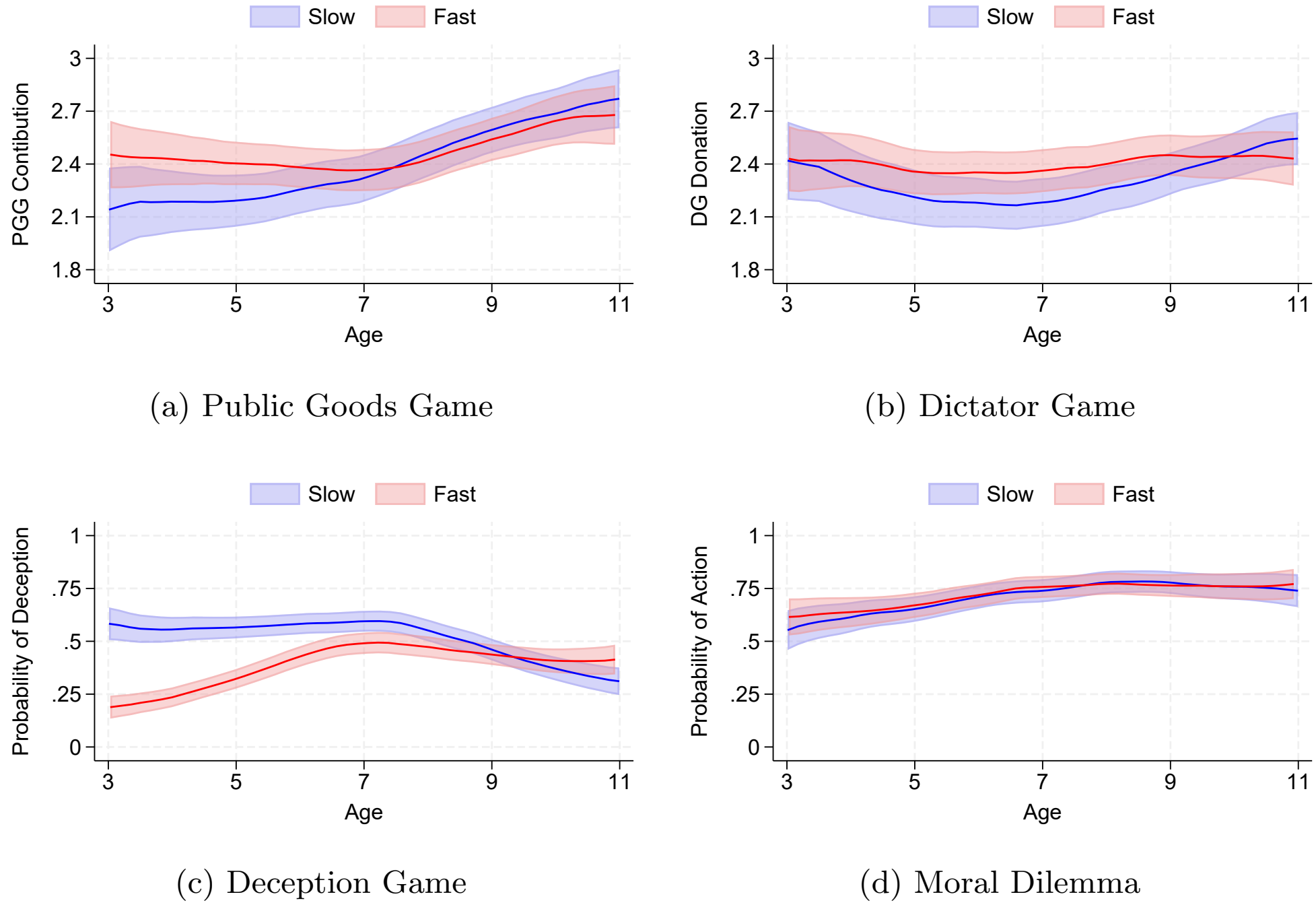

Supplementary Figure 3: Non-linear age and decision mode differences on PGG, DG, DEG and MD tasks.

Panels (a)–(d) depict the association with age estimated from local polynomial regressions with behaviour in PGG (n observations = 1,572), DG (n = 1,575), DEG (n = 1,575), and MD (n = 1,047) respectively as dependent variables and decision mode as control. Lines represent local polynomial estimates; shaded bands represent 95% CIs clustered by participant. Note that local polynomial regressions are presented for descriptive purposes only; all formal tests are two-sided and based on the linear regressions reported in the main text and Supplementary Tables.

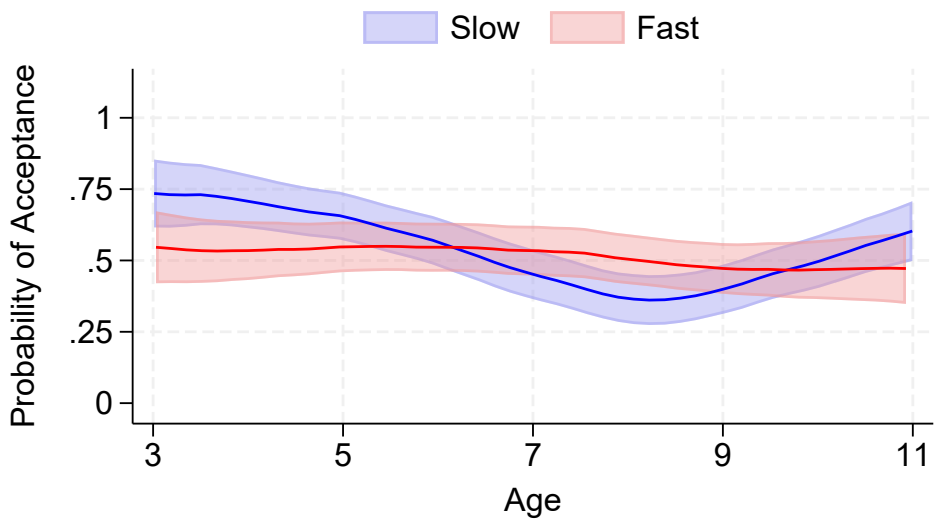

(a) Offer 0

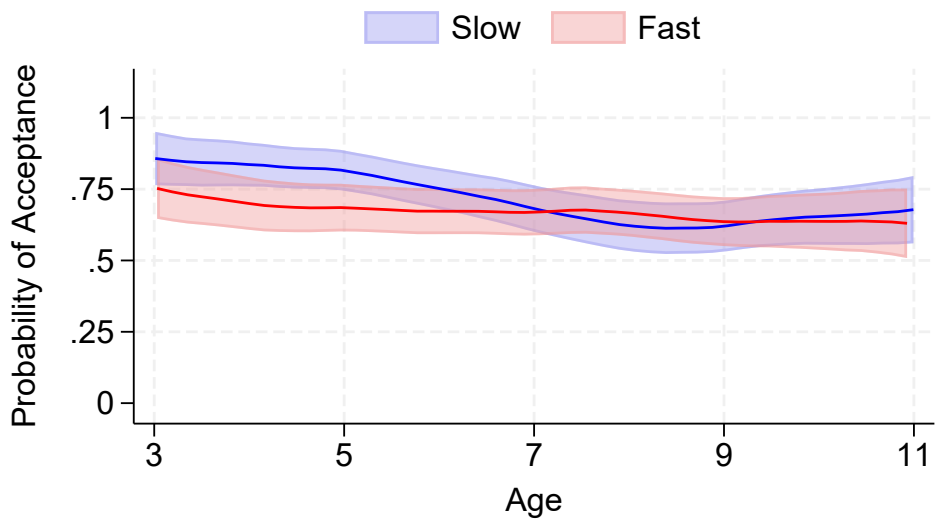

(b) Offer 1

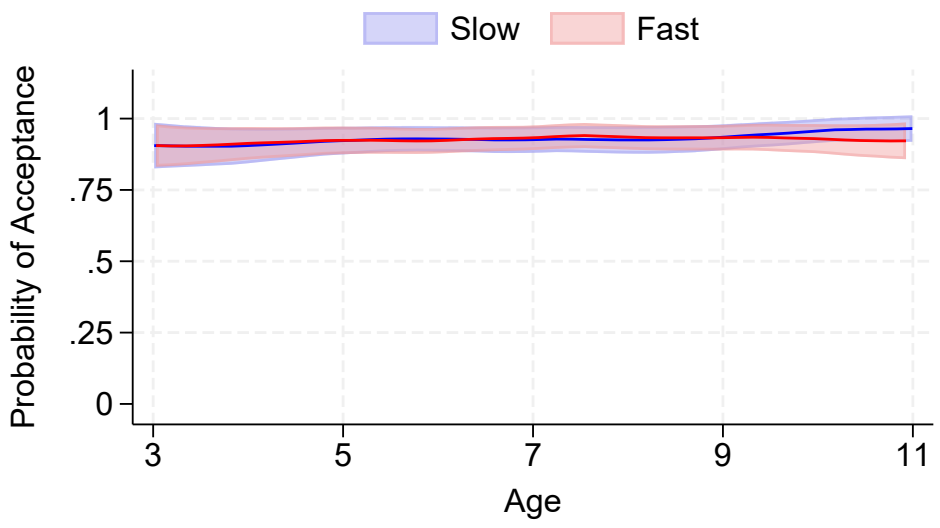

(c) Offer 2

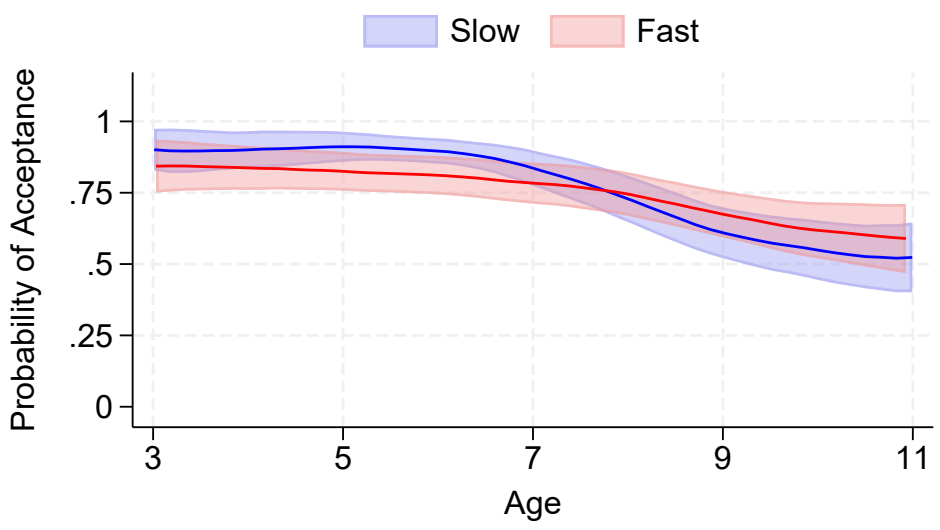

(d) Offer 3

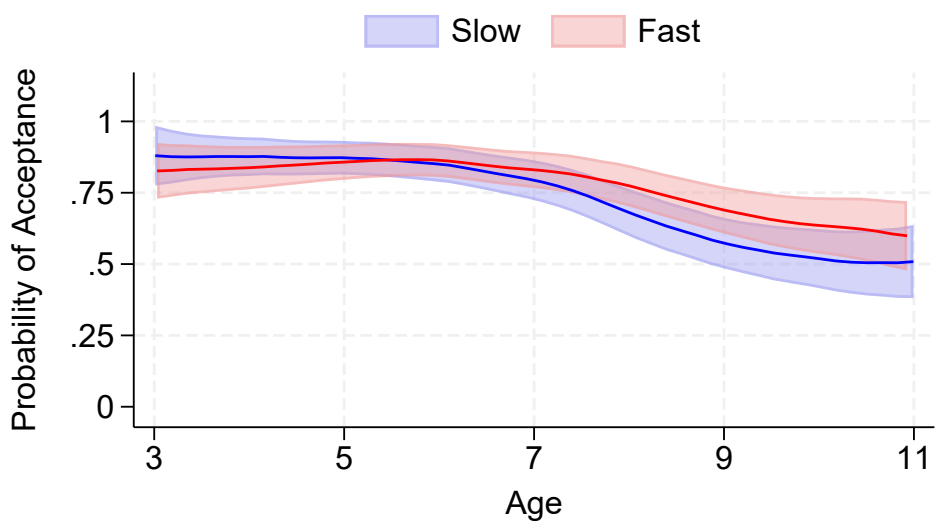

(e) Offer 4

Supplementary Figure 4: Non-linear age and decision mode differences on UG acceptance.

Panels (a)–(e) depict the association with age estimated from local polynomial regressions with behaviour in the 5 UG trials (4-0, 3-1, 2-2, 1-3, 0-4) respectively as dependent variables and decision mode as control (n = 2,625 observations, 524 participants). Lines represent local polynomial estimates; shaded bands represent 95% CIs clustered by participant. Note that local polynomial regressions are presented for descriptive purposes only; all formal tests are two-sided and based on the linear regressions reported in the main text and Supplementary Tables.

B Robustness Checks

B.1 Sample Inclusion Criteria

Supplementary Tables 7 and 8 replicate our main analysis, but include the participants who failed the comprehension checks. The results remain unaffected by this adjustment. In total, 10 participants failed 12 comprehension checks, with 2 failing more than one check.

Supplementary Tables 9 and 10 replicate our main analysis, excluding participants who did not comply with the fast condition. The results remain unaffected by this adjustment. In total, 66 participants were removed from the primary analysis due to non-compliance.

Supplementary Tables 11 and 12 replicate our main analysis, excluding participants aged 3–4 years. The results are robust to this sample restriction. In total, 117 participants aged 3–4 were removed from this analysis.

Loadings	Prosociality	Social Optimism	Acquiescence	Uniqueness
PGG1	0.3163	0.3570	-0.0616	0.7687
PGG2	0.3129	0.2003	-0.1167	0.8483
PGG3	0.2595	0.2934	-0.0370	0.8452
DG1	0.4705	0.1077	-0.0233	0.7664
DG2	0.3392	-0.0571	-0.0696	0.8768
DG3	0.4499	0.0761	-0.0126	0.7916
UG0	0.2150	-0.0071	0.3500	0.8312
UG1	0.1645	-0.0379	0.4333	0.7838
UG2	0.0391	0.0517	0.1798	0.9635
UG3	-0.1919	-0.0499	0.5922	0.6100
UG4	-0.1102	0.0306	0.5771	0.6539
DEG1	-0.5539	-0.0170	0.1549	0.6689
DEG2	-0.3872	0.0713	-0.0264	0.8443
DEG3	-0.5683	0.0109	0.0687	0.6722
MDF	-0.0151	-0.1361	-0.0384	0.9798
MDB	0.0705	-0.0443	-0.0721	0.9879
BEL1	-0.0469	0.5804	-0.0189	0.6605
BEL2	0.0251	0.5454	0.0140	0.7017
BEL3	0.0512	0.5552	-0.0155	0.6888

Supplementary Table 7: Factor loadings with participants failing comprehension.

The table reports the factor loadings for the 3 retained factors in the first 3 columns, and the uniqueness coefficient in the fourth column. Loadings with weights above 0.1 appear in blue, while those with weights below -0.1 appear in red.

Factor	Prosociality	Social Optimism	Acquiescence
Constant	−0.731	−0.167	1.026
	(0.185)	(0.182)	(0.148)
Fast	1.020***	0.177	−0.516*
	(0.240)	(0.246)	(0.213)
	[<0.001]	[0.251]	[0.017]
Age	0.089***	0.023	−0.143***
	(0.024)	(0.023)	(0.021)
	[<0.001]	[0.187]	[<0.001]
Age × Fast	−0.115***	−0.023	0.067*
	(0.031)	(0.031)	(0.029)
	[<0.001]	[0.251]	[0.021]
R-squared	0.048	0.002	0.115
Observations	532	532	532

Supplementary Table 8: Regressions with participants failing comprehension.

Heteroskedasticity robust standard errors appear in parentheses ($df = 528$). Sharpened FDR q-values appear in square brackets. Significance stars are based on sharpened FDR q-values, with * denoting $q < .050$, ** denoting $q < .010$, *** denoting $q < .001$; no stars are reported for constants as they reflect factor normalization rather than substantive hypotheses.

**Loadings**	**Prosociality**	**Social Optimism**	**Acquiescence**	**Uniqueness**
PGG1	0.3120	0.3520	-0.0638	0.7747
PGG2	0.3064	0.2139	-0.0950	0.8513
PGG3	0.2858	0.2799	-0.0280	0.8392
DG1	0.4514	0.0840	-0.0218	0.7887
DG2	0.3153	-0.0667	-0.0897	0.8881
DG3	0.4109	0.0769	-0.0266	0.8246
UG0	0.2263	-0.0289	0.3319	0.8378
UG1	0.2071	-0.0774	0.3993	0.7916
UG2	0.0335	0.0238	0.1240	0.9829
UG3	-0.1725	-0.0382	0.6092	0.5976
UG4	-0.1149	0.0400	0.6029	0.6217
DEG1	-0.5466	-0.0459	0.1733	0.6690
DEG2	-0.3683	0.0720	0.0101	0.8591
DEG3	-0.5735	-0.0108	0.0925	0.6624
MDF	-0.0722	-0.1659	-0.0057	0.9672
MDB	0.0526	-0.0681	-0.0446	0.9906
BEL1	-0.0561	0.6098	-0.0025	0.6250
BEL2	0.0606	0.5467	0.0275	0.6967
BEL3	0.0721	0.5593	-0.0393	0.6804

Supplementary Table 9: Factor loadings without non-compliants.

The table reports the factor loadings for the 3 retained factors in the first 3 columns, and the uniqueness coefficient in the fourth column. Loadings with weights above 0.1 appear in blue, while those with weights below -0.1 appear in red.

Factor	Prosociality	Social Optimism	Acquiescence
Constant	−0.682	−0.148	1.096
	(0.199)	(0.205)	(0.160)
Fast	0.723**	0.217	−0.581*
	(0.264)	(0.277)	(0.243)
	[0.009]	[0.274]	[0.019]
Age	0.087**	0.019	−0.151***
	(0.025)	(0.026)	(0.022)
	[0.001]	[0.277]	[<0.001]
Age × Fast	−0.082*	−0.026	0.077*
	(0.034)	(0.034)	(0.033)
	[0.019]	[0.274]	[0.021]
R-squared	0.036	0.002	0.123
Observations	457	457	457

Supplementary Table 10: Regressions without non-compliants.

Heteroskedasticity robust standard errors appear in parentheses ($df = 453$). Sharpened FDR q-values appear in square brackets. Significance stars are based on sharpened FDR q-values, with * denoting $q < .050$, ** denoting $q < .010$, *** denoting $q < .001$; no stars are reported for constants as they reflect factor normalization rather than substantive hypotheses.

**Loadings**	**Prosociality**	**Social Optimism**	**Acquiescence**	**Uniqueness**
PGG1	0.3811	0.2692	-0.0933	0.7736
PGG2	0.2782	0.0785	-0.1507	0.8937
PGG3	0.2607	0.1856	-0.0349	0.8964
DG1	0.4764	0.0256	-0.0275	0.7717
DG2	0.2746	-0.1337	-0.1302	0.8898
DG3	0.3966	0.0120	-0.0801	0.8362
UG0	0.3593	0.0088	0.1689	0.8423
UG1	0.2560	-0.0272	0.2546	0.8689
UG2	0.0682	0.0177	0.0509	0.9925
UG3	-0.1335	-0.0490	0.6180	0.5979
UG4	-0.0443	0.0013	0.6014	0.6363
DEG1	-0.4539	-0.0305	0.2875	0.7104
DEG2	-0.3117	0.0545	0.0319	0.8988
DEG3	-0.4986	-0.0397	0.2124	0.7047
MDF	-0.0279	-0.1190	-0.0318	0.9840
MDB	0.0485	0.0053	-0.0408	0.9960
BEL1	-0.0086	0.6018	-0.0042	0.6377
BEL2	0.0538	0.5244	-0.0338	0.7210
BEL3	0.0397	0.5790	-0.0308	0.6622

Supplementary Table 11: Factor loadings excluding 3–4 year olds.

The table reports the factor loadings for the 3 retained factors in the first 3 columns, and the uniqueness coefficient in the fourth column. Loadings with weights above 0.1 appear in blue, while those with weights below -0.1 appear in red. Sample restricted to participants aged 5 years and older.

	Prosociality	Social Optimism	Acquiescence
Constant	−1.084	−0.272	1.308
	(0.278)	(0.253)	(0.238)
Fast	0.937*	0.340	−0.491
	(0.367)	(0.357)	(0.346)
	[0.018]	[0.301]	[0.162]
Age	0.130***	0.034	−0.166***
	(0.033)	(0.030)	(0.030)
	[<0.001]	[0.242]	[<0.001]
Age × Fast	−0.104*	−0.042	0.064
	(0.044)	(0.043)	(0.043)
	[0.027]	[0.297]	[0.146]
R-squared	0.047	0.003	0.104
Observations	405	405	405

Supplementary Table 12: Regressions excluding 3–4 year olds.

Heteroskedasticity robust standard errors appear in parentheses ($df = 401$). Sharpened FDR q-values appear in square brackets. Significance stars are based on sharpened FDR q-values, with * denoting $q < .050$, ** denoting $q < .010$, *** denoting $q < .001$; no stars are reported for constants as they reflect factor normalization rather than substantive hypotheses. Sample restricted to participants aged 5 years and older.

B.2 Task Selection Criteria

Supplementary Tables 13 and 14 replicate our main analysis, excluding tasks with uniqueness coefficients exceeding 0.9 from the main factor analysis. In total, 3 trials were removed from the analysis due to high uniqueness values (UG2, MDF, and MDB). The results are robust to this adjustment.

Supplementary Tables 15 and 16 replicate our main analysis, excluding all Public Goods Game trials and their associated belief elicitations. In total, 6 trials were removed from this analysis (PGG1, PGG2, PGG3, BEL1, BEL2, BEL3). The results are robust to this adjustment, confirming that our findings are not driven by the inclusion of the PGG task. Note that this exclusion necessarily eliminates the Social Optimism factor, which was primarily associated with the belief elicitation trials.

Loadings	Prosociality	Social Optimism	Acquiescence	Uniqueness
PGG1	0.3175	0.3814	-0.0503	0.7512
PGG2	0.3033	0.2075	-0.1051	0.8539
PGG3	0.2696	0.2866	-0.0225	0.8447
DG1	0.4748	0.1031	-0.0184	0.7636
DG2	0.3375	-0.0560	-0.0730	0.8776
DG3	0.4412	0.0804	-0.0058	0.7988
UG0	0.2377	-0.0102	0.3370	0.8298
UG1	0.1781	-0.0407	0.4074	0.8007
UG3	-0.1799	-0.0439	0.6006	0.6050
UG4	-0.0945	0.0280	0.5834	0.6499
DEG1	-0.5420	-0.0220	0.1751	0.6750
DEG2	-0.3999	0.0624	-0.0236	0.8356
DEG3	-0.5668	0.0023	0.0910	0.6705
BEL1	-0.0381	0.5822	-0.0075	0.6596
BEL2	0.0177	0.5460	0.0103	0.7014
BEL3	0.0387	0.5514	-0.0271	0.6937

Supplementary Table 13: Factor loadings excluding high uniqueness tasks.

The table reports the factor loadings for the 3 retained factors in the first 3 columns, and the uniqueness coefficient in the fourth column. Loadings with weights above 0.1 appear in blue, while those with weights below -0.1 appear in red. Tasks with uniqueness exceeding 0.9 (UG2, MDF, MDB) are excluded from this analysis.

Factor	Prosociality	Social Optimism	Acquiescence
Constant	−0.681	−0.219	1.055
	(0.191)	(0.197)	(0.150)
Fast	0.996***	0.241	−0.570*
	(0.247)	(0.253)	(0.212)
	[<0.001]	[0.301]	[0.013]
Age	0.082**	0.029	−0.146***
	(0.024)	(0.024)	(0.021)
	[0.002]	[0.216]	[<0.001]
Age × Fast	−0.113**	−0.030	0.074*
	(0.032)	(0.032)	(0.029)
	[0.002]	[0.302]	[0.018]
R-squared	0.042	0.004	0.113
Observations	524	524	524

Supplementary Table 14: Regressions excluding high uniqueness tasks.

Heteroskedasticity robust standard errors appear in parentheses ($df = 520$). Sharpened FDR q-values appear in square brackets. Significance stars are based on sharpened FDR q-values, with * denoting $q < .050$, ** denoting $q < .010$, *** denoting $q < .001$; no stars are reported for constants as they reflect factor normalization rather than substantive hypotheses.

**Loadings**	**Prosociality**	**Acquiescence**	**Uniqueness**
DG1	0.4597	-0.0100	0.7885
DG2	0.3210	-0.0657	0.8927
DG3	0.4440	-0.0107	0.8028
UG0	0.2073	0.3348	0.8449
UG1	0.1870	0.4107	0.7964
UG2	0.0645	0.1694	0.9672
UG3	-0.1939	0.5975	0.6054
UG4	-0.0984	0.5738	0.6610
DEG1	-0.5711	0.1578	0.6490
DEG2	-0.3913	-0.0231	0.8464
DEG3	-0.5869	0.0800	0.6492
MDF	-0.0336	-0.0197	0.9985
MDB	0.0669	-0.0614	0.9917

Supplementary Table 15: Factor loadings excluding PGG and belief tasks.

The table reports the factor loadings for the 2 retained factors in the first 2 columns, and the uniqueness coefficient in the third column. Loadings with weights above 0.1 appear in blue, while those with weights below -0.1 appear in red. All PGG trials (PGG1-3) and belief elicitation trials (BEL1-3) are excluded, eliminating the Social Optimism factor.

Factor	Prosociality	Acquiescence
Constant	−0.624	0.990
	(0.191)	(0.155)
Fast	1.002***	−0.521*
	(0.242)	(0.218)
	[<0.001]	[0.026]
Age	0.073**	−0.137***
	(0.024)	(0.021)
	[0.006]	[<0.001]
Age × Fast	−0.113**	0.068*
	(0.032)	(0.030)
	[0.001]	[0.030]
R-squared	0.042	0.102
Observations	523	523

Supplementary Table 16: Regressions excluding PGG and belief tasks.

Heteroskedasticity robust standard errors appear in parentheses ($df = 519$). Sharpened FDR q-values appear in square brackets. Significance stars are based on sharpened FDR q-values, with * denoting $q < .050$, ** denoting $q < .010$, *** denoting $q < .001$; no stars are reported for constants as they reflect factor normalization rather than substantive hypotheses.

B.3 Time Pressure and Random Choice

Supplementary Table 17 examines whether time pressure moves younger children's responses toward random choice. We focus on 3–4 year old participants and test whether choices differ between time pressure and time delay conditions. To avoid distributional assumptions on the residuals, we conduct exact two-sided Wilcoxon rank-sum tests task by task. We find no systematic tendency for time pressure to move participants towards random choice. In 5 of the 9 tasks, behaviour in the fast condition is farther from random choice than behaviour in the slow condition. In the DEG task, choices differ significantly between conditions, with behaviour in the fast condition deviating more from random choice. In UG0, choices also differ significantly, with behaviour in the fast condition closer to random choice, but this does not extend to the other UG tasks (or any other task).

**Task**	**Mean Choice Time Pressure**	**Mean Choice Time Delay**	**Random Choice**	**z-statistic**	**Exact p-value**
PGG	2.44	2.17	2.00	1.58	0.112
DG	2.43	2.36	2.00	0.23	0.819
UG0	0.54	0.75	0.50	-2.37	0.027*
UG1	0.73	0.86	0.50	-1.69	0.139
UG2	0.92	0.91	0.50	0.19	1.000
UG3	0.86	0.91	0.50	-0.90	0.538
UG4	0.84	0.89	0.50	-0.82	0.583
DEG	0.21	0.60	0.50	4.07	0.000***
MD	0.61	0.58	0.50	0.51	0.707

Supplementary Table 17: Testing whether fast condition moves 3–4 year olds toward random choice.

The table reports mean choices for 3–4 year old participants in fast and slow conditions, the expected value under random choice, and two-sided exact Wilcoxon rank-sum tests for whether time pressure and time delay choices differ. Positive z-statistics indicate time pressure choices are farther from random choice than time delay choices, and negative that they are closer. All tests are two-sided. Significance is indicated with stars, with * denoting $p < .050$, ** denoting $p < .010$, *** denoting $p < .001$.

B.4 Estimating Factors by Age

The following linear regressions use the factors estimated by splitting the dataset, where factors are estimated using different weights above and below the median age in the sample. This robustness check demonstrates that the composition of 2 out of the 3 factors is unaffected by age. However, Acquiescence is no longer significant as a factor among older participants, and is consequently dropped from the analysis.

As was the case when factors were estimated on the full panel, linear regressions show that condition (fast, slow), age, and their interaction impact on Prosociality, but not on Social Optimism.

Factor	Prosociality	Social Optimism
Constant	−0.760***	−0.008
	(0.194)	(0.199)
Fast	1.254***	0.033
	(0.253)	(0.252)
Age	0.091***	0.000
	(0.025)	(0.026)
Age × Fast	−0.145***	−0.003
	(0.033)	(0.033)
R-squared	0.055	0.001
Observations	522	522

Supplementary Table 18: Split sample factors.

Heteroskedasticity robust standard errors appear in parentheses (df = 518). Significance is based on unadjusted p-values, with * denoting $p < .050$, ** denoting $p < .010$, *** denoting $p < .001$. This analysis serves as a descriptive validation of the factor structure and was not included in the sharpened FDR correction as it does not constitute a test of the treatment or developmental hypotheses of interest.

C Additional Scripts For Each Task

C.1 The PGG Task (Explanatory Movie-S1)

To explain the PGG to the children, the experimenter first showed them a short movie (Movie S1), which we detail here. The movie shows a male child actor sitting at a table. The child actor is on the left side of the scene. In front of him is an orange bowl (the common pool), with a female adult actor positioned behind the table, on the child's left, facing the viewer. She explains that the child on her right will play with 3 other players, each having 4 candies. The candies in front of the child actor are his own, and he decides what to do with them. The candies are taped together in pairs, totaling 4 in front of the child actor, between him and the bowl. The 3 partners are not real children but small closed boxes with images of children's faces (7 x 7 cm drawings) attached to them.

Facing the viewer, the adult actor says, "Hello and welcome! Today I'll explain the rules of a fun game. Listen carefully. Look! He is a child just like you." The child actor turns to the camera and says, "Hi! I'm Joseph." The adult continues, "Today he will play this game. Look, he has 4 candies." She picks up the candies and adds, "They are his own, and he can decide what to do with them." She then places them back and explains, "But in this game, you don't play alone. There are 3 other children... this child here, this child here, and this child here." The adult points to the 3 boxes, saying, "Each one of them, like Joseph, has 4 candies, and each decided how many to put in their box."

The adult actor says, "Now, let me explain how this works! Joseph has 4 candies and can decide to put 2 in the common bowl and keep 2 for himself." The child actor puts 2 candies in the bowl and keeps 2. The adult continues, "Or, he can put all 4 in the common bowl and keep none." The child actor places all the candies in the bowl. The adult then adds, "Or he can keep all 4 for himself, putting none in the bowl." The child actor keeps all 4 candies.

The adult actor looks at the camera, "Let's see an example so you understand. Joseph has 4 candies and can decide how to use them. Let's look at what Joseph decides to do." The child actor places 2 candies in the bowl and keeps 2. The adult comments, "Joseph has decided to put 2 candies in the common bowl and keep 2 for himself." The child actor brings the 2 candies closer. The adult continues, "The other 3 children already decided how many candies to put in the bowl, but we don't know yet how many. Let's find out." The adult opens each box, revealing the candies inside, placing them in front of the corresponding images, saying, "This child put 2 candies, this one put 4, and this one put none." She adds, "Now, I'll take them and put them all in the common bowl."

The adult grabs the bowl and shows it to the camera, "Do you know what happens when the candies are put in the common bowl? They double in number!" She then demonstrates, lining up the candies in front of her and adding an equal number from a small bag placed just outside the scene, helping the viewer understand that the total quantity is the same. She continues, "Now I'll put them all back in the bowl. And I'll distribute them equally among all the players, so everyone gets the same number of candies." The adult places 4 candies in front of each player, saying, "4 candies for

Joseph, 4 for this child, 4 for this child, and 4 for this child." Lastly, the adult engages the viewer: "Did you see what happened? Joseph had 4 candies, now he has 6, and he can take them home. But how many candies he gets depends on how many each child put in the bowl. Are you ready to play now?"

C.2 Additional Scripts and Instructions

Public Goods Game

Before asking the child participant to make her decision on the first trial, the experimenter repeated the instructions one last time: "Here are your candies. These are yours, and you can decide what to do with them—whether to put 2, all, or none in the common bowl, and how many to keep for yourself. Remember, if you put them in the common bowl, they will mix with those that the other children decided to give. In fact, now you will play with 3 children who have already played the game. You see, they have already made their choices, but we don't know how many candies they put in their boxes. Their boxes are closed, and we still don't know what they decided to put in the common bowl."

Dictator Game

In the fast condition (assuming the DG was administered after another task, where the child was already introduced to the idea of responding within 10 seconds), the experimenter prompted the child by saying, "(As in the previous game,) in this game too, you have to decide right away what to do. You only have 10 seconds to decide how many candies to give to this child, so you have to be really quick! Are you ready to go?" In the slow condition, the experimenter instead said, "(As in the previous game,) in this game too, you have to think very carefully before deciding how many candies to give to this child. You can't choose right away. Before deciding, you'll have to wait at least 10 seconds and think very, very carefully! After 10 seconds, you can take all the time you need to decide. I'm in no hurry, think carefully about what to do!"

Ultimatum Game

In the fast condition (assuming the first trial was 2-2, and UG was not the first task), to prompt the child's response, the experimenter said, "(As in the previous game,) in this game too, you have to decide right away whether to say yes or no. Listen, this child suggested keeping 2 candies for herself and giving you 2 candies. Are you okay with that? Tell me yes or no. You only have 10 seconds to decide, so you have to be really quick! Go!" In the slow condition, the experimenter said, "(As in the previous game,) in this game too, you need to think very carefully before deciding whether to say yes or no. You can't choose right away. Before deciding, you'll have to wait at least 10 seconds and think very, very carefully! After 10 seconds, you can take as long as you need to decide. I'm in no hurry, think carefully about how to respond!"

Deception Game

While giving the instructions, the experimenter also provided an example of how the game worked: "I'll show you how this game works. Look, to find out if I'm yellow or green, I'll draw a card. Oh, I'm yellow! See, the card is yellow! This means I am truly yellow, and this child here is green. Look, now I say "yellow!," and see, because I told the truth and said the true color of the card I drew, I receive 1 candy and she takes 3. Now, look what happens if I say "green!" Ah, it's not true that I'm green, but I can say green if I want, and if I say the false color, I take 3 candies and she only takes 1."

Next, participants were instructed on how to respond. In the fast condition, the experimenter said, "(As in the previous game,) in this game too, you have to decide right away how to respond—whether to say yellow or green. The color I'm showing you is the color of your group. As soon as I show it to you, I'll ask you to tell me the color of your group. You can say the true color and receive 1 candy, giving 3 to this child, or you can say the false color and receive 3 candies, giving 1 to this child. It's up to you! Remember, you only have 10 seconds to decide, so you have to be really quick!" After drawing a card for the child, the experimenter asked, "In which group are you, the yellows or the greens?" In the slow condition, the experimenter said, "(As in the previous game,) in this game too, you need to think very carefully before telling me if you are in the yellows or the greens. You can't say the color right away. After I show you the card with the color of your group and ask you the question, you'll have to wait at least 10 seconds and think very, very carefully! After 10 seconds, you can take all the time you want to decide. I'm in no hurry, think carefully about how to respond!"

Moral Dilemmas

Before the first dilemma, the experimenter introduced the task: "In this game, I'll tell you 2 stories. On the screen, you will see a story with some cartoons. It's important that you listen carefully to what happens in the story because later you will have to decide how the story should end. You decide the ending!" The experimenter then asked, "Who will decide how the story ends?" Once the experimenter ensured the child understood that they had to decide, she proceeded with the first story.

For the Bystander Dilemma, the experimenter followed this script: "Mary/Tom is going for a walk. S/he is walking down a hill. [Animation 1: A ball appears at the top of the hill] S/he sees that a giant ball is rolling down the trail. [Animation 2: 5 children appear] The ball is rolling directly toward the 5 children. The children cannot escape because they are looking straight ahead and cannot hear or see the ball." Participants then received the first probe: "Do the children see the ball rolling down the hill?" After the probe, the experimenter continued: "[Animation 3: The ball rolls down a bit] The ball is rolling down the path and will hurt the 5 children. [Animation 4: The ball is deflected] Mary/Tom knows that if s/he pushes the fence across the trail, the ball will be pushed over to the other trail. [Animation 5: A lone child appears] But on this other trail, there is another child walking down the hill who cannot see or hear the ball coming." At this point, the second probe was presented: "Does this child see or hear the ball rolling down the hill?" After the probe, the experimenter continued: "If the ball is pushed sideways, this child will get hurt, but the other 5 will be safe."

Then, the first animation was shown again and the child received the test question: “What should Mary/Tom do? Push the ball over to the side or not push the ball?”

For the Footbridge Dilemma, the experimenter said: “Luisa/John is going for a walk. S/he is walking down a hill. [Animation 1: A ball appears at the top of the hill] S/he sees that a giant ball is rolling down the trail. [Animation 2: 5 children appear] The ball is rolling directly toward the 5 children. The children cannot escape because they are looking straight ahead and cannot hear or see the ball.” After receiving the same probe as in the previous dilemma, children were told: “[Animation 3: The ball rolls down a bit] The ball is rolling down the path and will hurt the 5 children. [Animation 4: Bridge and child with backpack appear] Luisa/John is on the bridge between the ball and the 5 children. There is also a child with a big backpack on the bridge. This child cannot hear or see the ball coming because s/he is looking straight ahead.” After the second probe, as in the previous dilemma, children were told: “Luisa/John knows that the only way to stop the ball is to block it with something heavy. [Animation 5: The child is pushed] Luisa/John knows that if s/he pushes the child with the big backpack down onto the trail, the child will get hurt, but the other 5 children will be safe.” The first animation was presented again, and children received the test question: “What should Luisa/John do? Push the child down onto the trail or not push the child?”

In the fast condition, right before the test question, the experimenter said, “(As in the previous game,) in this game too, you have to decide right away what Maria/Luisa should do. You only have 10 seconds to give your answer, so you have to be really quick!” In the slow condition, the experimenter said, “(As in the previous game,) in this game too, you need to think very carefully before deciding what Maria/Luisa should do. You can’t answer right away. Before giving me your answer, you’ll have to wait at least 10 seconds and think very, very carefully! After 10 seconds, you can take all the time you want to respond. I’m in no hurry, think carefully about how to respond!”

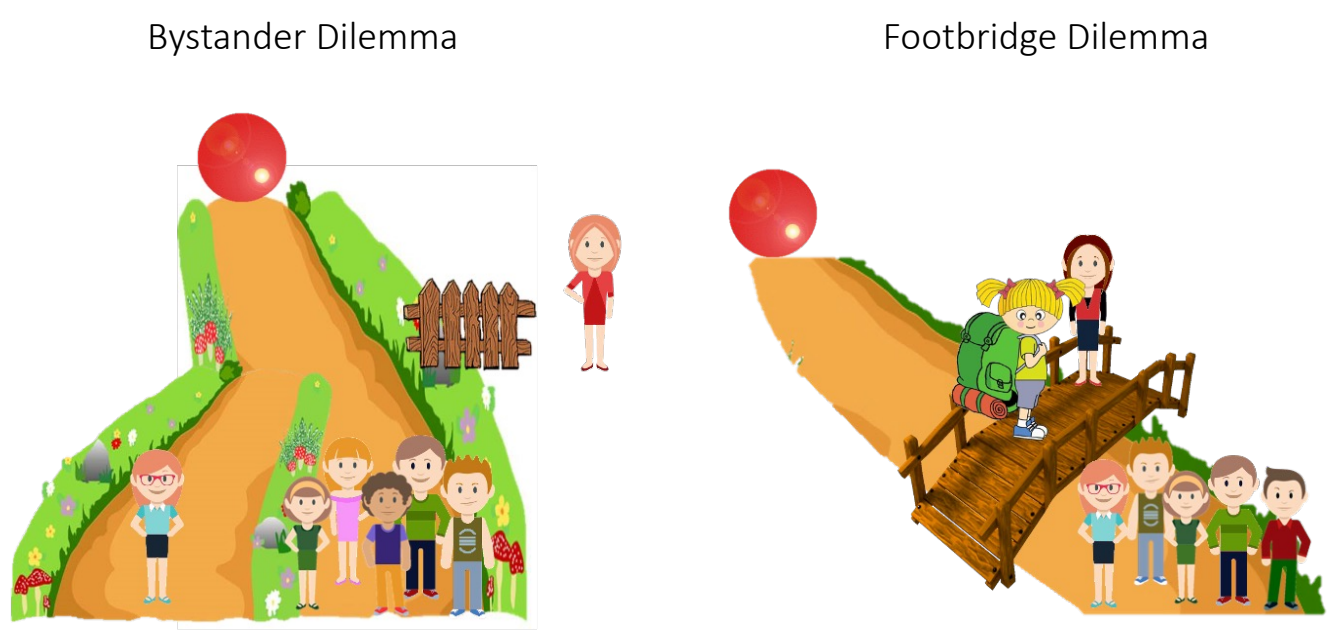

Supplementary Figure 5: Bystander and Footbridge Dilemmas stimuli.

The figure shows the first PowerPoint slide of the animation presented to children to illustrate the Bystander Dilemma (left) and the Footbridge Dilemma (right). In the figure, the female version of the stimuli is shown. A male version was used with male participants. Stimuli were originally designed by Dworazik et al. (2019).
