# Supplementary material for: Stable intuition and the rise of deliberative prosociality in childhood
Source: Nat Hum Behav. 2026 Jul 6;10(9):1686–97. doi: 10.1038/s41562-026-02487-4 (PMC13590409; doi:10.1038/s41562-026-02487-4)
Supplement: Supplementary file 2 [file 41562_2026_2487_MOESM2_ESM.pdf]

nature portfolio

Corresponding author(s): Francesco Margoni
Last updated by author(s): Feb 16, 2026

Reporting Summary

Nature Portfolio wishes to improve the reproducibility of the work that we publish. This form provides structure for consistency and transparency in reporting. For further information on Nature Portfolio policies, see our Editorial Policies and the Editorial Policy Checklist.

Statistics

For all statistical analyses, confirm that the following items are present in the figure legend, table legend, main text, or Methods section.

n/a	Confirmed	
☐	☒	The exact sample size (*n*) for each experimental group/condition, given as a discrete number and unit of measurement
☐	☒	A statement on whether measurements were taken from distinct samples or whether the same sample was measured repeatedly
☐	☒	The statistical test(s) used AND whether they are one- or two-sided<br>*Only common tests should be described solely by name; describe more complex techniques in the Methods section.*
☐	☒	A description of all covariates tested
☐	☒	A description of any assumptions or corrections, such as tests of normality and adjustment for multiple comparisons
☐	☒	A full description of the statistical parameters including central tendency (e.g. means) or other basic estimates (e.g. regression coefficient) AND variation (e.g. standard deviation) or associated estimates of uncertainty (e.g. confidence intervals)
☐	☒	For null hypothesis testing, the test statistic (e.g. *F*, *t*, *r*) with confidence intervals, effect sizes, degrees of freedom and *P* value noted<br>*Give P values as exact values whenever suitable.*
☐	☒	For Bayesian analysis, information on the choice of priors and Markov chain Monte Carlo settings
☒	☐	For hierarchical and complex designs, identification of the appropriate level for tests and full reporting of outcomes
☐	☒	Estimates of effect sizes (e.g. Cohen's *d*, Pearson's *r*), indicating how they were calculated

Our web collection on statistics for biologists contains articles on many of the points above.

Software and code

Policy information about availability of computer code

Data collection	No software was used.
Data analysis	All analyses were carried out using Stata 18 (StataCorp).

For manuscripts utilizing custom algorithms or software that are central to the research but not yet described in published literature, software must be made available to editors and reviewers. We strongly encourage code deposition in a community repository (e.g. GitHub). See the Nature Portfolio guidelines for submitting code & software for further information.

Data

Policy information about availability of data

All manuscripts must include a data availability statement. This statement should provide the following information, where applicable:

- Accession codes, unique identifiers, or web links for publicly available datasets
- A description of any restrictions on data availability
- For clinical datasets or third party data, please ensure that the statement adheres to our policy

The dataset generated and analysed during the current study is available in the Open Science Framework repository: https://osf.io/xpsqe/overview.

Research involving human participants, their data, or biological material

Policy information about studies with human participants or human data. See also policy information about sex, gender (identity/presentation), and sexual orientation and race, ethnicity and racism.

Reporting on sex and gender	We treated age as a main independent variable, both as a continuous measure and, in robustness checks, as a grouped variable. We report data from children, with gender information provided by their parents. The final sample was balanced by gender. However, gender was not theoretically relevant to the present study, as no gender-related hypotheses were formulated. Accordingly, gender was not included as an independent variable in the analyses.
Reporting on race, ethnicity, or other socially relevant groupings	We included a sample of Italian children recruited from preschools and primary schools in the Milano region. We did not collect data on race or ethnicity; the only available demographic information is that all participating children were native Italian speakers.
Population characteristics	See below.
Recruitment	Participants were recruited from kindergartens and primary schools in Milano (Italy) serving predominantly middle-income communities, and were tested with caregivers' written consent. Data collection took place between fall 2023 and spring 2024. Participation was voluntary, and the sample should therefore be considered a convenience sample. Potential sources of bias include school-level participation decisions, caregiver consent, and the regional and socioeconomic composition of the sample. These factors may limit generalizability to other populations but are unlikely to differentially affect the experimental manipulation.
Ethics oversight	The research was approved by the Ethical Committee of the University of Milano-Bicocca (protocol no. 0048825).

Note that full information on the approval of the study protocol must also be provided in the manuscript.

Field-specific reporting

Please select the one below that is the best fit for your research. If you are not sure, read the appropriate sections before making your selection.

☐ Life sciences ☒ Behavioural & social sciences ☐ Ecological, evolutionary & environmental sciences

For a reference copy of the document with all sections, see nature.com/documents/nr-reporting-summary-flat.pdf

Behavioural & social sciences study design

All studies must disclose on these points even when the disclosure is negative.

Study description	We conducted a quantitative experimental study by administering laboratory tasks to a sample of over 500 children. Rather than assuming that behavior is governed by five distinct, game-specific dimensions, we allowed the data to reveal whether underlying, common character traits emerged. To this end, we conducted a factor analysis to identify systematic behavioral patterns and assess within-subject variation across tasks.
Research sample	A total of 537 children (ages 3 to 10; 254 female) participated in the study, completing 5 tasks under either time pressure (respond within 10 seconds) or time delay (wait at least 10 seconds before responding). For each age group (3-year-olds, 4-year-olds, etc.), approximately 70 children were tested, with half under time pressure and half under time delay. An a-priori sample size calculation was conducted to determine the minimum sample size. Children aged 3–10 years were selected because this developmental period allows examination of early and middle childhood changes in intuitive and deliberative social decision making. Participants were recruited from kindergartens and primary schools in Milano, providing access to a broad community sample. The sample should be considered a convenience sample and is not intended to be representative of the broader population.
Sampling strategy	We employed a convenience sampling strategy, recruiting children from schools that agreed to participate in our project.
Data collection	Children completed five tasks administered either under time pressure or time delay. They were tested individually in a quiet room at their school. The tasks included the Public Goods Game (cooperation), the Dictator Game (altruism), the Ultimatum Game (aversion to inequality), the Deception Game (aversion to lying), and two Moral Dilemmas (utilitarian vs. deontological judgments). Responses were recorded manually by the experimenters using paper and pencil. Research assistants administering the tasks were not blind to the experimental condition or to the study's main hypotheses. No one was present during testing other than the participant and the researcher.
Timing	Data collection took place between fall 2023 and spring 2024.
Data exclusions	Compliance with our main manipulation (time pressure) was approximately 95%. As in previous studies employing time pressure, all participants were retained in the main analyses. In the Supplementary Materials, we report additional analyses excluding non-compliant participants, which overall confirm the main patterns of results. Additionally, data from 12 participants who failed the comprehension checks were excluded from the analyses. We show in the Supplementary Materials that including these participants does not alter the results.

Non-participation	There were no participant dropouts.
Randomization	Participants were randomly assigned to the experimental conditions.

Reporting for specific materials, systems and methods

We require information from authors about some types of materials, experimental systems and methods used in many studies. Here, indicate whether each material, system or method listed is relevant to your study. If you are not sure if a list item applies to your research, read the appropriate section before selecting a response.

Materials & experimental systems

n/a	Involved in the study
☒	☐ Antibodies
☒	☐ Eukaryotic cell lines
☒	☐ Palaeontology and archaeology
☒	☐ Animals and other organisms
☒	☐ Clinical data
☒	☐ Dual use research of concern
☒	☐ Plants

Methods

n/a	Involved in the study
☒	☐ ChIP-seq
☒	☐ Flow cytometry
☒	☐ MRI-based neuroimaging

Plants

Seed stocks	*Report on the source of all seed stocks or other plant material used. If applicable, state the seed stock centre and catalogue number. If plant specimens were collected from the field, describe the collection location, date and sampling procedures.*
Novel plant genotypes	*Describe the methods by which all novel plant genotypes were produced. This includes those generated by transgenic approaches, gene editing, chemical/radiation-based mutagenesis and hybridization. For transgenic lines, describe the transformation method, the number of independent lines analyzed and the generation upon which experiments were performed. For gene-edited lines, describe the editor used, the endogenous sequence targeted for editing, the targeting guide RNA sequence (if applicable) and how the editor was applied.*
Authentication	*Describe any authentication procedures for each seed stock used or novel genotype generated. Describe any experiments used to assess the effect of a mutation and, where applicable, how potential secondary effects (e.g. second site T-DNA insertions, mosiacism, off-target gene editing) were examined.*
