# Supplementary material for: Stable intuition and the rise of deliberative prosociality in childhood
Source: Nat Hum Behav. 2026 Jul 6;10(9):1686–97. doi: 10.1038/s41562-026-02487-4 (PMC13590409; doi:10.1038/s41562-026-02487-4)
Supplement: Supplementary file 3 [file 41562_2026_2487_MOESM3_ESM.pdf]

nature portfolio

Peer Review File

Stable Intuition and the Rise of Deliberative Prosociality in Childhood

Corresponding Author: Dr Francesco Margoni

Version 0:

Decision Letter:

18th August 2025

Dear Dr Margoni,

Thank you once again for your manuscript, entitled "The Rise of Deliberative Prosociality", and for your patience during the peer review process.

Your Article has now been evaluated by 3 referees. You will see from their comments copied below that, although they find your work of potential interest, they have raised quite substantial concerns. In light of these comments, we cannot accept the manuscript for publication, but would be interested in considering a revised version if you are willing and able to fully address reviewer and editorial concerns.

We hope you will find the referees' comments useful as you decide how to proceed. If you wish to submit a substantially revised manuscript, please bear in mind that we will be reluctant to approach the referees again in the absence of major revisions. We are committed to providing a fair and constructive peer-review process. Do not hesitate to contact us if there are specific requests from the reviewers that you believe are technically impossible or unlikely to yield a meaningful outcome.

Specifically, please prioritize the following:
1. All reviewers raised concerns about your theoretical framing, in particular, the predictions based on Social Heuristics Hypothesis (SHH) and the extent to which your design can distinguish between SHH and nativist accounts. As suggested by Reviewer #2, your manuscript can be substantially strengthened if you reframe the theoretical argument and shift the focus from adjudicating between the two theories to the developmental dynamics.
2. Another major concern raised by Reviewer #1 and Reviewer #2 is the validity/appropriateness of the experimental methods among the youngest children, in particular, task comprehension and the 10-sec time-pressure manipulation. In your revision, please address these concerns thoroughly.
3. Reviewer #2 also pointed out that the latent variables could be interpreted differently. Please respond to this concern by providing a more nuanced discussion of these latent factors (including alternative interpretations) as recommended by Reviewer #2.

Finally, your revised manuscript must comply fully with our editorial policies and formatting requirements. Failure to do so will result in your manuscript being returned to you, which will delay its consideration. To assist you in this process, I have attached a checklist that lists all of our requirements. If you have any questions about any of our policies or formatting, please don't hesitate to contact me.

If you wish to submit a suitably revised manuscript, we would hope to receive it within 4 months. I would be grateful if you could contact us as soon as possible if you foresee difficulties with meeting this target resubmission date.

With your revision, please:

• Include a “Response to the editors and reviewers” document detailing, point-by-point, how you addressed each editor and referee comment. If no action was taken to address a point, you must provide a compelling argument. When formatting this document, please respond to each reviewer comment individually, including the full text of the reviewer comment verbatim followed by your response to the individual point. This response will be used by the editors to evaluate your revision and sent back to the reviewers along with the revised manuscript.

• Highlight all changes made to your manuscript or provide us with a version that tracks changes.

Please use the link below to submit your revised manuscript and related files:

Link Redacted

Note: This URL links to your confidential home page and associated information about manuscripts you may have submitted, or that you are reviewing for us. If you wish to forward this email to co-authors, please delete the link to your homepage.

Thank you for the opportunity to review your work. Please do not hesitate to contact me if you have any questions or would like to discuss the required revisions further.

Sincerely,

[redacted]
Nature Human Behaviour

Reviewer expertise:

Reviewer #1: Developmental psychology, cooperative behaviors

Reviewer #2: Developmental Psychology, Social Cognition

Reviewer #3: Developmental psychology, cooperation, altruism

REVIEWER COMMENTS:

Reviewer #1 (Remarks to the Author):

This study examined the role of intuitive and deliberate decision processes on social decisions in 3- to 10-year-old children. To capture social decision-making, several tasks were included that assess cooperative behaviour and moral judgements. Factor analysis was employed to identify common dimensions of social behaviour (Prosociality, Social Optimism, and Acquiescence). Intuitive responses were more prosocial than deliberate for younger children, but this difference diminished with age. Social Optimism was unaffected by age and decision mode, whereas Acquiescence was unaffected by decision mode but declined with age. I see several strengths with this study: the authors have identified an important empirical question and used a rigorous methodological and analytic approach with a large (well-justified) sample size. My main questions centre on how this study can inform the predictions of the Social Heuristics Hypothesis and Nativist theories (which serve as the theoretical foundation), and the extent to which the youngest participants understood the more complex tasks, such as the public-goods game.

Theoretical framing

P. 3 In my reading of several SHH papers, there is no explicit developmental prediction, but there is an assumption that heuristics must be acquired through social experience, and so it makes sense that social heuristics would be acquired during early and middle childhood. I understand the logic, offered by the authors, that early social environments within the family may be more supportive of prosocial heuristics compared to the social environments children encounter when entering school, and so the social heuristics acquired may shift from prosocial to self-serving. At the same time, these claims about the type of social environment children are likely to encounter at various developmental stages are not self-evident and so should be made with supporting evidence.

An alternative possibility is that children find themselves in relatively stable social environments (in terms of supporting prosocial or self-serving heuristics) throughout the preschool-to-school-age years. This could lead to developmental stability in social intuitions across the age range studied here, in which case developmental continuity would be equally consistent with the Nativist and SHH accounts. If the acquired heuristics are prosocial, then the same developmental continuity predicted by the authors' in their framing of the nativist account may be observed, in which case it would not be possible to differentiate between the two accounts.

I think it might help to lay out specifically which pattern of evidence in the current study would align with SHH and which would align with a nativist account. A tricky thing about SHH here is that it is hard to imagine a pattern that could falsify it without a way to test alignment between children's social intuition and their social environment. Similarly, most contemporary nativist theories also allow for developmental change after early emergence and a role for social learning, so it is not immediately obvious how these perspectives can be adjudicated based on the pattern of findings in this study. Making explicit predictions about expected patterns would help.

Comprehension for youngest participants

One concern I have is with the level of task comprehension that the youngest participants are likely to have. This seems particularly important when decision-time manipulations are used, as there is a potential for time pressure to exacerbate comprehension problems and, in turn, drive condition effects in unintended ways.

I'll focus on the PGG, as this was the most complex of the resource-allocation tasks. The comprehension check verified that children understood that the candies they kept went to themselves and the ones they put in the common bowl went to everyone. But would the three-year-olds have understood the more complex nuances of the PGG? For example, even though the candies are doubled when put in the common bowl, it is better for me to just keep the candies and hope that others contribute if I want to self-maximize. Basically, do they understand that donations to the group are leveraged to provide a larger benefit to the group than keeping the candies can provide to themselves, but that these cooperative decisions are personally costly and the way to self-maximise is to keep everything for yourself?

My impression is that the comprehension check showed that participants understood the mechanics of who gets what candies (similar to what would be required for a DG), but it seems ambiguous whether they would understand these nuances of the PGG. Are there any additional checks that were not fully described in the manuscript? Or is there any precedent for using a PGG with children this young (I'm not aware of any and couldn't identify any in a quick search).

It may also help to explain how the evaluation was made that all tasks are age-appropriate for the youngest participants.

Minor points

P. 4 I realise there are significant word constraints for the manuscript, but it might be helpful to provide additional information about the development of intuitive cooperation in past literature. There are additional studies (not cited e.g Grossmann et al., 2020; Chajes et al., 2022, not exaustive) that have examined this question in the developmental literature, though most use decision-time correlations rather than manipulations. If the authors have decided to selectively cover only studies using decision-time manipulations, that could probably be stated very quickly as the literature is introduced.

P. 5 Were judgments about other players' contributions in the PGG made under the decision-time manipulation? If not, does it make sense to look for a condition effect for Social Optimism if the primary factor is not subject to a condition manipulation? I may be missing something here, so would appreciate clarification.

P. 10 It would be good to mention explicitly that the direction of the relation between PGG contributions and predictions about the contributions of others is not clear, just that there is an association. I think this point is alluded to with "and/or vice versa", but the conclusions are more directional. You could also cite Corbit et al., 2023, Dev Sci; they found a similar association, though only when decisions were made under time pressure.

P. 10 I believe SHH would be agnostic about whether intuition favours self-serving or prosocial behaviours in older children. This would depend on the social environment that the older children encounter, and it seems important to show that this environment supports self-serving heuristics in order to claim that SHH would predict this. I may have missed a paper that makes these sorts of predictions from SHH, but if this is based on about the authors' own assertion about children's social environments, then I believe these conclusions could be made more cautiously.

PP. 10–11 I find the claim that "deliberate prosociality rises with age" difficult to interpret because the study's two-condition design (pressure vs. delay) only allows relative rather than absolute inferences. Prior developmental work shows a general increase in prosocial behaviour across this age range, so the upward trend observed in the delay condition could simply mirror that baseline trajectory rather than reveal a unique effect of deliberation. Without a neutral-timing control, we cannot disentangle whether fast decisions elevate prosociality, slow decisions depress it, or both. Once prosociality rates converge across the two conditions at about eight years of age, the data indicate merely that the timing manipulation no longer has a significant effect on behavior (relative to each other, and we don't that what might be happening relative to baseline). In short, the current design more strongly supports conclusions about the relative contributions of intuitive versus deliberative processes, but the separate developmental trajectories for each process are not clear.

Reviewer #2 (Remarks to the Author):

This manuscript reports on an ambitious and important study investigating the developmental origins of intuitive and deliberative social decision-making. The authors use a large sample of children (N=537, ages 3-10) across a battery of five social tasks, employing a time-constraint manipulation to distinguish intuitive from deliberative responses. The use of factor analysis to identify latent traits of "Prosociality," "Social Optimism," and "Acquiescence" is a novel and valuable methodological contribution. The core finding—that intuitive prosociality is high and stable from a young age, while deliberative prosociality increases with age—is provocative and has the potential to significantly advance the field.
Despite these strengths, the manuscript requires major revision before it is suitable for publication. The primary concerns relate to (1) the theoretical framing of the research, (2) the conceptual interpretation of the latent factors derived from the analysis, and (3) the methodological validity of the core experimental manipulation and the generalizability of the findings.
Major Concerns

1. Theoretical Framing: Nativism vs. Social Heuristics Hypothesis (SHH)

The authors frame their study as a critical test between nativist accounts and the Social Heuristics Hypothesis (SHH), concluding that their findings support nativism while being "more difficult to reconcile" with the SHH. This framing relies on a narrow interpretation of the SHH. The SHH posits that intuitions are heuristics shaped by behaviors that are typically successful in an individual's social environment (Rand et al., 2014). For young children, the primary social environment is the family, where cooperation is generally the most advantageous strategy (Eisenberg et al., 2006; Wentzel, 2014). Therefore, a standard reading of the SHH would predict the internalization of a strong, stable prosocial heuristic from an early age—a prediction that is entirely consistent with the authors' findings.
The authors' argument that the SHH predicts a developmental shift toward selfish intuitions is not a core tenet of the theory and would only hold if school-age environments were so overwhelmingly competitive as to systematically "unlearn" early prosocial heuristics. As such, the study does not provide the critical test it claims. The results are compatible with both a nativist account and an SHH account. The manuscript should be reframed to acknowledge this, shifting the focus from adjudicating between these theories to detailing the novel developmental dynamic between a stable prosocial intuition and emerging deliberative reasoning.

2. Interpretation of Latent Factors
The data-driven factors are a major strength, but their psychological interpretation requires stronger justification.
The "Prosociality" Factor: This factor combines cooperation (PGG), altruism (DG), and honesty (DEG) with the acceptance of low offers in the Ultimatum Game (UG). Including the acceptance of unfairness—a lack of costly punishment—in a "Prosociality" factor is conceptually problematic. Prosocial behavior is typically defined as voluntary acts intended to benefit others (Eisenberg et al., 2006), and costly punishment is often considered a key mechanism for maintaining, not undermining, cooperation. An alternative interpretation is that this factor captures "low strategic sophistication" or compliance, which declines with age across all tasks. This concern is amplified by the high uniqueness coefficients for many of the task variables (e.g., PGG2, PGG3, DG2, UG0, UG1) reported in Table B3, which are often above 0.7 or 0.8. High uniqueness suggests that a large portion of a variable's variance is not explained by the common factors, weakening the claim that the factor represents a single, robust underlying trait.
The "Acquiescence" Factor: This factor, defined by accepting all UG offers, is interpreted as a general willingness to accept others' decisions. A more parsimonious explanation is that it reflects the development of task competence. Young children may not fully grasp the strategic nature of the UG, and the age-related decline in this factor may simply track their developing understanding of fairness norms and the power of rejection (McAuliffe et al., 2013).

3. Methodological Validity and Generalizability
Time-Constraint Manipulation: The validity of using a 10-second time pressure manipulation to isolate "intuition" in preschoolers (ages 3-4) is a significant concern. For this age group, the tasks are already cognitively demanding, and time pressure may induce cognitive overload, stress, or confusion rather than preventing deliberation (Ackerman & Lauterman, 2012; Walczyk & Griffith-Ross, 2006). Furthermore, the literature on time pressure in children is contradictory; while some studies find it increases prosocial sharing (Plötner et al., 2021), others find it inhibits prosocial helping, arguing that helping requires deliberation (Zha et al., 2025). This conflicting evidence must be addressed.
Generalizability: The study was conducted exclusively with children from Milan, Italy, a WEIRD (Western, Educated, Industrialized, Rich, Democratic) society. Given substantial evidence of cross-cultural variation in prosocial development (House et al., 2013; Kärtner, 2015), the authors must temper their broad claims about "human development" and explicitly discuss this limitation.

Minor Concerns
Figure Clarity: Figure 2 is central to the paper's claims but is very dense. The main text should guide the reader through the key takeaways from each panel more explicitly.
Comprehension Checks: The authors should report whether comprehension check failure rates differed between the time pressure and time delay conditions, as this would speak directly to the cognitive load imposed by the manipulation.

This is a potentially high-impact study with a rich dataset and innovative methods. However, the theoretical claims are currently overstated, and the interpretation of the core findings requires significant refinement. The manuscript can be substantially strengthened if the authors reframe the theoretical argument, provide a more nuanced discussion of the latent factors (including alternative interpretations), and thoroughly address the methodological limitations concerning the time-pressure manipulation and cultural specificity.

References

Ackerman, R., & Lauterman, T. (2012). Taking a break: The effects of sleep and interruptions on task-switching performance. Journal of Experimental Psychology: Applied, 18(2), 140–150.

Eisenberg, N., Fabes, R. A., & Spinrad, T. L. (2006). Prosocial development. In W. Damon & R. M. Lerner (Series Eds.) & N. Eisenberg (Vol. Ed.), Handbook of child psychology: Vol. 3. Social, emotional, and personality development (6th ed., pp. 646–718). Wiley.

Grueneisen, S., & Warneken, F. (2022). The development of prosocial behavior: From sympathy to strategy. Current Opinion in Psychology, 44, 144-150.

House, B. R., Silk, J. B., Henrich, J., Barrett, H. C., Scelza, B. A., Boyette, A. H.,... & Laurence, S. (2013). Ontogeny of prosocial behavior across diverse societies. Proceedings of the National Academy of Sciences, 110(36), 14586-14591.

Kärtner, J. (2015). A cultural perspective on prosocial development. Courant Research Centre ‘Text-Structures’, University of Göttingen.

Margoni, F., Nava, F., Sotis, C., Levy, M. R., Capraro, V., & Nava, E. (2025). The Rise of Deliberative Prosociality. PsyArXiv.

McAuliffe, K., Blake, P. R., Kim, G., Wrangham, R. W., & Warneken, F. (2013). Social influences on inequity aversion in children. PLoS ONE, 8(12), e80966.

Plötner, M., Hepach, R., Over, H., Carpenter, M., & Tomasello, M. (2021). Young children share more under time pressure than after a delay. PLoS ONE, 16(10), e0248121.

Rand, D. G., Greene, J. D., & Nowak, M. A. (2012). Spontaneous giving and calculated greed. Nature, 489(7416), 427–430.

Rand, D. G., Peysakhovich, A., Kraft-Todd, G. T., Newman, G. E., Wurzbacher, O., Nowak, M. A., & Greene, J. D. (2014). Social heuristics shape intuitive cooperation. Nature Communications, 5, 3677.

Walczyk, J. J., & Griffith-Ross, D. A. (2006). A test of the effects of time pressure on creative versus convergent problem solving. Creativity Research Journal, 18(2), 205-214.

Wentzel, K. R. (2014). Prosocial behaviour and schooling. In Child Encyclopedia on Early Childhood Development. Centre of Excellence for Early Childhood Development.

Zha, Z., Chen, C., Zhang, R., & Zhang, W. (2025). The effect of time pressure and ego depletion on young children's helping behavior. Frontiers in Psychology, 16, 1584248.

Reviewer #3 (Remarks to the Author):

Strengths:
- This is a quite a large study in terms of the sample size, age-range, and breadth in measures.
- The topic is of general interest in the behavioral sciences, connecting discussions about adult psychology and developmental psychology (but see some suggestions for improvement below).
- Despite the number of measures, the results are described succinctly and fairly easy to follow. The authors make good use of the supplemental material.
- The experimental tasks are adequate and build on previously validated paradigms. Some more information on execution and additional analyses are needed, however.

Weaknesses
- The introduction makes distinction between nativist accounts and the Social Heuristics Hypothesis. My two main reservations are that what predictions the Social Heuristics Hypothesis makes regarding the emergence of cooperative behaviors in children as grounded in empirical findings about child development. The paragraph on p. 3 is purely speculative, not making any connection to the developmental literature. The false dichotomy between a social heuristics approach focused on learning and experience and nativist approaches continues. This sounds like the nature-nurture debate long buried and now revived. Note also that the study is not set up to test any claims about early-emerging capacities, given that the youngest children of the sample are already 3 years old (researchers debating this issue test infants and toddlers).
- The second reservation is that more nuance is needed in the introduction of developmental accounts about the origins of prosociality. For one, the authors measure both children’s behaviors and their expectations / representations of social partners, which are ontologically different. Some of the cited findings and claims concern what children THINK about cooperative behaviors, how they EVALUATE e.g. helpers and hinders, while others are about what MOTIVATES children’s sharing and helping behavior. Please look up discussions of the knowledge-behavior gap in the development of cooperation (e.g. Blake et al.). Related to this, more theoretical nuance is needed to make predictions about what develops in children, i.e. what aspects are innate and what aspects are learned. Warneken et al. has written an Annual Reviews of Psychology article on this, providing a framework that might be helpful.
- More attention to the extant developmental literature on intuitive vs. deliberate processes is needed. See the work by Corbit et al, and Grossmann et al., which also includes public good games with time manipulation.
- The title uses “prosociality” as the umbrella term, but then only one of the tasks is about prosociality. Use “cooperation” instead as the overarching concept?
- Several questions remain regarding methods and presentation of results:
- How were children’s responses recorded?
- No information on reliability coding seems to have been provided.
- Please state that condition assignment was randomized on p. 12 (“half under time pressure and half under time delay”). This is currently only mentioned for each individual task.
- The figure states that the partners were “fictitious”, but the method section describes them as real partners. The Appendix adds to the confusion as it states that “the 3 partners are not real children but small closed boxes with images of children’s faces” — which presumably is a description and not something that the child participants were told. There is some video recording of at least one child, but was there also an assessment that children believed the children were real? For the moral dilemma task, we are apparently dealing with purely hypothetical scenarios or imagined others. All of this needs to be sorted out and described more clearly. It should also be addressed to what extent children believed they were interacting with real

children, including empirical data.
- Figure 1 is helpful in schematically representing the different tasks. I was confused about the insertion of "boxes used to depict the partners in all games". The schematic drawing might be more effective if it featured samples of the actual stimuli rather than the current old-fashioned representations of what look like white boys with buzz cuts from the 1950s.

Version 1:

Decision Letter:

5th November 2025

Dear Dr Margoni,

RE: "Stable Intuition and the Rise of Deliberative Prosociality in Childhood"

Thank you for submitting your revised manuscript and for all your work on the revision.

Although your manuscript has been revised in response to reviewer comments, there are some remaining concerns that we would like you to address before we send it back to the reviewers. Apologies that this wasn't made sufficiently clear in our previous decision letter, however, one point we'd like to clarify is the need to explicitly acknowledge in the manuscript the shift in your theoretical framing that occurred during peer review.

To ensure transparency for readers, we ask that you briefly explain in the manuscript regarding this evolution: although you originally aimed to distinguish between the SHH and nativist accounts, during peer review you came to recognize that the results do not allow for such distinction. You have therefore shifted focus to presenting the developmental patterns and clarifying how these findings constrain what the SHH and nativist approaches can predict.

This will help contextualize your revised interpretation and clarify the scope of your conclusions.
We're sorry for any confusion caused by the earlier communication and appreciate your continued engagement in refining the manuscript.

Please use the link below to submit your revised manuscript and related files:

Link Redacted

Note: This URL links to your confidential home page and associated information about manuscripts you may have submitted, or that you are reviewing for us. If you wish to forward this email to co-authors, please delete the link to your homepage.

Thank you in advance for attending to these requests and I look forward to receiving your revised manuscript.

Sincerely,

[redacted]
Nature Human Behaviour

Version 2:

Decision Letter:

Our ref: NATHUMBEHAV-25062855B

15th December 2025

Dear Dr. Margoni,

Thank you for submitting your revised manuscript "Stable Intuition and the Rise of Deliberative Prosociality in Childhood" (NATHUMBEHAV-25062855B). It has now been seen by the original referees and their comments are below. As you can see, the reviewers find that the paper has improved in revision. We will therefore be happy in principle to publish it in Nature Human Behaviour, pending minor revisions to satisfy the referees' final requests and to comply with our editorial and formatting guidelines.

We are now performing detailed checks on your paper and will send you a checklist detailing our editorial and formatting requirements within two weeks. Please do not upload the final materials and make any revisions until you receive this additional information from us.

Please do not hesitate to contact me if you have any questions.

Sincerely,

[redacted]
Nature Human Behaviour

Reviewer #1 (Remarks to the Author):

I have reviewed the revised manuscript carefully, along with the authors' detailed responses to my previous comments. I am satisfied that the authors have thoroughly and thoughtfully addressed all concerns raised in the earlier round. I have no further comments. I believe this manuscript will make a valuable contribution to the field, and I look forward to seeing it in print.

Reviewer #2 (Remarks to the Author):

The authors have submitted a substantially improved revision that effectively addresses the major theoretical and methodological concerns raised in the previous round of review. The pivotal shift in theoretical framing—moving from a rigid adjudication between the Social Heuristics Hypothesis (SHH) and Nativism to a framework that uses developmental data to constrain the predictions of both theories—is scientifically sound and resolves the false dichotomy noted previously.

Methodologically, the addition of extensive robustness checks in Appendix B is the revision's strongest feature. By demonstrating that the core "closing gap" trajectory holds even when excluding the youngest participants (Table B12) and the most complex task (Table B16), the authors have successfully mitigated concerns regarding task comprehension and the cognitive validity of the time-pressure manipulation. The evidence that time pressure does not induce random responding in 3-4 year olds (Table B17) is particularly convincing.

I recommend acceptance, provided the authors address the following minor issues to fully comply with Nature Human Behaviour reporting standards.

Minor Comments for Revision

1. Statistical Reporting (Exact P-values): The manuscript currently relies on inequalities (e.g., $p < .001$, $p = .210$) and asterisks in tables to denote significance. This does not strictly adhere to the journal's guidelines. Please revise the main text and all Supplementary Tables to report **exact p-values** (e.g., $p = .042$) wherever possible.

2. Bayesian Prior Justification: In the Results section, you state that "flat priors were used" for the Bayesian analysis. Please add a brief sentence justifying this choice (e.g., to maximize the influence of the likelihood function given the sample size) to ensure transparency for readers less familiar with default Bayesian settings.

3. Sample Specificity: While the addition of the "WEIRD" limitation is welcome, please expand this briefly to acknowledge the specific cultural context of the sample (Northern Italy). Acknowledging specific cultural nuances regarding trust and social capital adds necessary precision beyond the generic "WEIRD" label.

4. Clarification of the "Acquiescence" Factor: To strengthen the defense of the "Acquiescence" factor against the "incompetence" interpretation, I suggest explicitly mentioning in the main Discussion that this factor loads negatively on deception (Table B3). This is a strong data point indicating that "acquiescent" children are not merely clicking randomly or confused (which might lead to random lying), but are actively complying with truth-telling norms. Highlighting this in the text will solidify the argument.

Reviewer #3 (Remarks to the Author):

The revision resulted in a much improved manuscript. Thank you for a very responsive revision. The clarification questions about the methods have been addressed and the explanations are much clearer now.

I have to remaining concerns. There is some clarification on there use of terminology and the underlying concepts, but there are still some inconsistencies. The authors state the “prosociality” is used as a general term and altruism, cooperation etc. are specific forms of prosociality. However, in several places “cooperation” or is used as a general term. On p. 1011-, cooperation and prosociality are used interchangeably. On p. 2, it is stated that intuitive processes promote “cooperation” but

according to the usage before, “cooperation” is measured by the PGG, not the other tasks. Is it the case that the authors used “cooperation” to refer to social dilemmas, where the decision is between “cooperation” and “defection”? I acknowledge that terminology is a thorny issue and there is no perfect solution because different fields apply different theoretical frameworks. I think the only solution is to be clearer on the assumptions that are being made and also be clear that there is no 1:1 mapping go a task and a concept. For example, a DG is not a straightforward measure of “altruism” as also fairness consideration could play a role. “Cooperation” is not the same as a choice in a PD or PGG.

I do not understand this sentence: “The
evidence further indicates that the translation of prosocial motivations into actions
develops more gradually than expectations and preferences or evaluative capacities,
with helping and sharing emerging first and cooperative skills emerging later”

A second issue is that while some contact is made with theories of child development, these parts are still very much underdeveloped. It’s not the case that prosociality is either increasing, decreasing or remaining stable across development. The debate is actually about the origins of different processes and how prosocial behaviors become more selective or more expansive. See the discussions between Wynn and Bloom for argue for a early selectivity and a broadening over development versus the work by scholars like Marshall, Warneken, Wilks suggesting that prosocial behaviors (and moral concerns) start out broader and then become more selective over development. This would be a much better and more contemporary theoretical setup than the current suggestion that there this no change (who really believes that?) or decline versus increase.

Open Access This Peer Review File is licensed under a Creative Commons Attribution 4.0 International License, which permits use, sharing, adaptation, distribution and reproduction in any medium or format, as long as you give appropriate credit to the original author(s) and the source, provide a link to the Creative Commons license, and indicate if changes were made.
In cases where reviewers are anonymous, credit should be given to 'Anonymous Referee' and the source.
The images or other third party material in this Peer Review File are included in the article’s Creative Commons license, unless indicated otherwise in a credit line to the material. If material is not included in the article’s Creative Commons license and your intended use is not permitted by statutory regulation or exceeds the permitted use, you will need to obtain permission directly from the copyright holder.
To view a copy of this license, visit https://creativecommons.org/licenses/by/4.0/

Reviewer #1

I see several strengths with this study: the authors have identified an important empirical question and used a rigorous methodological and analytic approach with a large (well-justified) sample size. My main questions centre on how this study can inform the predictions of the Social Heuristics Hypothesis and Nativist theories (which serve as the theoretical foundation), and the extent to which the youngest participants understood the more complex tasks, such as the public-goods game.

Theoretical framing

P. 3 In my reading of several SHH papers, there is no explicit developmental prediction, but there is an assumption that heuristics must be acquired through social experience, and so it makes sense that social heuristics would be acquired during early and middle childhood. I understand the logic, offered by the authors, that early social environments within the family may be more supportive of prosocial heuristics compared to the social environments children encounter when entering school, and so the social heuristics acquired may shift from prosocial to self-serving. At the same time, these claims about the type of social environment children are likely to encounter at various developmental stages are not self-evident and so should be made with supporting evidence.

An alternative possibility is that children find themselves in relatively stable social environments (in terms of supporting prosocial or self-serving heuristics) throughout the preschool-to-school-age years. This could lead to developmental stability in social intuitions across the age range studied here, in which case developmental continuity would be equally consistent with the Nativist and SHH accounts. If the acquired heuristics are prosocial, then the same developmental continuity predicted by the authors' in their framing of the nativist account may be observed, in which case it would not be possible to differentiate between the two accounts.

I think it might help to lay out specifically which pattern of evidence in the current study would align with SHH and which would align with a nativist account. A tricky thing about SHH here is that it is hard to imagine a pattern that could falsify it without a way to test alignment between children's social intuition and their social environment. Similarly, most contemporary nativist theories also allow for developmental change after early emergence and a role for social learning, so it is not immediately obvious how these perspectives can be adjudicated based on the pattern of findings in this study. Making explicit predictions about expected patterns would help.

We fully agree with these observations and have rewritten the Introduction in line with the reviewers' suggestions. Specifically, we have shifted the focus from testing predictions and adjudicating between SHH and nativism to a) detailing the development of prosocial intuitions and deliberative social decision-making, and b) clarifying the empirical scope of the SHH and nativist accounts. We now frame the paper as a description of developmental processes that, in turn, help constrain our theoretical frameworks. As the reviewer noted, the SHH allows for at least two possible developmental trajectories: continuity, where prosocial heuristics remain stable across childhood, or transition, where self-serving heuristics emerge in middle childhood. Based on our data, we conclude that prosocial heuristics remain stable up to 10 years of age.

Comprehension for youngest participants

One concern I have is with the level of task comprehension that the youngest participants are likely to have. This seems particularly important when decision-time manipulations are used, as there is a potential for time pressure to exacerbate comprehension problems and, in turn, drive condition effects in unintended ways. I'll focus on the PGG, as this was the most complex of the resource-allocation tasks. The comprehension check verified that children understood that the candies they kept went to themselves and the ones they put in the common bowl went to everyone. But would the three-year-olds have understood the more complex nuances of the PGG? For example, even though the candies are doubled when put in the common bowl, it is better for me to just keep the candies and hope that others contribute if I want to self-maximize. Basically, do they understand that donations to the group are leveraged to provide a larger benefit to the group than keeping the candies can provide to themselves, but that these cooperative decisions are personally costly and the way to self-maximise is to keep everything for yourself? My impression is that the comprehension check showed that participants understood the mechanics of who gets what candies (similar to what would be required for a DG), but

it seems ambiguous whether they would understand these nuances of the PGG. Are there any additional checks that were not fully described in the manuscript? Or is there any precedent for using a PGG with children this young (I'm not aware of any and couldn't identify any in a quick search). It may also help to explain how the evaluation was made that all tasks are age-appropriate for the youngest participants.

We agree that comprehension checks cannot fully capture participants' grasp of all strategic nuances or the optimal play in a task. Comprehension checks were designed to ensure understanding of task mechanics without requiring children to identify the "optimal" strategy or anticipate others' play. For the PGG in particular — the most complex task and one without prior protocols for 3-year-olds — we took extra care by supplementing standard verbal instructions with an additional video demonstration.

Importantly, in the revised version of the article, we include a series of robustness checks that provide three pieces of empirical evidence suggesting that it is unlikely that our results are driven by lack of comprehension. We elaborate on these pieces of evidence below.

(1) Youngest children under time pressure are not more likely to make random errors

We find no systematic tendency for time pressure to move 3–4-year-olds towards random choice. In 5 of the 9 tasks, their behaviour in the fast condition is farther from random choice than their behaviour in the slow condition. For 2 of these 5, a one-sided t-test shows that behaviour in the time pressure condition is significantly farther away from random choice. In UG0 and UG1, behaviour in the time pressure condition is significantly closer to random choice, but that does not extend to the other UG tasks (or any other task). We report on this in Appendix B6 (Table B17).

(2) The main results are robust to excluding youngest children

The main findings remain robust when excluding the youngest children from the analysis. Specifically, excluding 3–4-year-olds from the sample, we find a three-factor structure with factor loadings closely aligned with those from the main specification (Tables B11) and, importantly, we still find a significant interaction condition (fast, slow) x age in the Prosociality factor (Table B12). This suggests that our results are coming from systematic behaviour across all age groups, and not just the youngest children.

(3) The main results are robust to excluding the PGG

We agree with the reviewer that the PGG may have been the most difficult game to understand. To address this concern, we have replicated the analysis omitting all PGG tasks and the associated belief elicitations. Our results are robust to the change, with the factor loadings on the two retained factors closely aligned with those from the main specification (Table B15). Additionally, our estimates for the impact of condition and age on the factors are unchanged (Table B16). This provides reassurance on the validity of our factor approach, which does not appear to rely on specific tasks (i.e., PGG).

Minor points

P. 4 I realise there are significant word constraints for the manuscript, but it might be helpful to provide additional information about the development of intuitive cooperation in past literature. There are additional studies (not cited e.g Grossmann et al., 2020; Chajes et al., 2022, not exhaustive) that have examined this question in the developmental literature, though most use decision-time correlations rather than manipulations. If the authors have decided to selectively cover only studies using decision-time manipulations, that could probably be stated very quickly as the literature is introduced.

Thank you for this comment. We deliberately restricted our review to studies using decision time manipulations, as these provide stronger evidence than correlational studies. Following the reviewer's suggestion, we now acknowledge this point when introducing the literature. Given space constraints, correlational work is mentioned only in passing. This addition appears already in the second paragraph of the Introduction (p. 2).

P. 5 Were judgments about other players' contributions in the PGG made under the decision-time manipulation? If not, does it make sense to look for a condition effect for Social Optimism if the primary factor is not subject to a condition manipulation? I may be missing something here, so would appreciate

clarification.

Judgments were made immediately after contributions, without time pressure. We now clarify this in the Methods (end of p. 15):

> "These judgments were made without time constraints, and children in both conditions had sufficient time to reason about their partners' likely contributions. The goal was to evaluate children's beliefs about how others would behave under time pressure or delay, rather than their own pressured decision making."

We note that examining interactions remains meaningful, as these judgments reflect children's beliefs about decisions made under manipulated cognitive modes. This is now also stated for example on p. 7 in the Results ("assuming that others decided under time pressure or time delay"), but we acknowledge that these beliefs were not directly manipulated.

P. 10 It would be good to mention explicitly that the direction of the relation between PGG contributions and predictions about the contributions of others is not clear, just that there is an association. I think this point is alluded to with "and/or vice versa", but the conclusions are more directional. You could also cite Corbit et al., 2023, Dev Sci; they found a similar association, though only when decisions were made under time pressure.

We fully agree. Following this suggestion, we now discuss the relationship between contributions and expectations about others' contributions in the PGG as an association, without implying a clear causal direction (p. 11, second paragraph). For instance, we note that "children's expectations of high contributions from the partners are positively associated with their own likelihood of contributing" and that "this association may reflect a foundational aspect of human social behaviour: individuals' expectations about others' cooperation and their own willingness to cooperate are closely intertwined, potentially influencing each other through mechanisms such as conditional cooperation." We also cite Corbit's work in this paragraph.

P. 10 I believe SHH would be agnostic about whether intuition favours self-serving or prosocial behaviours in older children. This would depend on the social environment that the older children encounter, and it seems important to show that this environment supports self-serving heuristics in order to claim that SHH would predict this. I may have missed a paper that makes these sorts of predictions from SHH, but if this is based on about the authors' own assertion about children's social environments, then I believe these conclusions could be made more cautiously.

We agree, and we now discuss that SHH can generate multiple predictions depending on the type of social environment children are inserted. Please see p. 4 of the Introduction.

PP. 10–11 I find the claim that "deliberate prosociality rises with age" difficult to interpret because the study's two-condition design (pressure vs. delay) only allows relative rather than absolute inferences. Prior developmental work shows a general increase in prosocial behaviour across this age range, so the upward trend observed in the delay condition could simply mirror that baseline trajectory rather than reveal a unique effect of deliberation. Without a neutral-timing control, we cannot disentangle whether fast decisions elevate prosociality, slow decisions depress it, or both. Once prosociality rates converge across the two conditions at about eight years of age, the data indicate merely that the timing manipulation no longer has a significant effect on behavior (relative to each other, and we don't that what might be happening relative to baseline). In short, the current design more strongly supports conclusions about the relative contributions of intuitive versus deliberative processes, but the separate developmental trajectories for each process are not clear.

Thank you for this useful comment. We have revised the discussion (pp. 11–12) to address these concerns. We use the term *deliberative prosociality* for behaviour observed in the time delay condition, and *intuitive prosociality* for behaviour in the time pressure condition. As we do not include a time neutral condition, we only examine behaviour within and across the two experimental frames. The delay condition explicitly imposes a deliberative mode, allowing a distinct test of prosociality under reflection. We now note on p. 13, while discussing limitations, that the study does not directly address behaviour in a neutral timing context.

Reviewer #2

Major Concerns

1. Theoretical Framing: Nativism vs. Social Heuristics Hypothesis (SHH)

The authors frame their study as a critical test between nativist accounts and the Social Heuristics Hypothesis (SHH), concluding that their findings support nativism while being "more difficult to reconcile" with the SHH. This framing relies on a narrow interpretation of the SHH. The SHH posits that intuitions are heuristics shaped by behaviors that are typically successful in an individual's social environment (Rand et al., 2014). For young children, the primary social environment is the family, where cooperation is generally the most advantageous strategy (Eisenberg et al., 2006; Wentzel, 2014). Therefore, a standard reading of the SHH would predict the internalization of a strong, stable prosocial heuristic from an early age—a prediction that is entirely consistent with the authors' findings.

The authors' argument that the SHH predicts a developmental shift toward selfish intuitions is not a core tenet of the theory and would only hold if school-age environments were so overwhelmingly competitive as to systematically "unlearn" early prosocial heuristics. As such, the study does not provide the critical test it claims. The results are compatible with both a nativist account and an SHH account. The manuscript should be reframed to acknowledge this, shifting the focus from adjudicating between these theories to detailing the novel developmental dynamic between a stable prosocial intuition and emerging deliberative reasoning.

We fully agree with these observations. In response, we have reframed the Introduction and we have clarified that the SHH is consistent with different predictions regarding the emergence (or not) of self-serving intuitions in middle childhood (see p. 4). More generally, the Introduction now frames the study's main aim as describing developmental processes, which are in turn interpreted using the SHH and the nativist account, without creating a contrast within the two. Thus, we shifted the focus from directly testing theoretical predictions to using our data to inform and refine theoretical expectations.

2. Interpretation of Latent Factors

The data-driven factors are a major strength, but their psychological interpretation requires stronger justification. The "Prosociality" Factor: This factor combines cooperation (PGG), altruism (DG), and honesty (DEG) with the acceptance of low offers in the Ultimatum Game (UG). Including the acceptance of unfairness—a lack of costly punishment—in a "Prosociality" factor is conceptually problematic. Prosocial behavior is typically defined as voluntary acts intended to benefit others (Eisenberg et al., 2006), and costly punishment is often considered a key mechanism for maintaining, not undermining, cooperation. An alternative interpretation is that this factor captures "low strategic sophistication" or compliance, which declines with age across all tasks. This concern is amplified by the high uniqueness coefficients for many of the task variables (e.g., PGG2, PGG3, DG2, UG0, UG1) reported in Table B3, which are often above 0.7 or 0.8. High uniqueness suggests that a large portion of a variable's variance is not explained by the common factors, weakening the claim that the factor represents a single, robust underlying trait.

Thank you for this thoughtful comment. We refer to the first factor as "Prosociality", arguing that it captures general prosocial tendencies, specifically defined as behaviours that benefit others (see p. 5, last sentence). In our one-shot context, accepting low offers in the UG does benefit the proposer, and therefore it is a prosocial behaviour according to this definition. Thus, in the context of our one-shot interactions, the Prosociality factor primarily reflects a behaviour that benefits others in the short term, rather than a principled commitment to enforcing fairness norms or a punishment mechanism aimed at increasing long-term cooperation. Importantly, we do not equate this factor with "cooperation". Rather, it represents general prosociality: a willingness to increase others' total payoff in the short term without necessarily knowing or maximizing what is best for the group or society more broadly and in the long term. We clarify this both in the Results and Discussion. We also note that all tasks in our study involved one-shot interactions with putatively different partners, and thus punishments do not influence long-term cooperation. Unlike in repeated interactions, rejecting low offers can only harm others.

As for uniqueness concerns, we agree that high uniqueness values can indicate poor measurement precision. To evaluate the impact of high uniqueness, we conducted a robustness analysis by repeating

our main analyses while omitting tasks with uniqueness coefficients above 0.90. This criterion excluded UG2, MDF, and MDB. Because such an approach excluded the noisiest tasks, factor loadings were largely unaffected, but predicted factors were less noisy. Consequently, our regression analyses yield the same conclusions. Further lowering the threshold to 0.8 excludes 9 of the 19 tasks and the Acquiescence factor no longer achieves an eigenvalue above 1. However, the results for Prosociality and Social Optimism are robust to this threshold as well. To avoid p-hacking the specification, we decided to stick with our original analysis. However, we have now included these robustness checks in Appendix B5 (Table B13). Please also see Table B14 for robustness analysis regressions**.** The exclusion of high-uniqueness variables actually strengthens our findings by reducing measurement noise. The robustness analysis confirms our main results across all three factors. We have only included the 0.9 threshold in the Appendix but can add the 0.8 threshold if requested.

The "Acquiescence" Factor: This factor, defined by accepting all UG offers, is interpreted as a general willingness to accept others' decisions. A more parsimonious explanation is that it reflects the development of task competence. Young children may not fully grasp the strategic nature of the UG, and the age-related decline in this factor may simply track their developing understanding of fairness norms and the power of rejection (McAuliffe et al., 2013).

Thank you for this comment. Please note that our game was one-shot and thus lacked strategic components. We apologize if this was not clear. We now clarify this in the manuscript. Importantly, the “acquiescence” factor loads positively not only on accepting unfair offers (smaller than 2), but also on accepting offers equal to or larger than 2. Since this factor loads positively on accepting *all* types of offers, we interpret it not as reflecting fairness considerations, but rather as a general tendency to accept any offer; hence our use of the term *acquiescence*. Moreover, rejecting advantageous offers is not an indicator of competence, and therefore this pattern works against the alternative interpretation. In the revised version of the article, we discuss potential alternative interpretations in the Discussion.

3. Methodological Validity and Generalizability

Time-Constraint Manipulation: The validity of using a 10-second time pressure manipulation to isolate "intuition" in preschoolers (ages 3-4) is a significant concern. For this age group, the tasks are already cognitively demanding, and time pressure may induce cognitive overload, stress, or confusion rather than preventing deliberation (Ackerman & Lauterman, 2012; Walczyk & Griffith-Ross, 2006).

We agree this is an important concern. In the revised version of the article, we have included several robustness checks that provide three pieces of evidence suggesting that our results are not driven by lack of understanding among youngest children under time pressure.

(1) Youngest children under time pressure are not more likely to make random errors

We compared the results for 3–4-year-olds under time pressure vs time delay, to see which, if any, is further than random. We found no systematic tendency for time pressure to move these participants towards random choice (Table B17). In 5 of the 9 tasks, behaviour in the fast condition is farther from random choice than behaviour in the slow condition. For 2 of these 5, a one-sided t-test shows that behaviour in the time pressure condition is significantly farther away from random choice. In UG0 and UG1, behaviour in the time pressure condition is significantly closer to random choice, but that does not extend to the other UG tasks (or any other task). We report on this in Appendix B6.

(2) The main results are robust to excluding youngest children

Excluding 3–4-year-olds from the sample, we find a three-factor structure with factor loadings closely aligned with those from the main specification (Tables B11) and, importantly, we still find a significant interaction condition (fast, slow) x age in the Prosociality factor (Table B12). This suggests that our results are coming from systematic behaviour across all age groups, and not just the youngest children.

(3) The main results are robust to excluding the PGG

We have replicated the analysis omitting all PGG tasks (arguably the most difficult tasks) and the associated belief elicitations. Our results are robust to the change, with the factor loadings on the two retained factors closely aligned with those from the main specification (Table B15). Additionally, our

estimates for the impact of condition and age are unchanged (Table B16). This provides reassurance on the validity of our factor approach, which does not appear to rely on specific tasks (i.e., PGG).

Furthermore, the literature on time pressure in children is contradictory; while some studies find it increases prosocial sharing (Plötner et al., 2021), others find it inhibits prosocial helping, arguing that helping requires deliberation (Zha et al., 2025). This conflicting evidence must be addressed.

Thank you for pointing this out. We now reference the contrasting findings in the literature on p. 11.

Generalizability: The study was conducted exclusively with children from Milan, Italy, a WEIRD (Western, Educated, Industrialized, Rich, Democratic) society. Given substantial evidence of cross-cultural variation in prosocial development (House et al., 2013; Kärtner, 2015), the authors must temper their broad claims about "human development" and explicitly discuss this limitation.

We have now added this point as a limitation and future direction in the Discussion (see p. 13), and overall we have tempered our writing.

Minor Concerns

Figure Clarity: Figure 2 is central to the paper's claims but is very dense. The main text should guide the reader through the key takeaways from each panel more explicitly.

We now discuss the figure in greater detail throughout sections 2.1 and 2.2.

Comprehension Checks: The authors should report whether comprehension check failure rates differed between the time pressure and time delay conditions, as this would speak directly to the cognitive load imposed by the manipulation.

We now report failure rates for comprehension checks by age and condition in Appendix B1. We note that children under time pressure were not significantly less likely to pass the comprehension questions; if anything, descriptive statistics point in the opposite direction.

This is a potentially high-impact study with a rich dataset and innovative methods. However, the theoretical claims are currently overstated, and the interpretation of the core findings requires significant refinement. The manuscript can be substantially strengthened if the authors reframe the theoretical argument, provide a more nuanced discussion of the latent factors (including alternative interpretations), and thoroughly address the methodological limitations concerning the time-pressure manipulation and cultural specificity.

We are thankful for the comments and suggestions we have received, which have greatly helped us in revising the paper.

Reviewer #3

Strengths: This is quite a large study in terms of the sample size, age-range, and breadth in measures. The topic is of general interest in the behavioral sciences, connecting discussions about adult psychology and developmental psychology (but see some suggestions for improvement below). Despite the number of measures, the results are described succinctly and fairly easy to follow. The authors make good use of the supplemental material. The experimental tasks are adequate and build on previously validated paradigms. Some more information on execution and additional analyses are needed, however.

Weaknesses

The introduction makes distinction between nativist accounts and the Social Heuristics Hypothesis. My two main reservations are that what predictions the Social Heuristics Hypothesis makes regarding the emergence of cooperative behaviors in children as grounded in empirical findings about child development. The paragraph on p. 3 is purely speculative, not making any connection to the developmental literature. The false dichotomy between a social heuristics approach focused on learning and experience and nativist approaches continues. This sounds like the nature-nurture debate long buried and now revived. Note also that the study is not set up to test any claims about early-emerging capacities, given that the youngest children of the sample are already 3 years old (researchers debating this issue test infants and toddlers).

We agree with these observations. In line with similar points raised by the other two reviewers, we have revised the Introduction to avoid framing our study as adjudicating between SHH and nativism, since these theories can, in fact, generate overlapping predictions. Instead, we now present our work primarily as an effort to characterize the development of intuitive and deliberate social decision making, with the additional aim of using our data to refine the empirical scope and possible predictions of the main theoretical frameworks guiding our Introduction.

The second reservation is that more nuance is needed in the introduction of developmental accounts about the origins of prosociality. For one, the authors measure both children's behaviors and their expectations / representations of social partners, which are ontologically different. Some of the cited findings and claims concern what children THINK about cooperative behaviors, how they EVALUATE e.g. helpers and hinders, while others are about what MOTIVATES children's sharing and helping behavior. Please look up discussions of the knowledge-behavior gap in the development of cooperation (e.g. Blake et al.). Related to this, more theoretical nuance is needed to make predictions about what develops in children, i.e. what aspects are innate and what aspects are learned. Warneken et al. has written an Annual Reviews of Psychology article on this, providing a framework that might be helpful.

We also agree with this second concern. We have revised the Introduction, and in the updated paragraph on p. 3 we now structure the literature review around three strands of evidence: (i) studies on expectations about others' behaviour, (ii) studies on preferences or evaluations, and (iii) studies on motivations or behaviours. We also cite the recommended works by Blake et al. and Warneken.

More attention to the extant developmental literature on intuitive vs. deliberate processes is needed. See the work by Corbit et al, and Grossmann et al., which also includes public good games with time manipulation.

Thank you for pointing to this literature. We cite Corbit et al. on p. 2. Assuming the reviewer is referring to Grossmann, Missana, & Vaish (2020), Cognition, 196, 104144, we note that this is not a manipulation study; we thus include it among the studies relying solely on correlational measures (p. 2). The literature paragraph has been expanded in the suggested direction.

The title uses "prosociality" as the umbrella term, but then only one of the tasks is about prosociality. Use "cooperation" instead as the overarching concept?

We would say that only one task strictly addresses "cooperation", namely, the PGG. Importantly, we do not use "Prosociality" as a broad umbrella term, but when capitalised, it specifically refers to the construct identified through our factor analysis. This factor loads on behaviours that increase the others' payoff; these behaviours align with standard definitions of prosociality (Eisenberg & Mussen, 1989),

which is why we chose this term. We have carefully reviewed the manuscript to ensure consistency and now define "prosociality" explicitly at the outset (p. 2, second sentence).

Several questions remain regarding methods and presentation of results:

How were children's responses recorded?

We have added this information to the methods section (p. 14, beginning of section 4.2): "… their responses were recorded by the experimenter using paper and pen."

No information on reliability coding seems to have been provided.

We now clarify in the manuscript that children's responses were recorded by the experimenters using paper and pen (p. 14). During each session, experimenters simply registered the number of candies contributed by each participating child, and no video recordings were made. Because the task involved only a straightforward numerical response and there were no subjective judgments to code, inter-rater reliability was not applicable in this context.

Please state that condition assignment was randomized on p. 12 ("half under time pressure and half under time delay"). This is currently only mentioned for each individual task.

Thank you, the information was already noted at the end of 4.1 section, but following the reviewer's suggestion, we now mention randomization upfront in the beginning of the section. We state, "For each age group (3-year-olds, 4-year-olds, etc.), approximately 70 children were tested, with half randomly assigned to respond under time pressure and half to respond under time delay". We have also added a statement about whether blinding was used.

The figure states that the partners were "fictitious", but the method section describes them as real partners. The Appendix adds to the confusion as it states that "the 3 partners are not real children but small closed boxes with images of children's faces" — which presumably is a description and not something that the child participants were told. There is some video recording of at least one child, but was there also an assessment that children believed the children were real? For the moral dilemma task, we are apparently dealing with purely hypothetical scenarios or imagined others. All of this needs to be sorted out and described more clearly. It should also be addressed to what extent children believed they were interacting with real children, including empirical data.

We now clarify upfront in the Method section that children "played with fictitious participants" (p. 14). When introducing the first task, the PGG, we explicitly state that "children were matched with three fictitious child participants (depicted with drawings on small boxes, as shown in Figure 1)". Figure 1 itself has been updated to clearly display the materials presented to children, thereby resolving potential confusion caused by the original figure, which depicted images of real partners. In describing the moral dilemmas (p. 17), we now specify that "participants did not play with partners as in the other tasks." Although we did not directly collect data on whether children believed the partners were real, experimenters were instructed to reassure any child expressing doubts by stating that the other children were real and came from a nearby school where the same tasks were being administered. The use of fictitious partners presented as real is, importantly, a well-established and widely adopted procedure in research with children of the same age group (e.g., Engelmann, Herrmann, & Tomasello, 2018; Beeler-Duden, Pelletz, & Vaish, 2022; Shaw & Olson, 2014).

- Figure 1 is helpful in schematically representing the different tasks. I was confused about the insertion of "boxes used to depict the partners in all games". The schematic drawing might be more effective if it featured samples of the actual stimuli rather than the current old-fashioned representations of what look like white boys with buzz cuts from the 1950s.

We have updated Figure 1 to include the actual stimuli and to enhance overall clarity.

Revision checklist for Nature Human Behaviour manuscripts

General: Refrain from making novelty or priority claims (e.g., 'novel', 'first', 'unprecedented', etc.). Do not use qualitative characterisations of their own work (e.g., 'highly rigorous', 'robust', 'compelling' etc.

We have reviewed the manuscript and can confirm that there are no such instances present.

We ask that studies reporting correlational (rather than causal) evidence use language of association rather than causation to describe their findings. For instance, language such as 'the effect/impact of x on y'; 'x drives x'; 'x leads to a change in y' and so on, is inappropriate when the evidence is correlational. In all such cases, 'x correlates/is associated with y' or similar should be used.

We polished the narrative to avoid any causal language when discussing correlational results.

For studies reporting statistically null results, make sure that you do not interpret the absence of evidence as evidence of absence. There is no statistical test that can demonstrate the absence of an effect. Statements such as 'There is no association between x and y.' or 'X has no effect on Y.' must be revised to read '[No/Little] credible evidence of an association between x and y.' or '[No/Little] credible evidence that X affects Y.'

We have checked the paper, and can confirm we do not have any such instances.

Ensure that your main text does not recycle text from previous publications without proper acknowledgment and citation of the original work.

We have checked the paper, and can confirm we do not have any such instances.

*Title: Up to 150 characters (including spaces); and *not* in a "title:subtitle" format*

Our previous title had a title:subtitle format, we now provide a title not in such a format.

Abstract: Up to 150 words.

The draft is in compliance.

Main text: The main text of your manuscript (i.e., the introduction, results and discussion, excluding the Methods and all other elements, such as abstract, references, figure legends, tables, etc.) should not exceed 5000 words.

The draft is in compliance.

Discussion: The discussion section must include a transparent discussion of limitations.

We discuss limitations openly on page 13.

Statistics: Frequentist inferential statistics should be reported in full wherever they occur (main text, Figure captions, Tables) as follows:

- statistic(degrees of freedom) = value, p = value, effect size statistic = value, % Confidence Intervals = values
- P values reported should be exact. Do not report 'ns' – give the exact value.

The draft is in compliance. When discussing regression coefficients, we report parameter estimates, SE and p-values as customary in frequentist inferential statistics with large datasets. We would be happy to add CIs instead of SEs if preferred. Effect size is not applicable to all regression coefficients. Please let us know of any additional detail required.

All statements or interpretations of the results should be supported by appropriate, fully reported statistics. This extends to comparisons of relationships between variables – for example, interpretations pertaining to difference of differences are also supported by appropriate statistics. For example, if comparison A is statistically significant and comparison B is not, it does not mean that the difference

between A and B is itself statistically significant. If you would like to make claims about difference, A and B must be tested directly.

We verified the narrative to ensure compliance.

Results that do not reach conventional thresholds of statistical significance are not statistically significant. Do not discuss these results as 'marginally significant' or 'just missing significance' and do not interpret these results as providing support for the hypotheses tested.

The draft is now in compliance.

If your manuscript reports any null results and you provide interpretations of these results, you must also report Bayes Factors or equivalence tests. If the main results of your manuscript are null, in addition to Bayes Factors or equivalence tests, we require that you provide statistical evidence that the study is sufficiently powered to detect the smallest theoretically or practically meaningful effect.

We have computed and reported Bayes factors for all null effects.

For manuscripts that use Bayesian statistics, we require a description of the prior specification (i.e., the type of distribution and its parameter values). For informed priors, please provide a justification for the choices made. For hypothesis-testing analyses, please provide both the Bayes Factor and a sensitivity analysis of the Bayes factor under different prior specifications. For parameter estimation, please report summary values and include credible intervals, as well as a plot that includes the posterior distribution, prior distribution, Bayes factor, credible intervals, and posterior median. For more guidance on reporting the results of Bayesian statistical analyses, please see Kruschke 2021 and van Doorn et al. 2020.

The draft is in compliance.

Methods: There is no word limit for the Methods section. It should include all details necessary for reproducing your results.

The draft is in compliance.

If your manuscript reports the results of research with human participants, the Methods section should start with a statement:
a) confirming that your research complies with all relevant ethical regulations;
b) naming the board and institution that approved the study protocol;
c) confirming that informed consent was obtained from all human participants;
d) disclosing information on participant compensation;
e) indicating whether any, all, or none of your studies were preregistered.

We have added the missing information, including information about participant compensation and pre-registration (see pp. 13-14).

Your Methods section should include a statement indicating how the sample sizes were chosen. If power analysis or Bayesian sampling was used, provide full details. If you did not use a formal method for determining sample size, please use the following statement: "No statistical methods were used to pre-determine sample sizes but our sample sizes are [similar to/larger than] those reported in previous publications (ref x,y,z)."

The draft discusses the a-priori sample size analysis and its justification (p. 14).

For experimental studies, the Methods section must include a statement on randomization and whether blinding was used.

The draft now includes these statements in sections 4.1 and 4.2 (p. 14).

Data availability: Your data availability section should inform readers about the availability of the data used to support the conclusions of your study. We strongly encourage authors to publicly share their data unless there are ethical or legal restrictions that prevent you from sharing. If DOIs are provided,

we also strongly encourage including these in the Reference list (authors, title, publisher (repository name), identifier, year). For more guidance on how to write this section please see: http://www.nature.com/authors/policies/data/data-availability-statements-data-citations.pdf

The draft now contains a data availability statement saying: “The dataset generated and analysed during the current study is available in the Open Science Framework repository: https://osf.io/xpsqe/?view only=7a42054dcebd47ef82fd215755485eeb.” (p. 18).

Acknowledgements: Please indicate all sources of funding that supported your work. If no specific funding supported the work, include the following statement: “The authors received no specific funding for this work.” For work that was supported by a funder, indicate what role the funder(s) had in the conceptualization, design, data collection, analysis, decision to publish, or preparation of the manuscript. If any of this information could be perceived as a competing interest, ensure that it is also included in your competing interests statement. If the funder(s) had no role, please include the following statement: “The funders had no role in study design, data collection and analysis, decision to publish or preparation of the manuscript.”

A statement was added as requested (p. 18).

Author contributions: Include an ‘Author contributions’ statement of individual author contributions to the paper (such as experimental work, project planning, data analysis, etc.). The statement should be short and refer to authors by their initials.

The draft is in compliance (p. 18).

Competing interests: We ask authors to declare both financial and non-financial competing interests. If you have no financial or non-financial competing interests, please use the following standard statement: “The authors declare no competing interests.”

The statement was added on p. 18.

Figures and Tables: No more than 8 display items in the main manuscript (more Figures and Tables can be provided as Supplementary Information).

The main manuscript contains only 4 of these.

Data should be presented in a format that shows data distribution (dot-plots or box-and-whisker plots), with all box-plot elements (e.g. center line, median; box limits, upper and lower quartiles; whiskers, 1.5x interquartile range; points, outliers) defined. If bar graphs are used, the corresponding dot plots should be overlaid.

The draft is in compliance.

Graph axes start at zero and have not been altered in scale to exaggerate effects. A ‘discontinuity’ can be used for the y axis if absolutely necessary due to sizing constraints, but the break must be visually evident and should not impinge on any data points.

The draft is in compliance, except for Figure 3 panels a and b. We decided against adding the zero because in sharing games the benchmark level is the equal split which is 2 in our study and included in the plot. We could have normalised observations relative to this level but found the plot more compelling as is, and easier to read and understand for the reader.

References: Within the text, references should appear as superscript Arabic numerals, in order of mention. If a reference is cited more than once, the same number should be used throughout the text and the reference should receive a single entry in the reference list.

The draft has been submitted in standard Nature latex shell, please apply relevant shell.

The reference list should only include

a) articles that have been published or accepted by a named publication;

b) *preprints deposited in a recognized preprint server;*
c) *published conference abstracts;*
d) *numbered patents*
e) *deposited research datasets or code that have been assigned a digital object identifier (DOI).*

We have checked the references list, and the draft is in compliance.

Unpublished manuscripts, submitted manuscripts, and manuscripts under review/revision should not be included in the reference list. If a manuscript is under consideration, not yet submitted or not yet accepted, it should be mentioned in the main text only in parentheses, as follows: (Up to five author names, et al., unpublished manuscript). No major claims can rely solely on unpublished manuscripts or data.

We do not have any such instances.

Ordering of manuscript content: Please make sure that elements of your manuscript are in the following order: Title, Author list, Affiliations, Abstract, Main text, Methods, Data Availability, Acknowledgements, Author Contributions Statement, Competing Interests Statement, Tables, Figure Legends/Captions (for main text figures), References

The draft is in compliance.

Figures must be uploaded individually, one figure per file.

The figures have been uploaded individually.

Reviewer #1

I see several strengths with this study: the authors have identified an important empirical question and used a rigorous methodological and analytic approach with a large (well-justified) sample size. My main questions centre on how this study can inform the predictions of the Social Heuristics Hypothesis and Nativist theories (which serve as the theoretical foundation), and the extent to which the youngest participants understood the more complex tasks, such as the public-goods game.

Theoretical framing

P. 3 In my reading of several SHH papers, there is no explicit developmental prediction, but there is an assumption that heuristics must be acquired through social experience, and so it makes sense that social heuristics would be acquired during early and middle childhood. I understand the logic, offered by the authors, that early social environments within the family may be more supportive of prosocial heuristics compared to the social environments children encounter when entering school, and so the social heuristics acquired may shift from prosocial to self-serving. At the same time, these claims about the type of social environment children are likely to encounter at various developmental stages are not self-evident and so should be made with supporting evidence.

An alternative possibility is that children find themselves in relatively stable social environments (in terms of supporting prosocial or self-serving heuristics) throughout the preschool-to-school-age years. This could lead to developmental stability in social intuitions across the age range studied here, in which case developmental continuity would be equally consistent with the Nativist and SHH accounts. If the acquired heuristics are prosocial, then the same developmental continuity predicted by the authors' in their framing of the nativist account may be observed, in which case it would not be possible to differentiate between the two accounts.

I think it might help to lay out specifically which pattern of evidence in the current study would align with SHH and which would align with a nativist account. A tricky thing about SHH here is that it is hard to imagine a pattern that could falsify it without a way to test alignment between children's social intuition and their social environment. Similarly, most contemporary nativist theories also allow for developmental change after early emergence and a role for social learning, so it is not immediately obvious how these perspectives can be adjudicated based on the pattern of findings in this study. Making explicit predictions about expected patterns would help.

We fully agree with these observations and have rewritten the Introduction in line with the reviewers' suggestions. Specifically, we have shifted the focus from testing predictions and adjudicating between SHH and nativism to a) detailing the development of prosocial intuitions and deliberative social decision-making, and b) clarifying the empirical scope of the SHH and nativist accounts. We now frame the paper as a description of developmental processes that, in turn, help constrain our theoretical frameworks. As the reviewer noted, the SHH allows for at least two possible developmental trajectories: continuity, where prosocial heuristics remain stable across childhood, or transition, where self-serving heuristics emerge in middle childhood. Based on our data, we conclude that prosocial heuristics remain stable up to 10 years of age.

Comprehension for youngest participants

One concern I have is with the level of task comprehension that the youngest participants are likely to have. This seems particularly important when decision-time manipulations are used, as there is a potential for time pressure to exacerbate comprehension problems and, in turn, drive condition effects in unintended ways. I'll focus on the PGG, as this was the most complex of the resource-allocation tasks. The comprehension check verified that children understood that the candies they kept went to themselves and the ones they put in the common bowl went to everyone. But would the three-year-olds have understood the more complex nuances of the PGG? For example, even though the candies are doubled when put in the common bowl, it is better for me to just keep the candies and hope that others contribute if I want to self-maximize. Basically, do they understand that donations to the group are leveraged to provide a larger benefit to the group than keeping the candies can provide to themselves, but that these cooperative decisions are personally costly and the way to self-maximise is to keep everything for yourself? My impression is that the comprehension check showed that participants understood the mechanics of who gets what candies (similar to what would be required for a DG), but

it seems ambiguous whether they would understand these nuances of the PGG. Are there any additional checks that were not fully described in the manuscript? Or is there any precedent for using a PGG with children this young (I'm not aware of any and couldn't identify any in a quick search). It may also help to explain how the evaluation was made that all tasks are age-appropriate for the youngest participants.

We agree that comprehension checks cannot fully capture participants' grasp of all strategic nuances or the optimal play in a task. Comprehension checks were designed to ensure understanding of task mechanics without requiring children to identify the "optimal" strategy or anticipate others' play. For the PGG in particular — the most complex task and one without prior protocols for 3-year-olds — we took extra care by supplementing standard verbal instructions with an additional video demonstration.

Importantly, in the revised version of the article, we include a series of robustness checks that provide three pieces of empirical evidence suggesting that it is unlikely that our results are driven by lack of comprehension. We elaborate on these pieces of evidence below.

(1) Youngest children under time pressure are not more likely to make random errors

We find no systematic tendency for time pressure to move 3–4-year-olds towards random choice. In 5 of the 9 tasks, their behaviour in the fast condition is farther from random choice than their behaviour in the slow condition. For 2 of these 5, a one-sided t-test shows that behaviour in the time pressure condition is significantly farther away from random choice. In UG0 and UG1, behaviour in the time pressure condition is significantly closer to random choice, but that does not extend to the other UG tasks (or any other task). We report on this in Appendix B6 (Table B17).

(2) The main results are robust to excluding youngest children

The main findings remain robust when excluding the youngest children from the analysis. Specifically, excluding 3–4-year-olds from the sample, we find a three-factor structure with factor loadings closely aligned with those from the main specification (Tables B11) and, importantly, we still find a significant interaction condition (fast, slow) x age in the Prosociality factor (Table B12). This suggests that our results are coming from systematic behaviour across all age groups, and not just the youngest children.

(3) The main results are robust to excluding the PGG

We agree with the reviewer that the PGG may have been the most difficult game to understand. To address this concern, we have replicated the analysis omitting all PGG tasks and the associated belief elicitations. Our results are robust to the change, with the factor loadings on the two retained factors closely aligned with those from the main specification (Table B15). Additionally, our estimates for the impact of condition and age on the factors are unchanged (Table B16). This provides reassurance on the validity of our factor approach, which does not appear to rely on specific tasks (i.e., PGG).

Minor points

P. 4 I realise there are significant word constraints for the manuscript, but it might be helpful to provide additional information about the development of intuitive cooperation in past literature. There are additional studies (not cited e.g Grossmann et al., 2020; Chajes et al., 2022, not exhaustive) that have examined this question in the developmental literature, though most use decision-time correlations rather than manipulations. If the authors have decided to selectively cover only studies using decision-time manipulations, that could probably be stated very quickly as the literature is introduced.

Thank you for this comment. We deliberately restricted our review to studies using decision time manipulations, as these provide stronger evidence than correlational studies. Following the reviewer's suggestion, we now acknowledge this point when introducing the literature. Given space constraints, correlational work is mentioned only in passing. This addition appears already in the second paragraph of the Introduction (p. 2).

P. 5 Were judgments about other players' contributions in the PGG made under the decision-time manipulation? If not, does it make sense to look for a condition effect for Social Optimism if the primary factor is not subject to a condition manipulation? I may be missing something here, so would appreciate

clarification.

Judgments were made immediately after contributions, without time pressure. We now clarify this in the Methods (end of p. 15):

> "These judgments were made without time constraints, and children in both conditions had sufficient time to reason about their partners' likely contributions. The goal was to evaluate children's beliefs about how others would behave under time pressure or delay, rather than their own pressured decision making."

We note that examining interactions remains meaningful, as these judgments reflect children's beliefs about decisions made under manipulated cognitive modes. This is now also stated for example on p. 7 in the Results ("assuming that others decided under time pressure or time delay"), but we acknowledge that these beliefs were not directly manipulated.

P. 10 It would be good to mention explicitly that the direction of the relation between PGG contributions and predictions about the contributions of others is not clear, just that there is an association. I think this point is alluded to with "and/or vice versa", but the conclusions are more directional. You could also cite Corbit et al., 2023, Dev Sci; they found a similar association, though only when decisions were made under time pressure.

We fully agree. Following this suggestion, we now discuss the relationship between contributions and expectations about others' contributions in the PGG as an association, without implying a clear causal direction (p. 11, second paragraph). For instance, we note that "children's expectations of high contributions from the partners are positively associated with their own likelihood of contributing" and that "this association may reflect a foundational aspect of human social behaviour: individuals' expectations about others' cooperation and their own willingness to cooperate are closely intertwined, potentially influencing each other through mechanisms such as conditional cooperation." We also cite Corbit's work in this paragraph.

P. 10 I believe SHH would be agnostic about whether intuition favours self-serving or prosocial behaviours in older children. This would depend on the social environment that the older children encounter, and it seems important to show that this environment supports self-serving heuristics in order to claim that SHH would predict this. I may have missed a paper that makes these sorts of predictions from SHH, but if this is based on about the authors' own assertion about children's social environments, then I believe these conclusions could be made more cautiously.

We agree, and we now discuss that SHH can generate multiple predictions depending on the type of social environment children are inserted. Please see p. 4 of the Introduction.

PP. 10–11 I find the claim that "deliberate prosociality rises with age" difficult to interpret because the study's two-condition design (pressure vs. delay) only allows relative rather than absolute inferences. Prior developmental work shows a general increase in prosocial behaviour across this age range, so the upward trend observed in the delay condition could simply mirror that baseline trajectory rather than reveal a unique effect of deliberation. Without a neutral-timing control, we cannot disentangle whether fast decisions elevate prosociality, slow decisions depress it, or both. Once prosociality rates converge across the two conditions at about eight years of age, the data indicate merely that the timing manipulation no longer has a significant effect on behavior (relative to each other, and we don't that what might be happening relative to baseline). In short, the current design more strongly supports conclusions about the relative contributions of intuitive versus deliberative processes, but the separate developmental trajectories for each process are not clear.

Thank you for this useful comment. We have revised the discussion (pp. 11–12) to address these concerns. We use the term *deliberative prosociality* for behaviour observed in the time delay condition, and *intuitive prosociality* for behaviour in the time pressure condition. As we do not include a time neutral condition, we only examine behaviour within and across the two experimental frames. The delay condition explicitly imposes a deliberative mode, allowing a distinct test of prosociality under reflection. We now note on p. 13, while discussing limitations, that the study does not directly address behaviour in a neutral timing context.

Reviewer #2

Major Concerns

1. Theoretical Framing: Nativism vs. Social Heuristics Hypothesis (SHH)

The authors frame their study as a critical test between nativist accounts and the Social Heuristics Hypothesis (SHH), concluding that their findings support nativism while being "more difficult to reconcile" with the SHH. This framing relies on a narrow interpretation of the SHH. The SHH posits that intuitions are heuristics shaped by behaviors that are typically successful in an individual's social environment (Rand et al., 2014). For young children, the primary social environment is the family, where cooperation is generally the most advantageous strategy (Eisenberg et al., 2006; Wentzel, 2014). Therefore, a standard reading of the SHH would predict the internalization of a strong, stable prosocial heuristic from an early age—a prediction that is entirely consistent with the authors' findings.

The authors' argument that the SHH predicts a developmental shift toward selfish intuitions is not a core tenet of the theory and would only hold if school-age environments were so overwhelmingly competitive as to systematically "unlearn" early prosocial heuristics. As such, the study does not provide the critical test it claims. The results are compatible with both a nativist account and an SHH account. The manuscript should be reframed to acknowledge this, shifting the focus from adjudicating between these theories to detailing the novel developmental dynamic between a stable prosocial intuition and emerging deliberative reasoning.

We fully agree with these observations. In response, we have reframed the Introduction and we have clarified that the SHH is consistent with different predictions regarding the emergence (or not) of self-serving intuitions in middle childhood (see p. 4). More generally, the Introduction now frames the study's main aim as describing developmental processes, which are in turn interpreted using the SHH and the nativist account, without creating a contrast within the two. Thus, we shifted the focus from directly testing theoretical predictions to using our data to inform and refine theoretical expectations.

2. Interpretation of Latent Factors

The data-driven factors are a major strength, but their psychological interpretation requires stronger justification. The "Prosociality" Factor: This factor combines cooperation (PGG), altruism (DG), and honesty (DEG) with the acceptance of low offers in the Ultimatum Game (UG). Including the acceptance of unfairness—a lack of costly punishment—in a "Prosociality" factor is conceptually problematic. Prosocial behavior is typically defined as voluntary acts intended to benefit others (Eisenberg et al., 2006), and costly punishment is often considered a key mechanism for maintaining, not undermining, cooperation. An alternative interpretation is that this factor captures "low strategic sophistication" or compliance, which declines with age across all tasks. This concern is amplified by the high uniqueness coefficients for many of the task variables (e.g., PGG2, PGG3, DG2, UG0, UG1) reported in Table B3, which are often above 0.7 or 0.8. High uniqueness suggests that a large portion of a variable's variance is not explained by the common factors, weakening the claim that the factor represents a single, robust underlying trait.

Thank you for this thoughtful comment. We refer to the first factor as "Prosociality", arguing that it captures general prosocial tendencies, specifically defined as behaviours that benefit others (see p. 5, last sentence). In our one-shot context, accepting low offers in the UG does benefit the proposer, and therefore it is a prosocial behaviour according to this definition. Thus, in the context of our one-shot interactions, the Prosociality factor primarily reflects a behaviour that benefits others in the short term, rather than a principled commitment to enforcing fairness norms or a punishment mechanism aimed at increasing long-term cooperation. Importantly, we do not equate this factor with "cooperation". Rather, it represents general prosociality: a willingness to increase others' total payoff in the short term without necessarily knowing or maximizing what is best for the group or society more broadly and in the long term. We clarify this both in the Results and Discussion. We also note that all tasks in our study involved one-shot interactions with putatively different partners, and thus punishments do not influence long-term cooperation. Unlike in repeated interactions, rejecting low offers can only harm others.

As for uniqueness concerns, we agree that high uniqueness values can indicate poor measurement precision. To evaluate the impact of high uniqueness, we conducted a robustness analysis by repeating

our main analyses while omitting tasks with uniqueness coefficients above 0.90. This criterion excluded UG2, MDF, and MDB. Because such an approach excluded the noisiest tasks, factor loadings were largely unaffected, but predicted factors were less noisy. Consequently, our regression analyses yield the same conclusions. Further lowering the threshold to 0.8 excludes 9 of the 19 tasks and the Acquiescence factor no longer achieves an eigenvalue above 1. However, the results for Prosociality and Social Optimism are robust to this threshold as well. To avoid p-hacking the specification, we decided to stick with our original analysis. However, we have now included these robustness checks in Appendix B5 (Table B13). Please also see Table B14 for robustness analysis regressions**.** The exclusion of high-uniqueness variables actually strengthens our findings by reducing measurement noise. The robustness analysis confirms our main results across all three factors. We have only included the 0.9 threshold in the Appendix but can add the 0.8 threshold if requested.

The "Acquiescence" Factor: This factor, defined by accepting all UG offers, is interpreted as a general willingness to accept others' decisions. A more parsimonious explanation is that it reflects the development of task competence. Young children may not fully grasp the strategic nature of the UG, and the age-related decline in this factor may simply track their developing understanding of fairness norms and the power of rejection (McAuliffe et al., 2013).

Thank you for this comment. Please note that our game was one-shot and thus lacked strategic components. We apologize if this was not clear. We now clarify this in the manuscript. Importantly, the "acquiescence" factor loads positively not only on accepting unfair offers (smaller than 2), but also on accepting offers equal to or larger than 2. Since this factor loads positively on accepting *all* types of offers, we interpret it not as reflecting fairness considerations, but rather as a general tendency to accept any offer; hence our use of the term *acquiescence*. Moreover, rejecting advantageous offers is not an indicator of competence, and therefore this pattern works against the alternative interpretation. In the revised version of the article, we discuss potential alternative interpretations in the Discussion.

3. Methodological Validity and Generalizability

Time-Constraint Manipulation: The validity of using a 10-second time pressure manipulation to isolate "intuition" in preschoolers (ages 3-4) is a significant concern. For this age group, the tasks are already cognitively demanding, and time pressure may induce cognitive overload, stress, or confusion rather than preventing deliberation (Ackerman & Lauterman, 2012; Walczyk & Griffith-Ross, 2006).

We agree this is an important concern. In the revised version of the article, we have included several robustness checks that provide three pieces of evidence suggesting that our results are not driven by lack of understanding among youngest children under time pressure.

(1) Youngest children under time pressure are not more likely to make random errors

We compared the results for 3–4-year-olds under time pressure vs time delay, to see which, if any, is further than random. We found no systematic tendency for time pressure to move these participants towards random choice (Table B17). In 5 of the 9 tasks, behaviour in the fast condition is farther from random choice than behaviour in the slow condition. For 2 of these 5, a one-sided t-test shows that behaviour in the time pressure condition is significantly farther away from random choice. In UG0 and UG1, behaviour in the time pressure condition is significantly closer to random choice, but that does not extend to the other UG tasks (or any other task). We report on this in Appendix B6.

(2) The main results are robust to excluding youngest children

Excluding 3–4-year-olds from the sample, we find a three-factor structure with factor loadings closely aligned with those from the main specification (Tables B11) and, importantly, we still find a significant interaction condition (fast, slow) x age in the Prosociality factor (Table B12). This suggests that our results are coming from systematic behaviour across all age groups, and not just the youngest children.

(3) The main results are robust to excluding the PGG

We have replicated the analysis omitting all PGG tasks (arguably the most difficult tasks) and the associated belief elicitations. Our results are robust to the change, with the factor loadings on the two retained factors closely aligned with those from the main specification (Table B15). Additionally, our

estimates for the impact of condition and age are unchanged (Table B16). This provides reassurance on the validity of our factor approach, which does not appear to rely on specific tasks (i.e., PGG).

Furthermore, the literature on time pressure in children is contradictory; while some studies find it increases prosocial sharing (Plötner et al., 2021), others find it inhibits prosocial helping, arguing that helping requires deliberation (Zha et al., 2025). This conflicting evidence must be addressed.

Thank you for pointing this out. We now reference the contrasting findings in the literature on p. 11.

Generalizability: The study was conducted exclusively with children from Milan, Italy, a WEIRD (Western, Educated, Industrialized, Rich, Democratic) society. Given substantial evidence of cross-cultural variation in prosocial development (House et al., 2013; Kärtner, 2015), the authors must temper their broad claims about "human development" and explicitly discuss this limitation.

We have now added this point as a limitation and future direction in the Discussion (see p. 13), and overall we have tempered our writing.

Minor Concerns

Figure Clarity: Figure 2 is central to the paper's claims but is very dense. The main text should guide the reader through the key takeaways from each panel more explicitly.

We now discuss the figure in greater detail throughout sections 2.1 and 2.2.

Comprehension Checks: The authors should report whether comprehension check failure rates differed between the time pressure and time delay conditions, as this would speak directly to the cognitive load imposed by the manipulation.

We now report failure rates for comprehension checks by age and condition in Appendix B1. We note that children under time pressure were not significantly less likely to pass the comprehension questions; if anything, descriptive statistics point in the opposite direction.

This is a potentially high-impact study with a rich dataset and innovative methods. However, the theoretical claims are currently overstated, and the interpretation of the core findings requires significant refinement. The manuscript can be substantially strengthened if the authors reframe the theoretical argument, provide a more nuanced discussion of the latent factors (including alternative interpretations), and thoroughly address the methodological limitations concerning the time-pressure manipulation and cultural specificity.

We are thankful for the comments and suggestions we have received, which have greatly helped us in revising the paper.

Reviewer #3

Strengths: This is quite a large study in terms of the sample size, age-range, and breadth in measures. The topic is of general interest in the behavioral sciences, connecting discussions about adult psychology and developmental psychology (but see some suggestions for improvement below). Despite the number of measures, the results are described succinctly and fairly easy to follow. The authors make good use of the supplemental material. The experimental tasks are adequate and build on previously validated paradigms. Some more information on execution and additional analyses are needed, however.

Weaknesses

The introduction makes distinction between nativist accounts and the Social Heuristics Hypothesis. My two main reservations are that what predictions the Social Heuristics Hypothesis makes regarding the emergence of cooperative behaviors in children as grounded in empirical findings about child development. The paragraph on p. 3 is purely speculative, not making any connection to the developmental literature. The false dichotomy between a social heuristics approach focused on learning and experience and nativist approaches continues. This sounds like the nature-nurture debate long buried and now revived. Note also that the study is not set up to test any claims about early-emerging capacities, given that the youngest children of the sample are already 3 years old (researchers debating this issue test infants and toddlers).

We agree with these observations. In line with similar points raised by the other two reviewers, we have revised the Introduction to avoid framing our study as adjudicating between SHH and nativism, since these theories can, in fact, generate overlapping predictions. Instead, we now present our work primarily as an effort to characterize the development of intuitive and deliberate social decision making, with the additional aim of using our data to refine the empirical scope and possible predictions of the main theoretical frameworks guiding our Introduction.

The second reservation is that more nuance is needed in the introduction of developmental accounts about the origins of prosociality. For one, the authors measure both children's behaviors and their expectations / representations of social partners, which are ontologically different. Some of the cited findings and claims concern what children THINK about cooperative behaviors, how they EVALUATE e.g. helpers and hinders, while others are about what MOTIVATES children's sharing and helping behavior. Please look up discussions of the knowledge-behavior gap in the development of cooperation (e.g. Blake et al.). Related to this, more theoretical nuance is needed to make predictions about what develops in children, i.e. what aspects are innate and what aspects are learned. Warneken et al. has written an Annual Reviews of Psychology article on this, providing a framework that might be helpful.

We also agree with this second concern. We have revised the Introduction, and in the updated paragraph on p. 3 we now structure the literature review around three strands of evidence: (i) studies on expectations about others' behaviour, (ii) studies on preferences or evaluations, and (iii) studies on motivations or behaviours. We also cite the recommended works by Blake et al. and Warneken.

More attention to the extant developmental literature on intuitive vs. deliberate processes is needed. See the work by Corbit et al, and Grossmann et al., which also includes public good games with time manipulation.

Thank you for pointing to this literature. We cite Corbit et al. on p. 2. Assuming the reviewer is referring to Grossmann, Missana, & Vaish (2020), Cognition, 196, 104144, we note that this is not a manipulation study; we thus include it among the studies relying solely on correlational measures (p. 2). The literature paragraph has been expanded in the suggested direction.

The title uses "prosociality" as the umbrella term, but then only one of the tasks is about prosociality. Use "cooperation" instead as the overarching concept?

We would say that only one task strictly addresses "cooperation", namely, the PGG. Importantly, we do not use "Prosociality" as a broad umbrella term, but when capitalised, it specifically refers to the construct identified through our factor analysis. This factor loads on behaviours that increase the others' payoff; these behaviours align with standard definitions of prosociality (Eisenberg & Mussen, 1989),

which is why we chose this term. We have carefully reviewed the manuscript to ensure consistency and now define "prosociality" explicitly at the outset (p. 2, second sentence).

Several questions remain regarding methods and presentation of results:

How were children's responses recorded?

We have added this information to the methods section (p. 14, beginning of section 4.2): "… their responses were recorded by the experimenter using paper and pen."

No information on reliability coding seems to have been provided.

We now clarify in the manuscript that children's responses were recorded by the experimenters using paper and pen (p. 14). During each session, experimenters simply registered the number of candies contributed by each participating child, and no video recordings were made. Because the task involved only a straightforward numerical response and there were no subjective judgments to code, inter-rater reliability was not applicable in this context.

Please state that condition assignment was randomized on p. 12 ("half under time pressure and half under time delay"). This is currently only mentioned for each individual task.

Thank you, the information was already noted at the end of 4.1 section, but following the reviewer's suggestion, we now mention randomization upfront in the beginning of the section. We state, "For each age group (3-year-olds, 4-year-olds, etc.), approximately 70 children were tested, with half randomly assigned to respond under time pressure and half to respond under time delay". We have also added a statement about whether blinding was used.

The figure states that the partners were "fictitious", but the method section describes them as real partners. The Appendix adds to the confusion as it states that "the 3 partners are not real children but small closed boxes with images of children's faces" — which presumably is a description and not something that the child participants were told. There is some video recording of at least one child, but was there also an assessment that children believed the children were real? For the moral dilemma task, we are apparently dealing with purely hypothetical scenarios or imagined others. All of this needs to be sorted out and described more clearly. It should also be addressed to what extent children believed they were interacting with real children, including empirical data.

We now clarify upfront in the Method section that children "played with fictitious participants" (p. 14). When introducing the first task, the PGG, we explicitly state that "children were matched with three fictitious child participants (depicted with drawings on small boxes, as shown in Figure 1)". Figure 1 itself has been updated to clearly display the materials presented to children, thereby resolving potential confusion caused by the original figure, which depicted images of real partners. In describing the moral dilemmas (p. 17), we now specify that "participants did not play with partners as in the other tasks." Although we did not directly collect data on whether children believed the partners were real, experimenters were instructed to reassure any child expressing doubts by stating that the other children were real and came from a nearby school where the same tasks were being administered. The use of fictitious partners presented as real is, importantly, a well-established and widely adopted procedure in research with children of the same age group (e.g., Engelmann, Herrmann, & Tomasello, 2018; Beeler-Duden, Pelletz, & Vaish, 2022; Shaw & Olson, 2014).

- Figure 1 is helpful in schematically representing the different tasks. I was confused about the insertion of "boxes used to depict the partners in all games". The schematic drawing might be more effective if it featured samples of the actual stimuli rather than the current old-fashioned representations of what look like white boys with buzz cuts from the 1950s.

We have updated Figure 1 to include the actual stimuli and to enhance overall clarity.

Revision checklist for Nature Human Behaviour manuscripts

General: Refrain from making novelty or priority claims (e.g., 'novel', 'first', 'unprecedented', etc.). Do not use qualitative characterisations of their own work (e.g., 'highly rigorous', 'robust', 'compelling' etc.

We have reviewed the manuscript and can confirm that there are no such instances present.

We ask that studies reporting correlational (rather than causal) evidence use language of association rather than causation to describe their findings. For instance, language such as 'the effect/impact of x on y'; 'x drives x'; 'x leads to a change in y' and so on, is inappropriate when the evidence is correlational. In all such cases, 'x correlates/is associated with y' or similar should be used.

We polished the narrative to avoid any causal language when discussing correlational results.

For studies reporting statistically null results, make sure that you do not interpret the absence of evidence as evidence of absence. There is no statistical test that can demonstrate the absence of an effect. Statements such as 'There is no association between x and y.' or 'X has no effect on Y.' must be revised to read '[No/Little] credible evidence of an association between x and y.' or '[No/Little] credible evidence that X affects Y.'

We have checked the paper, and can confirm we do not have any such instances.

Ensure that your main text does not recycle text from previous publications without proper acknowledgment and citation of the original work.

We have checked the paper, and can confirm we do not have any such instances.

*Title: Up to 150 characters (including spaces); and *not* in a "title:subtitle" format*

Our previous title had a title:subtitle format, we now provide a title not in such a format.

Abstract: Up to 150 words.

The draft is in compliance.

Main text: The main text of your manuscript (i.e., the introduction, results and discussion, excluding the Methods and all other elements, such as abstract, references, figure legends, tables, etc.) should not exceed 5000 words.

The draft is in compliance.

Discussion: The discussion section must include a transparent discussion of limitations.

We discuss limitations openly on page 13.

Statistics: Frequentist inferential statistics should be reported in full wherever they occur (main text, Figure captions, Tables) as follows:

- statistic(degrees of freedom) = value, p = value, effect size statistic = value, % Confidence Intervals = values
- P values reported should be exact. Do not report 'ns' – give the exact value.

The draft is in compliance. When discussing regression coefficients, we report parameter estimates, SE and p-values as customary in frequentist inferential statistics with large datasets. We would be happy to add CIs instead of SEs if preferred. Effect size is not applicable to all regression coefficients. Please let us know of any additional detail required.

All statements or interpretations of the results should be supported by appropriate, fully reported statistics. This extends to comparisons of relationships between variables – for example, interpretations pertaining to difference of differences are also supported by appropriate statistics. For example, if comparison A is statistically significant and comparison B is not, it does not mean that the difference

between A and B is itself statistically significant. If you would like to make claims about difference, A and B must be tested directly.

We verified the narrative to ensure compliance.

Results that do not reach conventional thresholds of statistical significance are not statistically significant. Do not discuss these results as 'marginally significant' or 'just missing significance' and do not interpret these results as providing support for the hypotheses tested.

The draft is now in compliance.

If your manuscript reports any null results and you provide interpretations of these results, you must also report Bayes Factors or equivalence tests. If the main results of your manuscript are null, in addition to Bayes Factors or equivalence tests, we require that you provide statistical evidence that the study is sufficiently powered to detect the smallest theoretically or practically meaningful effect.

We have computed and reported Bayes factors for all null effects.

For manuscripts that use Bayesian statistics, we require a description of the prior specification (i.e., the type of distribution and its parameter values). For informed priors, please provide a justification for the choices made. For hypothesis-testing analyses, please provide both the Bayes Factor and a sensitivity analysis of the Bayes factor under different prior specifications. For parameter estimation, please report summary values and include credible intervals, as well as a plot that includes the posterior distribution, prior distribution, Bayes factor, credible intervals, and posterior median. For more guidance on reporting the results of Bayesian statistical analyses, please see Kruschke 2021 and van Doorn et al. 2020.

The draft is in compliance.

Methods: There is no word limit for the Methods section. It should include all details necessary for reproducing your results.

The draft is in compliance.

If your manuscript reports the results of research with human participants, the Methods section should start with a statement:
a) confirming that your research complies with all relevant ethical regulations;
b) naming the board and institution that approved the study protocol;
c) confirming that informed consent was obtained from all human participants;
d) disclosing information on participant compensation;
e) indicating whether any, all, or none of your studies were preregistered.

We have added the missing information, including information about participant compensation and pre-registration (see pp. 13-14).

Your Methods section should include a statement indicating how the sample sizes were chosen. If power analysis or Bayesian sampling was used, provide full details. If you did not use a formal method for determining sample size, please use the following statement: "No statistical methods were used to pre-determine sample sizes but our sample sizes are [similar to/larger than] those reported in previous publications (ref x,y,z)."

The draft discusses the a-priori sample size analysis and its justification (p. 14).

For experimental studies, the Methods section must include a statement on randomization and whether blinding was used.

The draft now includes these statements in sections 4.1 and 4.2 (p. 14).

Data availability: Your data availability section should inform readers about the availability of the data used to support the conclusions of your study. We strongly encourage authors to publicly share their data unless there are ethical or legal restrictions that prevent you from sharing. If DOIs are provided,

we also strongly encourage including these in the Reference list (authors, title, publisher (repository name), identifier, year). For more guidance on how to write this section please see: http://www.nature.com/authors/policies/data/data-availability-statements-data-citations.pdf

The draft now contains a data availability statement saying: "The dataset generated and analysed during the current study is available in the Open Science Framework repository: https://osf.io/xpsqe/?view only=7a42054dcebd47ef82fd215755485eeb." (p. 18).

Acknowledgements: Please indicate all sources of funding that supported your work. If no specific funding supported the work, include the following statement: "The authors received no specific funding for this work." For work that was supported by a funder, indicate what role the funder(s) had in the conceptualization, design, data collection, analysis, decision to publish, or preparation of the manuscript. If any of this information could be perceived as a competing interest, ensure that it is also included in your competing interests statement. If the funder(s) had no role, please include the following statement: "The funders had no role in study design, data collection and analysis, decision to publish or preparation of the manuscript."

A statement was added as requested (p. 18).

Author contributions: Include an 'Author contributions' statement of individual author contributions to the paper (such as experimental work, project planning, data analysis, etc.). The statement should be short and refer to authors by their initials.

The draft is in compliance (p. 18).

Competing interests: We ask authors to declare both financial and non-financial competing interests. If you have no financial or non-financial competing interests, please use the following standard statement: "The authors declare no competing interests."

The statement was added on p. 18.

Figures and Tables: No more than 8 display items in the main manuscript (more Figures and Tables can be provided as Supplementary Information).

The main manuscript contains only 4 of these.

Data should be presented in a format that shows data distribution (dot-plots or box-and-whisker plots), with all box-plot elements (e.g. center line, median; box limits, upper and lower quartiles; whiskers, 1.5x interquartile range; points, outliers) defined. If bar graphs are used, the corresponding dot plots should be overlaid.

The draft is in compliance.

Graph axes start at zero and have not been altered in scale to exaggerate effects. A 'discontinuity' can be used for the y axis if absolutely necessary due to sizing constraints, but the break must be visually evident and should not impinge on any data points.

The draft is in compliance, except for Figure 3 panels a and b. We decided against adding the zero because in sharing games the benchmark level is the equal split which is 2 in our study and included in the plot. We could have normalised observations relative to this level but found the plot more compelling as is, and easier to read and understand for the reader.

References: Within the text, references should appear as superscript Arabic numerals, in order of mention. If a reference is cited more than once, the same number should be used throughout the text and the reference should receive a single entry in the reference list.

The draft has been submitted in standard Nature latex shell, please apply relevant shell.

The reference list should only include

a) articles that have been published or accepted by a named publication;

b) preprints deposited in a recognized preprint server;
c) published conference abstracts;
d) numbered patents
e) deposited research datasets or code that have been assigned a digital object identifier (DOI).

We have checked the references list, and the draft is in compliance.

Unpublished manuscripts, submitted manuscripts, and manuscripts under review/revision should not be included in the reference list. If a manuscript is under consideration, not yet submitted or not yet accepted, it should be mentioned in the main text only in parentheses, as follows: (Up to five author names, et al., unpublished manuscript). No major claims can rely solely on unpublished manuscripts or data.

We do not have any such instances.

Ordering of manuscript content: Please make sure that elements of your manuscript are in the following order: Title, Author list, Affiliations, Abstract, Main text, Methods, Data Availability, Acknowledgements, Author Contributions Statement, Competing Interests Statement, Tables, Figure Legends/Captions (for main text figures), References

The draft is in compliance.

Figures must be uploaded individually, one figure per file.

The figures have been uploaded individually.
